# What are the experiences of supportive care in people affected by brain cancer and their informal caregivers: A qualitative systematic review

**DOI:** 10.1007/s11764-023-01401-5

**Published:** 2023-05-31

**Authors:** C. Paterson, C. Roberts, J. Li, M. Chapman, K. Strickland, N. Johnston, E. Law, R. Bacon, M. Turner, I. Mohanty, G. Pranavan, K. Toohey

**Affiliations:** 1 Faculty of Health, University of Canberra, Bruce, Canberra, ACT Australia; 2 Prehabilitation, Activity, Cancer, Exercise and Survivorship (PACES) Research Group, University of Canberra, Bruce, Canberra, ACT Australia; 3 School of Nursing, Midwifery and Public Health, University of Canberra, Bruce, Canberra, ACT Australia; 4 Canberra Health Services and ACT Health, Garran, Canberra, Australia; 5 Robert Gordon University, Aberdeen, Scotland, UK; 6 Department of Palliative Care, Canberra Health Services, Garran, Canberra, Australia; 7 School of Medicine and Psychology, Australian National University, Canberra, Australia; 8 School of Nursing & Midwifery, Edith Cowan University, Joondalup, WA Australia; 9 School of Clinical Sciences, Faculty of Health and Environmental Sciences, AUT, Auckland, New Zealand; 10 Icon Cancer Centre, Canberra, Australia

**Keywords:** Qualitative, Systematic review, Brain cancer, Supportive care, Patients, Informal caregivers

## Abstract

**Purpose:**

To critically synthesise qualitative research to understand experiences of supportive care in people affected by brain cancer and their informal caregivers.

**Methods:**

A qualitative systematic review was conducted according to the Joanna Briggs methodology and has been reported according to the Preferred Reporting Items for Systematic Reviews and Meta-Analysis (PRISMA) Guidelines. Electronic databases were searched by an expert systematic review librarian for all qualitative studies irrespective of research design. All publications were double screened by two reviewers using a pre-determined exclusion and inclusion criteria. The review was managed using Covidence systematic review software. Methodological quality assessment and data extraction were performed. Qualitative findings accompanied by illustrative quotes from included studies were extracted and grouped into categories, which created the overall synthesised findings.

**Results:**

A total of 33 studies were included which represented a total sample of 671 participants inclusive of 303 patients and 368 informal caregivers. There was a total of 220 individual findings included in this review, which were synthesised into two findings (1) caregivers and patients perceived supports which would have been helpful and (2) caregiver and patient experiences of unmet supportive care needs.

**Conclusion:**

This review highlighted the suffering and distress caused by brain cancer and associated treatments. Both patients and their informal caregivers experienced disconnect from themselves in renegotiating roles, and a profound sense of loneliness as the physical deterioration of the disease progressed. Both patients and informal caregivers reported similar unmet needs within the current service provision for brain cancer. However, what is apparent is that current cancer services are provided solely for patients, with little or no consideration to the support needs of both the patient and their informal caregiver. Service re-design is needed to improve care coordination with individualised informational support, implementation of holistic needs assessments for both the patients and their caregivers, better community support provision, improved opportunities for emotional care with early referral for palliative care services.

**Implications for cancer survivors:**

It is recommended that members of the multidisciplinary brain cancer team reflect on these findings to target holistic needs assessments and develop shared self-management care plans for both the patient and the informal caregiver.

**Supplementary Information:**

The online version contains supplementary material available at 10.1007/s11764-023-01401-5.

## Introduction

Primary malignant brain tumours (PMBT) are comparatively rare and account for 1.7% of all cancers with a global incidence of 3.9 per 100,000 [1]. The most common variant in adults are high-grade gliomas, which result in a disproportionately high level of morbidity and mortality, with a median survival rate of 12–15 months [2]. Treatment modalities [3] include chemotherapy, radiotherapy and/or surgery which often results in severe long-term side effects [4], which negatively impacts quality of life [5]. Physical symptoms are common in PMBT and often require treatment. Frequently needed symptom control includes antiemetics to control nausea, anti-seizure medications to control symptoms, analgesia for pain and steroids to reduce the brain swelling [6]. Importantly, unlike individuals with other terminal cancer diagnoses, people diagnosed with PMBT are likely to have physical and cognitive deficits from the time of diagnosis, due to tumour invasion of the delicate tissues in the brain. People affected by PMBT often experience significant negative physical and psychological consequences of the cancer itself and associated treatments. Many people diagnosed with PMBT can experience changes in personality, behaviour, mood, weight changes loss of cognitive function, lack of control of bodily functions, sensory loss, loss of mobility, impaired speech, visual-perception deficits, seizures, fatigue, loneliness, social isolation, anxiety and depression [7, 8]. Additionally, people living with PMBT often grapple with indirect consequences, such as changes to their family life, economic situation, occupational and social roles and independence due to their inability to legally drive a motor vehicle [8]. Caregivers of individuals with PMBT also face significant and unique circumstances in relation to emotional care and physical burden, which can reduce their own quality of life [9]. As the disease progresses and symptoms become more problematic, patients become increasingly reliant on their informal caregivers for support with all activities of daily living, as well as social, emotional, spiritual, and financial support.

A previous systematic review [10] identified only eleven qualitative studies during 2005–2011 that reported on aspects of follow-up and supportive care for people diagnosed with brain cancer. There are several limitations of this review [10]; firstly, this systematic review is outdated clinically by year of publication (2012). Secondly, there were methodological limitations, namely, the reviewers did not provide a transparent account of the process of data synthesis, nor did they provide the quality assessment of the included studies. Consequently, the methodological quality of the evidence presented in this review is unclear and therefore problematic in the transferability of this evidence to practice. Given the changing clinical landscape since publication of the review [10], it is timely to understand contemporary supportive care experience from the patients and their nominated caregiver.

Supportive care is broadly defined as the necessary cancer services for those affected by cancer to meet their person-centred physical, emotional, social, psychosocial, informational, spiritual and practical needs during diagnosis, treatment and follow-up phases, encompassing issues of survivorship, palliative care and bereavement [11]. Given the reported experiences of unmet supportive care needs of people affected by brain cancer [12–14] and their caregivers [15, 16], it is important to critically synthesise recent existing evidence to identify the domains of unmet supportive care needs. Therefore, this systematic review aimed to inform holistic rehabilitation person-centred models of care, to develop evidence-based clinical guidelines, informed from insights on the experiences of patients and caregivers, in their own words. This qualitative systematic review addresses the following research questions:

1. What supports were perceived as beneficial among people affected by brain cancer and their informal caregivers?
2. What are the unmet supportive care needs among people affected by brain cancer and their informal caregivers?

## Method

### Design

This systematic review has been reported according to the Preferred Reporting Items for Systematic Reviews and Meta-Analyses (PRISMA) guidelines [17]. A meta-aggregation of qualitative studies [18] was conducted to identify and synthesise qualitative research studies, to understand the experiences, needs and preferences for supportive care, among people diagnosed with primary brain cancer and their informal caregivers. This review was conducted according to a priori systematic review protocol available upon request.

### Pre-eligibility screening criteria

#### Types of studies

- Studies exploring experiences, needs and preferences for supportive care in participants diagnosed with brain cancer, and their informal caregivers
- Qualitative studies only irrespective of research design and qualitative components of mixed methods studies
- Relevant systematic reviews were scrutinised for potentially relevant studies for screening
- Studies conducted with adults (≥ 18 years old) and informal caregivers

### Exclusion criteria

- All quantitative studies, conference abstracts, commentaries, editorials or studies which did not provide data to address the research question.

#### Types of participants

- Adults (≥ 18 years of age) with a confirmed histological diagnosis of primary brain cancer irrespective of stage of disease or treatment, and their informal caregivers. Participants with thyroid cancers and brain metastasis were excluded.

#### Types of outcomes measures

Qualitative experiences, needs and preferences for supportive care (e.g. qualitative experiences) based upon the classification of supportive care [11].

### Search strategy

Searches to identify relevant publications were conducted by an expert academic librarian using a combination of keywords and subject headings. Search terms were applied consistently across the APA PsycINFO, CINAHL, Cochrane Library (Database of Systematic Reviews and Central Register of Controlled Trials), Medline, Proquest (Nursing and Allied Health Database, Health and Medical Collection), and Scopus databases. See Supplementary Table 1 for the full record of searches.

### Study selection

Following the search, all identified citations were imported into Covidence systematic review software for de-duplication and screening according to the inclusion and exclusion criteria. Titles and abstracts were screened by nine reviewers (CP, GP, JL, EL, MC, KS, RB, NJ, KT), with any conflicts resolved by discussion. The full texts of selected studies were retrieved and assessed in detail against the inclusion criteria by nine reviewers (GP, CP, KS, JL, KT, NJ, MC, EL, RB). Full-text studies that did not meet the inclusion criteria were excluded and reasons for exclusion provided. The study selection process is described using the PRISMA flow diagram [17].

### Assessment of methodological quality

All studies meeting the inclusion criteria were assessed using the JBI Critical Appraisal Checklist for Qualitative Research. This is a 10-item Critical Appraisal Checklist which assesses congruity between the philosophical/theoretical position adopted in the study, study methodology, study methods, the research question, the representation of the data and the interpretation of the findings of each of the selected studies [18]. The item ratings of each appraisal were consolidated and represented in a final quality appraisal table. The included studies were assigned a score based on each question within the appraisal tool, with a rating of yes, no, or unclear.

### Data extraction

The data extracted across the included studies capture information about the population, context, geographical location, study methods and the phenomena of interest relevant to the research question. Qualitative themes as highlighted by the study authors of the included studies provided textual findings to provide representability of the original study. The findings were extracted directly from the studies, and illustrative quotations were extracted to illustrate each finding. Importantly, the reviewers extracted the findings as reported by the researchers of each included study, without interpreting the actual data in keeping with the JBI meta-aggregation method [18].

### Data synthesis

Qualitative research findings (subthemes and illustrative quotes) across the included studies were synthesized using a thematic analysis approach. Specifically, the synthesis of findings enabled the generation of a set of statements that represented similar findings which were categorized based on the commonality of meaning [18]. Findings and supporting illustrations were assessed for congruence and were given a ConQual ranking of either ‘unequivocal’ (clear association between the finding and illustration), ‘credible ’ (unclear association between the finding and illustration, leaving it open to challenge) or ‘not supported’ (findings not supported by data) [18]. Unsupported findings were not included in the final synthesis in keeping with the JBI methodology. Following careful and repeated assessment of the compiled data, two or more findings were grouped into categories and then were grouped together to form overall synthesised findings.

The data synthesis involved three steps in this process:

- Step 1: The data extraction (findings and illustrative quotes) from the main findings of the original studies was extracted in tabular format.
- Step 2: The findings and associated illustrative quotes were grouped together based on similar meaning.
- Step 3: The final step in the meta-aggregation synthesis involved the generation of categories and the final synthesized findings reviewing conclusions with primary sources.

This process in the data synthesis was carried out by one reviewer and quality checked by a second reviewer. Any disagreements were resolved by discussion.

### Findings

**Fig. 1:**
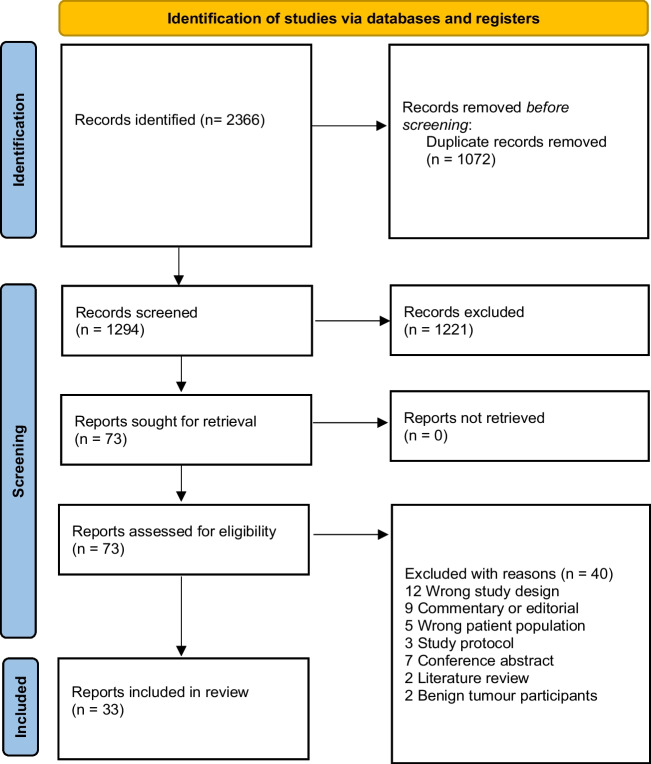
PRISMA flow diagram

Of the 1294 publications screened, 73 full-text articles were assessed according to the pre-eligibility criteria, and 40 were excluded with reasons; see Fig. 1. A total of 33 studies met the inclusion criteria. The studies were conducted in a range of countries which included United Kingdom (*n* = 7), Netherlands (*n* = 1), multi-country study (*n* = 2), Australia (*n* = 7), Belgium (*n* = 2), Canada (*n* = 1), Sweden (*n* = 1), USA (*n* = 7), Denmark (*n* = 4) and Germany (*n* = 1); see Table 1 for an overview of the included studies. This systematic review represented a total sample of 671 participants inclusive of 303 patients and 368 informal caregivers, noting that one study did not report on sample size [31]. Overall, the methodological quality of the included studies was good but with the notable exception of a lack of reporting of the researchers theoretical positioning and acknowledgement of the researcher influences on the study data; see Table 2 for results of quality assessment of the included studies.

**Table 1:** Characteristics of the included studies

Study and country	Methods for data collection and analysis	Phenomena of interest	Setting/context/culture	Participant characteristics and sample size	Description of main findings
Arber et al. [9] UK - England	In-depth qualitative interviews using grounded theory	Carer’s access to and experience of information/support	One specialist hospital in South of England	22 caregivers	Challenging experience with gaps in information provided. Main areas of difficulty were combining employment and caring, managing finances and benefits, locating support groups, what to expect following neurosurgery, managing medications.
Arber et al. [19] UK – England *Reporting same study as Aber et al. [9]	In-depth qualitative interviews using grounded theory	Experience of family caregivers when caring for a person with primary malignant brain tumour	One cancer centre in Southeast England	22 caregivers	The themes generated were those of developing helpful relationships, safe places, comfort zones, and threats to connecting.
Boele et al. [20] Netherlands	Individual semi-structured interviews that were audiotaped	Patients’ and caregivers’ attitudes and preferences toward symptoms and distress monitoring	One outpatient oncology department	15 patients and 15 informal caregivers	Advantages of monitoring generated by participants include increased awareness of problems and facilitating supportive care provision. Disadvantages included investment of time and mastering the discipline to monitor frequently.
Boele et al. [21] USA and Netherlands *Includes same sample as Boele et al. [20]	Individual semi-structured interviews that were audiotaped	Explore PBT caregivers’ preferences toward symptoms and distress monitoring	One Cancer Centres in USA and one cancer centre in the Netherlands	USA 12 caregivers Dutch 15 caregivers	Caregivers utilize both formal and informal support services. Keeping track of care issues was thought to provide more insight into unmet needs and help them find professional help, but it requires investment of time and takes discipline.
Cavers et al. [22] UK - Scotland	Prospective longitudinal qualitative interviews using grounded theory	Explore the multidimensional experience of patients and caregivers	A tertiary centre of clinical neurosciences	26 patients, 23 caregivers	Physical, social, psychological, and existential distress even before a diagnosis was confirmed. Social decline followed a similar trajectory to that of physical decline, whereas psychological and existential distress were typically acute around diagnosis and again after initial treatment.
Collins et al. [23] Australia	Individual semi-structured interviews that were audiotaped	To understand the supportive and palliative care needs	Neurosurgery, oncology and palliative care services of two Australian metropolitan hospitals	23 caregivers (15 current and 8 bereaved)	Carers described significant needs in relation to three distinct domains: the challenge of caring; the lack of support available to carers and the suffering of caring. The need for care coordination to improve care.
Coolbrandt et al. [24] Belgium	Qualitative interviews using grounded theory	Explore the experience of informal caregivers	Oncology wards of the University Hospital	16 caregivers	The overall theme related to experiences of family caregivers this the following sub-themes, feeling lost and alone in a new life, committed but struggling to care, and caring needs.
Cubis et al. [25] Australia	Qualitative phenomenological study. Two in-depth semi-structured interviews were conducted three months apart	Aimed to understand how brain tumour influences people’s ability to manage, maintain, and rebuild their social networks	Patients at different stages of cancer interviewed in their own homes or other locations that were convenient for them and offered privacy	20 patients with diverse types of primary brain tumours	Two overarching and interrelated themes emerged: engaging and connecting and then versus now. An interplay of barriers, facilitators and strategies influenced people’s ability to engage and connect with their social groups, which in turn influenced whether they experienced stability; maintenance and expansion; loss and rebuilding; or loss and shrinkage of their social networks over time.
D’Agostino and Edelstein [26] Canada	Four focus groups	Explore needs of young adult PMBT survivors	Oncology wards of the University Hospital	7 young adult survivors	Common challenges across the groups included physical appearance, fertility, late effects, social relationships, and changing priorities. Childhood cancer survivors struggled with identity formation, social isolation, and health care transitions.
Dahlberg et al. [27] Sweden	An exploratory qualitative study. In-depth interviews were conducted and a social network-mapping tool (CareMaps) was tested	Explores how patients and informal caregivers perceive the potential usefulness of a social network-mapping tool in their self-care and to describe the qualities in the interpersonal relations that they map	Study participants were recruited via a series of workshops facilitated by the designer of the CareMaps tool	7 persons living with brain tumours, 12 informal caregivers (where of 6 bereaved)	Participants expressed positive opinions about the CareMaps tool but raised some questions regarding its design, how to use it in their self-care, and the optimal timing of introducing the tool. Two themes reflecting qualities in relations were found: self-care supportive relations during which daily management of the brain tumour is in focus and identity-preserving relations that allow individuals to disconnect from their brain tumour experiences. Both types of relations were described as important, were found in different contexts (e.g., social life, work life, and healthcare), and emphasized contrasting qualities.
Deatrick et al. [28] USA	Sequential, mixed-methods design	To explore a typology of family management (FM) patterns for young adult survivors	Neuro-oncology and survivorship outpatient clinics	45 mothers (involved in qualitative phase)	Need related to having successful strategies to incorporate changes in survivor functioning into everyday family life, profound stress related to daily challenges and families were able to manage, accommodate, and accept differences.
Foust Winton et al. [29] USA	A qualitative descriptive method study using semi-structured interviews	Describes how patients who have undergone craniotomy for brain tumour removal experience pain management while hospitalised	Interviews conducted with patients on a neurological step-down unit in an urban teaching hospital in the Midwest United States	27 patients who had undergone a craniotomy 2 weeks prior	Their pain experiences varied on 2 dimensions: salience of pain during recovery and complexity of pain management. Based on these dimensions, 3 distinct types of pain management experiences were identified: (1) pain-as-nonsalient, routine pain management experience; (2) pain-as-salient, routine pain management experience; and (3) pain-as-salient, complex pain management experience. Many post craniotomy patients experience their pain as tolerable and/or pain management as satisfying and effective; others experience pain and pain management as challenging.
Francis et al. [30] Denmark	Individual semi-structured interviews, over two time points, which were recorded	To investigate spouses’ experiences of suffering in their role as main caregiver of a partner with PMBT	Oncology ward of a university hospital	10 spouse caregivers (7 women and 3 men)	Three central themes: 1) “enduring everyday life”, 2) “being overlooked and hurt” and 3) “being acknowledged and feeling good”. Spouse caregivers are suffering from exhaustion and supress their own emotions to endure care responsibilities. Overlooking their experiences and everyday hardship causes disappointment and hurts their dignity. Acknowledgment through simple acts of practical help or time to talk are consoling and alleviate their experiences of suffering.
Fraulob and Davies [31] UK	Qualitative responses in the English Cancer Patient Experience Survey (CPES)	To explore experiences of general practice care and support	National Health Service care	84 comments analysed	Slowness in referral for investigation, delay in receiving scan results, lack of supportive response from the GPs, lack of follow-up care overall suboptimal coordination in care.
Gately et al. [32] Australia	Semi-structured interviews that were audiotaped and transcribed verbatim. Thematic analysis used	To explore the lived experience of long-term survivors of glioblastoma	Tertiary centre	6 long-term survivors and 4 caregivers	Long-term survivors of glioblastoma experience disconnection from themselves from the time of diagnosis into survivorship, which evolves over time. Clinicians need to consider the emotional impact and adopt a holistic approach, including the early introduction of psychosocial support to patients and their caregivers and the role of language in clinical encounters.
Halkett et al. [33] Australia	Qualitative interviews using grounded theory and Maslow’s hierarchy of needs	Explore the experience of patients with PMBT	Medical oncology department of a tertiary referral centre for neurological cancers	19 patients	Patients with brain tumours may have unique needs. Health professionals need to clarify patients’ information and support needs and be aware that this change over and within time.
Hazen et al. [34] USA	Individual semi-structured interviews that were audiotaped	Explore Information and symptom management	Medical oncology department of a tertiary referral centre	7 patients and 6 caregivers	Uncertain about the future, could not get a clear prognosis, did not know how their disease would progress or how to make plans. Concerns such as weight gain, seizures, visual and speech deficits, and inability to drive are more unique to patients with brain cancer. Carers played an essential role in assisting patients with decision making, managing their health, and assisting them physically.
Heckel et al. [35] USA	Individual semi-structured interviews that were audiotaped	Explore mobile health and patient-facing technologies	Local brain tumour support group and radiation clinic	7 patients and 6 caregivers	Participants highly willing to use technologies to capture and manage information, provided they were designed according to the needs, interests, and abilities of these users. Participants felt that such tools could benefit patient care activities and help to address information challenges for both current and future patients and caregivers.
Hricik et al. [36] USA	Individual semi-structured interviews that were audiotaped	To compare experiences, perceived burdens, and needs during home care of informal caregivers	Medical oncology department of a tertiary referral centre	10 informal caregivers affected by brain cancer	Need for improved informational support among caregivers and better support to cope with the physical and psychological changes of the patient.
Langbecker et al. [37] USA	Individual semi-structured using qualitative description	To explore the transition into the caregiver role and how their perceptions of this transition change over time	Neurosurgery and neuro-oncology clinics of a regional medical centre	10 informal caregivers	Caregivers described difficulties stemming from the patient’s tumour-related dysfunction and changes in their familial, occupational, and social roles. Support from family and friends was vital to caregivers’ emotional health, but shock and fear were evident. Difficulty in communicating with healthcare providers.
McConigley et al. [38] Australia	Qualitative interviews using grounded theory	To explore the experiences of adults with primary brain tumours who have unmet needs	Multidisciplinary rehabilitation, community, and psychosocial services	21 informal caregivers	Rapid change and need for timely informational support.
Molassiotis et al. [7] UK	Longitudinal Interviews over 4 time points analysed using content analysis	To explore symptom experience	Specialist oncology centre	9 patients	Key issues for support included ongoing fatigue, memory loss, and inability to drive. Fatalistic views about the outcomes of their disease. Adjustments to their lives to accommodate their functional limitations (including home alterations, introducing regular exercise to their lives, and using complementary therapies). Several participants angry and dissatisfied with health care professionals.
Nixon and Narayanasamy [39] UK	Qualitative study using critical incident technique	To explore spiritual needs	Specialist oncology centre	21 patients	Some patients with brain tumours do report spiritual needs during their hospital stay and some of these needs are not met by nurses.
Ownsworth et al. [40] UK	Critical Incident Technique questionnaire and analysed thematic content analysis	To gain insights into the spiritual needs of neuro-oncology patients	Neurosurgical unit of the local NHS trust	21 patients	Some but not all participants would like support from nurses in the neurosurgical setting with meeting spiritual needs. Identified needs related to family and emotional support, need for connection loneliness/state of despair, religious needs, reassurance meaning and purpose, plans for future/re-establishing a sense of normality.
Philip et al. [41] Australia	Phenomenological approach using in-depth interviews	To explore family caregivers’ experiences of support and relationship changes	Specialist oncology centre	11 family caregivers	Overall, the findings highlight that there is considerable variability in caregivers’ experiences and expectations of support and the impact of brain tumour on relationships.
Piil et al. [8] Denmark	Longitudinal Interviews over 5 time points and audio recorded	To elucidate patients’ and caregivers’ experiences and needs for rehabilitation	Department of Neurosurgery, University Hospital of Copenhagen	33 patients and 33 caregivers	Five themes 1) “individual strategy for acquiring prognostic information” revealed two different strategies for coping. 2) “shared hope,” was based on a strong sense of solidarity between the patient and the caregiver, 3) “engagement in health promotion activities,” was facilitated by shared hope, as the patients and caregivers jointed forces toward a healthier lifestyle reflecting that being involved and taking responsibility were highly valued. 4) (present for patients alone), “adjustment to symptom limitations,” emerged from experiences of and responses to the symptoms/complications and neurological deterioration resulting in physical/ cognitive and functional decline, daily activity limitations, and role changes. 5) (caregivers alone), “role transition from family member to caregiver,” describes the changing role from being a family member to becoming a caregiver.
Raju and Reddy [42] Australia	Qualitative interviews using grounded theory	To understand patient experience of high-grade glioma at the end of life	Specialist oncology centre	10 patients	Despite the medical treatment and supportive care available, there remains a gap in services addressing complex existential and psychosocial needs that were markedly valued by patients. Unmet needs related to loss of self, impending loss and decline, loneliness, and isolation, focus on the here and now, doping day to day and waiting and uncertainty.
Sterckx et al. [43] Germany	Retrospective thematic analysis of interviews at first consultation	To identify the concerns and burdens presented during initial consultation	Specialised in psycho-oncology	53 patients	Increased awareness of the psychological needs of patients to define treatment strategies.
Tastan et al. [44] Belgium	Qualitative interviews using grounded theory	Identify patient experience and care needs	Specialised in psycho-oncology	17 patients	Aa life-changing diagnosis associated with feelings of shock, loss, uncertainty, anxiety, and disregard. Patients also expressed great inner strength. Primary needs from professional caregivers included information, support, and availability.
Vedelø et al. [45] Turkey	Semi structured interviews analysed using a phenomenological approach	To explore experiences of patients’ relatives during the perioperative period and home care	Neurosurgery department of a military hospital	10 caregivers	The patients’ relatives’ needs for knowledge and the psychosocial situation were neglected. Relatives wanted more knowledge about the surgical procedure, possible complications, patient care and home care.
Vedelø et al. [46] Denmark	Longitudinal interviews and observations	Patient experiences during diagnosis of brain cancer	Hospital	4 patients	Four major themes were identified: information needs, balancing hope and reality while trying to perceive the unknown reality of brain cancer, not knowing what to expect and participants’ perceptions of the relationship with the healthcare providers. The analysis revealed that participants were in risk of having unmet information needs and that contextual factors seemed to cause fragmented care that led to feelings of uncertainty and loss of control.
Wasner et al. [47] Denmark Norway	Longitudinal single case study	Exploring an integrated Brain Cancer Pathway from a patient perspective	Specialist oncology centre	1 patient	Patient experienced being alone, although surrounded by healthcare providers Had to develop strategies to manage the responsibilities given in the pathway. Needs related to information, communication, and support clearly changed overtime.
Whisenant et al. [48] USA	Qualitative interviews using story theory	Explore the experiences of informal caregivers	Specialist cancer hospital	20 patients and 20 caregivers	Themes related to commitment, expectation management, role negotiation, self-care, new insight, and role support were identified in this caregiver population.

**Table 2:**
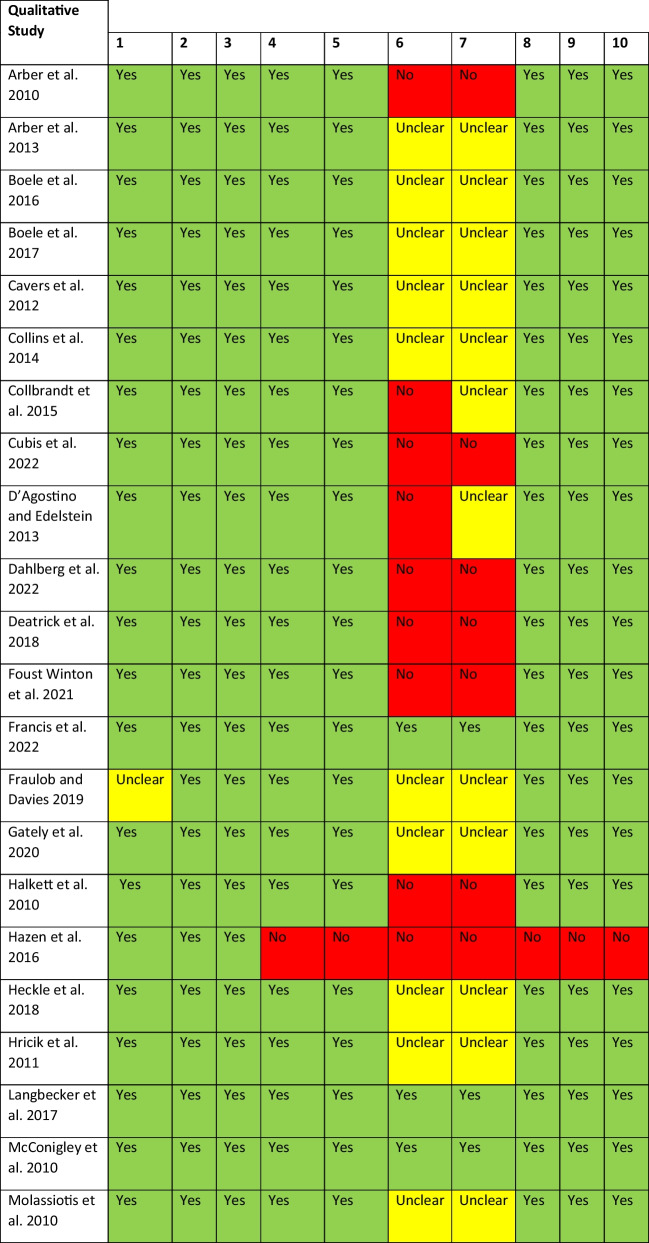

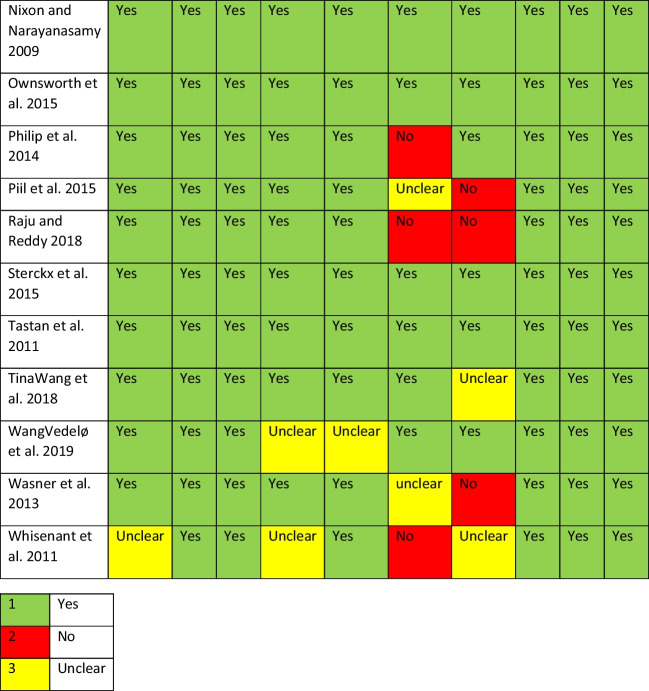
Quality appraisal of primary studies

Item number check list key*: (1) Is there congruity between the stated philosophical perspective and the research methodology? (2) Is there congruity between the research methodology and the research question or objectives? (3) Is there congruity between the research methodology and the methods used to collect data? (4) Is there congruity between the research methodology and the representation and analysis of data? (5) Is there congruity between the research methodology and the interpretation of results? (6) Is there a statement locating the researcher culturally or theoretically? (7) Is the influence of the researcher on the research, and vice-versa, addressed? (8) Are participants, and their voices, adequately represented? (9) Is the research ethical according to current criteria for recent studies, and is there evidence of ethical approval by an appropriate body? (10) Do the conclusions drawn in the research report flow from the analysis, or interpretation, of the data?

There was a total of 220 individual findings included in this review (see Supplementary Table 2), which were synthesised into two findings: (1) caregivers and patients perceived supports which would have been helpful, and (2) caregiver and patients experiences of unmet supportive care needs; see Table 3.

**Table 3:** Synthesized findings

Findings	Categories	Synthesized Finding
F3, F4, F5, F6, F8, F9, F10, F23, F26, F31, F32, F33, F39, F42, F45, F46, F94, F100, F114, F116, F146, F148, F164, F165, F166, F181, F201, F215, F217, F218, F220	Tailored information Practical support Complementary therapies Social network Caring healthcare professionals	**Perception of what support would have been helpful** *Caregivers* Informal caregivers needed timely access to information and practical support from both their healthcare team and wider social networks. Receiving practical support and targeted information to support self-management for both their loved one with brain cancer and themselves was viewed as essential. It was imperative that healthcare professionals provided family-centred care not only for the patient diagnosed with brain cancer but also for the caregiver as well. *Patient* Patients diagnosed with brain cancer reported perceived benefit in remote needs–based monitoring healthcare systems with their healthcare professionals. Having the right documented information in their next steps in care and treatment was important, as well as targeted documented probes to ask their care team. Many patients were afraid of the word “palliative care” which compounded their existential distress, but they would have valued an earlier referral as for many this was an inevitable part of the disease course. Patients relied completely on their caregiver and social network for daily living.
F24, F59, F61, F62, F80, F84, F85, F108, F111, F130, F132, F134, F135, F140, F153, F160, F167, F169, F173, F186, F188, F189, F195, F196	Home-based digital monitoring Documented specific probes Early access to palliative care Caregiver Social network	
F1, F19, F20, F21, F22, F27, F28, F29, F30, F70, F113, F182, F200, F7, F12, F64, F69, F71, F72, F73, F76, F77, F79, F115, F145, F146, F2, F67, F178, F179, F180, F199, F206, F207, F210, F218, F14, F86, F92, F99, F117, F120, F219, F147, F11, F13, F37, F113, F44, F50, F51, F52, F88, F119, F125, F175, F176, F177, F203, F205, F211, F209, F213, F214, F215, F66, F68, F74, F75, F78, F87, F89, F90, F91, F93, F98, F112, F114, F118, F124, F126, F144, F149, F168, F197, F198, F202, F204, F213, F211, F212	Lack informational support Poor care coordination Lack of social support Caregiver role	**Actual experiences of unmet supportive care needs** *Caregivers* Caregivers expressed that they experienced a lack of informational support, advice and care coordination with problems with continuity of care. Caregivers reported that they were poorly, if at all, prepared for the enormity of their caregiver role. Not only did caregivers experience a lack of supportive care within the healthcare system but experienced diminished social support from family and friends overtime. *Patients* Patients articulated a lack of tailored information and time provided to them during consultations with their healthcare professionals. Patients express frustrations with a lack of general support from their General Practitioners and sub-optimal communication between primary and secondary care providers. Patients expressed unmet physical, psychological and social needs with profound existential distress with little support available to them.
F16, F17, F18, F25, F55, F87, F101, F102, F107, F109, F110, F123, F133, F136, F157, F159, F169, F174, F183, F185, F187, F191, F192, F193, F34, F35, F43, F54, F56, F82, F103, F104, F105, F128, F150, F163, F172, F36, F49, F53, F95, F96, F97, F122, F127, F167, F190, F40, F41, F47, F80, F81, F128, F129, F141, F57, F58, F60, F61, F63, F65, F106, F131, F137, F138, F139, F142, F143, F158, F161, F162, F168, F170, F171, F184, F154, F155, F83, F121, F151, F152, F156, F194	Lack of information Patient–clinician relationship Physical, psychological, social unmet needs Existential distress	

### Perceived supports which would have been helpful

#### Informal caregivers

It was clear across many of the included studies that having access to the right information at the right time was important. For caregivers, information access was essential at time of diagnosis, hospital discharge, post treatment and into the disease trajectory [9, 35, 40, 48]. Caregivers would have found it helpful to have a checklist from their healthcare professionals to help them understand what was going to happen next for the person with malignant brain cancer [9, 21, 40, 47], and some found it helpful to audio record their conversation during appointments [48]. Many caregivers lacked upfront information at the time of diagnosis from their clinical teams and consequently found their own source of online information. Helpful sources included the International Brain Tumour Alliance, Cancer Research UK and online support groups [9]. Information about the option of clinical staff remote monitoring digital needs assessment and virtual communication with the clinical team was perceived as potentially useful [21]. Another source of support [47] was finding someone to talk to and getting practical help and guidance from other caregivers, for example, advice on financial benefits, information and recommendations from other caregivers on relaxation days and having the space to express their own feelings of frustration without the sense of guilt [19, 21, 28, 30].

“The Marie Curie Day Nurse ... she was just like, it is like Mary Poppins arriving (laughs). You know she’s a very, very good person.” Caregiver (page 54) [19]

It was noted by caregivers that having access to cancer well-being centres for both their loved ones diagnosed with brain cancer and themselves was important to access complimentary therapies, such as relaxation, Reiki and massages and also as they promoted and enabled social support through connection with other families in a similar situation [19]. Informal support outside the clinical team was of central importance, for example having a social network of family, friends and neighbours to help with groceries, household chores, financial assistance, child-minding, cooking dinner and socialising [21, 30, 40, 44], but this support often dissolved over time when disease trajectory worsened [22, 35]. For those caregivers from a faith-based community, having the opportunity to meet other church members was valued for social, emotional and spiritual support [22, 48]. For some, it was important to experience personal growth in gaining new perspectives, skills and knowledge in caring giving of their loved one [48].

“The good thing about it is I think we have learned to appreciate each day.” Caregiver (page 5) [48]

Having healthcare professionals who were competent and could communicate with empathy, understanding and compassion to their own needs and their loved one was essential [22, 48], as well as having the opportunity to have questions answered [35]. It was also important that healthcare professionals supported self-management for both the patient living with brain cancer but also to support coping mechanisms for the caregiver in health-promoting activities, through providing family-centred cancer care [8].

#### Patients

Patients living with brain cancer articulated that they perceived benefit in remote symptom monitoring and needs-based assessments through digital health platforms which they could complete at home and connect with their hospital care team [21]. Patients perceived that this model of care would empower them with increased knowledge about their condition and to help them self-care with instant advice and better access to their care team [21].

“I believe yes, that … that would, of course, be very convenient if you could just arrange it through the computer. […]. Then you don’t have to be there at half past ten. […] So yes, that might be even more appealing. Also because you then could do this more often. Without constantly going to and fro.” Patient (Male, page 3019) [21]

Patients wanted specific known question probes to ask and document information for symptom management and structured check-ups with their healthcare professionals to ensure timely identification of cancer recurrence or progression [8, 26, 42, 45, 46]. While for many patients the word ‘palliative care’ was frightening, patients knew that they would require palliative care services as an inevitable part of the disease course [41]. Patients expressed that earlier access to palliative care services would have been helpful in coping with symptom management and importantly to ensure that they also had an advance care plan in place [41]. Therefore, a positive relationship between the patient and the healthcare professional team was imperative [45].

“... it was a good conversation. He is a pleasant doctor; he was nice and made me calm.” Patient (Male, page 344) [45]

Many patients experienced significant emotional [39] and existential distress and expressed that having sources of support and reassurance [39] from family, friends and healthcare professionals was crucial [7, 22, 25, 43] including peer support from other people diagnosed with brain cancer [34]. It was clear that patients affected by brain cancer relied completely on their caregiver who was often their most important support [33, 39], and patients counted on them to advocate on their behalf when they could no longer communicate their needs [41]. Some patients found comfort from a spiritual response to existential distress such as through faith in God or an afterlife as a comfort, particularly when the disease progressed [7, 22].

“I believe there’s life after death … so that way I’m not frightened of dying … It calms me down. I know whatever happens, when it happens, will be the Lord’s decision, not mine.” Patient (Male, page 378) [22]

### Experiences of unmet supportive care needs

#### Informal caregivers

Many caregivers spoke about a lack of informational support, advice and care coordination from healthcare professionals [19, 23, 24, 40, 44, 47] with a lack of knowledge about how to deal with symptoms and the disease sequelae over time [20, 21, 23, 35, 44]. There was a notable lack of continuity of care in specialists (doctors, nurses and district nurses), and caregivers expressed that this was imperative particularly when their loved one experienced confusion and cognitive decline [23].

“We were just being handballed around. No one was going to take responsibility and tell us what we had to do.” (Caregiver, page 5) [23]

Specifically, caregivers wanted assistance from healthcare professionals to prepare them for their caregiver role including enabling them to enlist support and plan key transitionary stages of diagnosis, discharge, during treatment and at tumour progression [23, 24, 40]. Caregivers reported that they needed help in accessing early palliative care services and trying to encourage their loved one to accept such services [19, 22]. It was also important that caregivers were given information about how to manage medications and side-effects of treatment safely [19, 44]. Many caregivers also reported that they were ill prepared for coping with personality changes, impulsive and aggressive behaviour at home unsupervised from healthcare professionals [23, 44, 47, 48].

Unfortunately, not only did caregivers experience a lack of support from their healthcare professional team, but they also experienced reduced support within their families and social networks over time [19, 27, 28, 30, 35]. However, some families reported strengthened connections [40].

“In hindsight my sister-in-law once said to me, I have now been in there [with the patient], we went home, I was all run down. And, she says, I now can understand you when you say, you are run down. I could not have stayed in there over night, I could not.” (Caregiver, p197) [35]

Caregivers reported a high level of stress caring for their loved one [22, 38, 44], and coping with the progressive personality changes was most disturbing and distressing [26, 44] resulting in renegotiating relationships [47]. Some caregivers developed anxiety and depression with a lack of timely referrals for needed support in the healthcare system [22, 47]. Caregivers expressed significant burden and psychological distress because of the involuntary caregiver role forced upon them. Many expressed that their role was 24-7 hours, and they provided constant availability to tend to their loved ones’ needs, which compounded a sense of isolation [8, 23, 27, 30, 35, 36, 38, 47, 48]. Their caregiver role encompassed navigating the healthcare system, making treatment decisions, driving patients to appointments, being an advocate, administering medications, managing seizures, providing daily living assistance with meals, bathing, toileting, cleaning, looking after children and being the sole income provider in the family [8, 23, 24, 27, 28, 30, 35, 36, 38, 40, 47].

“From that moment [of diagnosis], everything was different … As the seizures progressed, she started losing more of her abilities – she lost the ability to eat, to drink, to stand, to walk. Her sanitary needs were done by me, everything was done by me.” (Caregiver, page 5) [23]

#### Patients

Patients reported that there was limited time with their clinicians which impacted on the quality of the information provided to them to meet their needs [7, 20, 33, 41–43, 45]. Patients expressed difficulties in navigating the healthcare system [46] and understanding information because of fatigue, language and speech, memory or visual difficulties [7, 21, 33, 34, 37] and having a supportive empathetic clinician was crucial [7, 22, 25, 33].

Patients also expressed a lack of care and support from their General Practitioners to gain help in managing side effects and seizures in the home environment [31] and articulated that there needed to be improved communication between primary and secondary care providers [31, 45]. Many patients experienced anxiety and depression but did not get the needed support from their healthcare professional team [22, 32, 39, 41, 43, 46]. However, for some other patients, they did not want to access supportive care services [37]. It was common among patients affected by brain cancer to experience significant existential distress [7, 22, 33, 42, 45], and for some, this brought them closer to their religion [39]. Over time, patients were able to adjust to death and dying and accepted this as part of the disease course [22], but it was important that they had a support person to talk to [39] because there was limited acknowledgement of their existential distress from their care team [41].

“I feel sad… sometimes, I get fear whenever I think about my death …” (Patient, page 8) [42]

Patients affected by brain cancer reported a significant and distressing physical burden of the disease, from initial physical problems at diagnosis to a rapid downward and debilitating trajectory with a lack of supported self-management [22, 41]. Over time, patients reported an increased frequency and severity of symptoms which included pain [29], fatigue [7], nausea, communication, mobility, strength, understanding their behaviour and physical appearance [22]. Changes to symptoms also led to an increase in dependence on others, which results in a perceived disconnection from the past-self experienced by the person with PMBT [8, 32, 41]. For many patients, as the physical illness progressed, the sense of social isolation deepened [39, 41] as it was difficult to continue work or engage in other social activities compounded by their inability to drive [7, 22, 25, 26, 38].

“Cognitively um like … the other day I was already over at the coffee shop with another friend and Suzie walks in with hands on hips like ‘Angie! Did you forget we’re meeting for coffee?” (Patient, page 10) [25]

## Discussion

This qualitative systematic review set out to understand what supports would have been helpful to people affected by brain cancer and their informal caregivers, and to identify experiences of unmet supportive care needs in existing cancer services, in their own words. Importantly, this research critically synthesised supportive care perspectives from both the patient and their informal caregiver. Both groups reported similar issues with the current provision of brain cancer care. However, what is apparent is that current cancer services are provided solely for patients, with little or no consideration to the support needs of the informal caregiver, and this finding is not dissimilar to other caregivers affected by cancer [49]. What is clear however is that the enormity of the informal caregiver’s role in the context of brain cancer was evident necessitating timely support from the healthcare professional team. All informal caregivers represented in this review reported that they lacked the support, information and preparation to take on and adapt to this role. This finding highlights the need for increased caregiver support to alleviate distress and suffering among caregivers and can be achieved by providing family-based cancer care. This is important for patients as well as caregivers as research has shown that caregivers’ distress can have an impact on patients’ distress, long-term adjustment and anxiety [50].

Key opportunities for future interventions to address unmet needs of both patients and their informal caregiver includes (1) better care coordination to enable tailored and targeted informational support; (2) implementation of holistic needs assessments, for both the patients and their caregivers [11]; (3) better community support provision, anticipatory proactive care rather than reactive, and (4) improved opportunities for emotional care with early streamlined integration for palliative care services. Interventions and clinical service re-design must target the shortcomings in existing services to address the psychological, communication, information and assistance to mobile and re-mobilise social support networks in the community for families affected by brain cancer identified in this review. There was a notable lack of insight provided into preferences for multidisciplinary (MDT) models of supportive care, and this observation is in keeping with previous research, which identified that rehabilitative services are not provided for people diagnosed with brain cancer [51]. It would be highly beneficial to conduct needs-based-holistic assessments (for both patients and informal caregivers) and coordinate care, which would involve medical clinicians, brain cancer specialist nurses, nurse practitioners, occupational therapists, physiotherapists, exercise physiologists, psychologists, social workers, speech pathologists, dietitians, GPs and community nurses, given the profound negative sequelae of brain cancer. Arguably, this clinical group has the highest need to access MDT services and models of care and should be urgently prioritised. A further essential consideration is access to early palliative care services in both acute and community settings to optimise hope [52], normalising the idea of dying [53] to provide the needed reassurance [54].

This qualitative systematic review has highlighted the complexity of dealing with brain cancer, from both the patients and caregivers’ perspective and underscored what they articulated to be helpful. Both patients and informal caregivers wanted open and honest discussions with empathy and compassion about the disease and practical assistance to manage day-today uncertainty and existential distress. Healthcare professionals should prepare patients and caregivers at the onset about what to expect, while tactfully providing hope, sensitive to individual needs, including optimal communication and family-centred cancer care through crisis management, at times of disease progression. The needs of people diagnosed with brain cancer and their caregivers are unlike other cancer trajectories [11]. Therefore, future interventional research should consider a comprehensive targeted holistic-needs–based assessment, safely mobilising a multidisciplinary model of care to enable proactive and anticipatory care, rather than reactive to continual crisis management.

### Implications for survivors

This review has highlighted the suffering and distress caused by brain cancer and associated treatments. Both patients and their informal caregivers experienced disconnect from themselves in renegotiating roles, and a profound sense of loneliness as the physical deterioration of the disease took hold. Cancer and palliative healthcare teams need to consider the emotional impact of brain cancer and provide a comprehensive assessment of the family’s social network, to ensure that appropriate signposting for community support can be suggested and mobilised (for example, peer support groups, access to cancer well-being centres, signposting to charity organisations and respite services for the informal caregiver). Furthermore, little is known about how patients and informal caregivers coped with brain cancer during the COVID-19 pandemic [55] as strict government lockdowns were enforced, restricted visitor policy’s implemented in hospitals with rapid changes from in-person face-to-face consultation and reviews to telehealth models of care [56, 57]. This current review and a recently published systematic review [55] identified that little is known about experiences of supportive care during the pandemic among people affected by brain cancer, and this should be a focus for future research. Further research is also needed to explore the structure and types of social support for the family affected by brain cancer in the community setting, and how this may moderate or mediate the relationship between stress and coping for both the patient and the informal caregiver. Lastly, there was a lack of discussion across all studies about the intimacy and relationship impacts aspects of brain cancer and its side-effects, from both patients and informal caregivers. The reasons for this are unknown; it might be due to the profound and pervasive impact and instant onset of symptoms at diagnosis, and this was not a priority given the disease burden and poor prognosis.

### Limitations

Due to the inclusion criteria, only studies published in the English language were included, and therefore by omission, the findings presented here may not be transferable to other non-English speaking communities. However, this review followed a rigorous and transparent process throughout.

## Conclusion

The findings from this systematic review have provided valuable insights from both patients and the informal caregivers’ perspective, into what supports are helpful, and where future targeted interventions are needed to address unmet supportive care needs. This review has extended knowledge and understanding and provided future directions for clinical practice and research. There is an urgent need to provide family-based cancer care to address the needs of both the patients and their informal caregiver. Service re-design is needed (1) to improve care coordination with individualised informational support, (2) for implementation of holistic needs assessments for both the patients and their caregivers, (3) to better community support provision and (4) for improved opportunities for emotional care with early referral for palliative care services.

## Supplementary information

ESM 1

ESM 2

## Data Availability

Not applicable.
