# Supplementary material for: What are the experiences of supportive care in people affected by brain cancer and their informal caregivers: A qualitative systematic review
Source: J Cancer Surviv. 2023 May 31;18(5):1608–29. doi: 10.1007/s11764-023-01401-5 (PMC10229398; doi:10.1007/s11764-023-01401-5)
Supplement: Supplementary file 1 — ESM 1 [file 11764_2023_1401_MOESM1_ESM.docx]

**Supplementary Table 1. Database searches.**

| Database: APA PsycINFO | | | |
| --- | --- | --- | --- |
| Date of search: April 2022 | | | |
| Symbols used in this document: | | | |
| TI:AB – Title and abstract field of a record | | | |
| N6 – finds words only when they are no more than 6 words apart | | | |
| DE = Psychological Index Term | | | |
| “ ” finds a phrase | | | |
| Asterisk (*) – finds various endings of a word stem | | | |
| **Search #** | **Concept/Explanation** | **Search Terms/Strategy** | **# of Results** |
| #1 | Brain cancer: Keywords | TI:AB “brain cancer*” OR “brain tumor*” OR (brain N6 glioma*) | 2,938 |
| #2 | Brain cancer: MeSH | DE "Brain Neoplasms" | 4,753 |
| #3 |  | #1 OR #2 | 5,971 |
| #4 | Needs and support: Keywords | TI:AB (physical OR psychological OR psychosocial OR social OR emotion* OR “interperson*” OR family OR caregiver* OR partner OR spous* OR intima* OR practical OR financial OR employment OR “daily-living” OR “housekeeping” OR nutrition* OR diet* OR exercise OR spiritual OR existential OR “end-of-life” OR bereavement OR “terminal care” OR hospice OR “palliative care” OR decision* OR cognitive OR “patient-clinician” OR “health care” OR sadness OR fear OR pain OR isolat* OR lonel* OR information* OR transition*) N6 (need* OR support*) | 266,480 |
| #5 | Needs and support: MeSH | DE "Social Support" OR DE "Supportive Psychotherapy" OR DE "Social Networks" OR DE "Caregiving" OR DE "Psychological Needs" OR DE "Health Service Needs" | 82,071 |
| #6 |  | #4 OR #5 | 302,368 |
| #7 |  | #3 AND #6 | 240 |
|  | Data cut-off January 2010-present | Limiter applied | 148 |

| Database: Cochrane Library (Central Register of Controlled Trials and Database of Systematic Reviews) | | | |
| --- | --- | --- | --- |
| Date of search: April 2022 | | | |
| Symbols used in this document: | | | |
| NEAR – finds words only when they are no more than 6 words apart | | | |
| MH = Main Heading or “MeSH Heading” | | | |
| + = Explodes the “MeSH Heading” | | | |
| “ ” finds a phrase | | | |
| Asterisk (*) – finds various endings of a word stem | | | |
| **Search #** | **Concept/Explanation** | **Search Terms/Strategy** | **# of Results** |
| #1 | Brain cancer: Keywords | “brain cancer*” OR “brain tumor*” OR (brain NEAR glioma*) | 1,805 |
| #2 | Brain cancer: MeSH | (MH "Brain Neoplasms+") | 1,925 |
| #3 |  | #1 OR #2 | 3,248 |
| #4 | Needs and support: Keywords | (physical OR psychological OR psychosocial OR social OR emotion* OR “interperson*” OR family OR caregiver* OR partner OR spous* OR intima* OR practical OR financial OR employment OR “daily-living” OR “housekeeping” OR nutrition* OR diet* OR exercise OR spiritual OR existential OR “end-of-life” OR bereavement OR “terminal care” OR hospice OR “palliative care” OR decision* OR cognitive OR “patient-clinician” OR “health care” OR sadness OR fear OR pain OR isolat* OR lonel* OR information* OR transition*) NEAR (need* OR support*) | 50,074 |
| #5 | Needs and support: MeSH | (MH "Health Services Needs and Demand+") | 444 |
| #6 | Needs and support: MeSH | (MH "Needs Assessment") | 352 |
| #7 |  | #4 OR #5 OR #6 | 50,479 |
| #8 |  | #3 AND #7 | 133 |
|  | Exclude Cochrane protocols |  | 128 |
|  | Data cut-off January 2010-present | Limiter applied | 111 |
| Note - 111 results include 44 systematic reviews and 67 trials. | | | |

| Database: Cumulative Index to Nursing and Allied Health Literature (CINAHL) | | | |
| --- | --- | --- | --- |
| Date of search: April 2022 | | | |
| Symbols used in this document: | | | |
| TI:AB – Title and abstract field of a record | | | |
| N6 – finds words only when they are no more than 6 words apart | | | |
| MH = Main Heading or “CINAHL Heading” | | | |
| + = Explodes the “CINAHL Heading” | | | |
| “ ” finds a phrase | | | |
| Asterisk (*) – finds various endings of a word stem | | | |
| **Search #** | **Concept/Explanation** | **Search Terms/Strategy** | **# of Results** |
| #1 | Brain cancer: Keywords | TI:AB “brain cancer*” OR “brain tumor*” OR (brain N6 glioma*) | 4,961 |
| #2 | Brain cancer: MeSH | (MH "Brain Neoplasms+") | 15,238 |
| #3 |  | #1 OR #2 | 17,113 |
| #4 | Needs and support: Keywords | TI:AB (physical OR psychological OR psychosocial OR social OR emotion* OR “interperson*” OR family OR caregiver* OR partner OR spous* OR intima* OR practical OR financial OR employment OR “daily-living” OR “housekeeping” OR nutrition* OR diet* OR exercise OR spiritual OR existential OR “end-of-life” OR bereavement OR “terminal care” OR hospice OR “palliative care” OR decision* OR cognitive OR “patient-clinician” OR “health care” OR sadness OR fear OR pain OR isolat* OR lonel* OR information* OR transition*) N6 (need* OR support*) | 208,611 |
| #5 | Needs and support: MeSH | (MH "Health Services Needs and Demand+") OR (MH "Needs Assessment") | 42,562 |
| #6 |  | #4 OR #5 | 241,606 |
| #7 |  | #3 AND #6 | 358 |
|  | Data cut-off January 2010-present | Limiter applied | 250 |

| Database: MEDLINE | | | |
| --- | --- | --- | --- |
| Date of search: April 2022 | | | |
| Symbols used in this document: | | | |
| TI:AB – Title and abstract field of a record | | | |
| N6 – finds words only when they are no more than 6 words apart | | | |
| MH = Main Heading or “MeSH Heading” | | | |
| + = Explodes the “MeSH Heading” | | | |
| “ ” finds a phrase | | | |
| Asterisk (*) – finds various endings of a word stem | | | |
| **Search #** | **Concept/Explanation** | **Search Terms/Strategy** | **# of Results** |
| #1 | Brain cancer: Keywords | TI:AB “brain cancer*” OR “brain tumor*” OR (brain N6 glioma*) | 43,271 |
| #2 | Brain cancer: MeSH | (MH "Brain Neoplasms+") | 151,590 |
| #3 |  | #1 OR #2 | 165,944 |
| #4 | Needs and support: Keywords | TI:AB (physical OR psychological OR psychosocial OR social OR emotion* OR “interperson*” OR family OR caregiver* OR partner OR spous* OR intima* OR practical OR financial OR employment OR “daily-living” OR “housekeeping” OR nutrition* OR diet* OR exercise OR spiritual OR existential OR “end-of-life” OR bereavement OR “terminal care” OR hospice OR “palliative care” OR decision* OR cognitive OR “patient-clinician” OR “health care” OR sadness OR fear OR pain OR isolat* OR lonel* OR information* OR transition*) N6 (need* OR support*) | 368,124 |
| #5 | Needs and support: MeSH | (MH "Health Services Needs and Demand+") OR (MH "Needs Assessment") | 87,997 |
| #6 |  | #4 OR #5 | 439,735 |
| #7 |  | #3 AND #6 | 945 |
|  | Data cut-off January 2010-present | Limiter applied | 629 |

| Database: ProQuest (Health & Medical collection and Nursing & Allied Health database) | | | |
| --- | --- | --- | --- |
| Date of search: April 2022 | | | |
| Symbols used in this document: | | | |
| TI:AB – Title and abstract field of a record | | | |
| NEAR/6 – finds words only when they are no more than 6 words apart | | | |
| MH = Main Heading or “MeSH Heading” | | | |
| + = Explodes the “MeSH Heading” | | | |
| “ ” finds a phrase | | | |
| Asterisk (*) – finds various endings of a word stem | | | |
| **Search #** | **Concept/Explanation** | **Search Terms/Strategy** | **# of Results** |
| #1 | Brain cancer: Keywords | TI:AB “brain cancer*” OR “brain tumor*” OR (brain NEAR/6 glioma*) | 31,874 |
| #2 | Brain cancer: MeSH | (MH "Brain Neoplasms+") | 8,826 |
| #3 |  | #1 OR #2 | 38,180 |
| #4 | Needs and support: Keywords | TI:AB (physical OR psychological OR psychosocial OR social OR emotion* OR “interperson*” OR family OR caregiver* OR partner OR spous* OR intima* OR practical OR financial OR employment OR “daily-living” OR “housekeeping” OR nutrition* OR diet* OR exercise OR spiritual OR existential OR “end-of-life” OR bereavement OR “terminal care” OR hospice OR “palliative care” OR decision* OR cognitive OR “patient-clinician” OR “health care” OR sadness OR fear OR pain OR isolat* OR lonel* OR information* OR transition*) NEAR/6 (need* OR support*) | 285,648 |
| #5 | Needs and support: MeSH | (MH "Health Services Needs and Demand+") OR (MH "Needs Assessment") | 12,743 |
| #6 |  | #4 OR #5 | 295,731 |
| #7 |  | #3 AND #6 | 376 |
|  | Data cut-off January 2010-present | Limiter applied | 260 |

| Database: Scopus | | | |
| --- | --- | --- | --- |
| Date of search: April 2022 | | | |
| Symbols used in this document: | | | |
| TI:AB – Title and abstract field of a record | | | |
| W/6 – finds words only when they are no more than 6 words apart | | | |
| “ ” finds a phrase | | | |
| Asterisk (*) – finds various endings of a word stem | | | |
| **Search #** | **Concept/Explanation** | **Search Terms/Strategy** | **# of Results** |
| #1 | Brain cancer: Keywords | TI:AB “brain cancer*” OR “brain tumor*” OR (brain W/6 glioma*) | 60,038 |
| #2 | Needs and support: Keywords | TI:AB (physical OR psychological OR psychosocial OR social OR emotion* OR “interperson*” OR family OR caregiver* OR partner OR spous* OR intima* OR practical OR financial OR employment OR “daily-living” OR “housekeeping” OR nutrition* OR diet* OR exercise OR spiritual OR existential OR “end-of-life” OR bereavement OR “terminal care” OR hospice OR “palliative care” OR decision* OR cognitive OR “patient-clinician” OR “health care” OR sadness OR fear OR pain OR isolat* OR lonel* OR information* OR transition*) W/6 (need* OR support*) | 896,854 |
| #3 |  | #1 AND #2 | 801 |
|  | Data cut-off January 2010-present | Limiter applied | 542 |
