# Supplementary material for: What are the experiences of supportive care in people affected by brain cancer and their informal caregivers: A qualitative systematic review
Source: J Cancer Surviv. 2023 May 31;18(5):1608–29. doi: 10.1007/s11764-023-01401-5 (PMC10229398; doi:10.1007/s11764-023-01401-5)
Supplement: Supplementary file 2 — ESM 2 [file 11764_2023_1401_MOESM2_ESM.docx]

**Study Findings and Illustrations**

| **Authors: Arber et al. 2010** | | | | |  |  |  |
| --- | --- | --- | --- | --- | --- | --- | --- |
| **Participants, Clinical and Demographic Characteristics** | **Findings** | **Illustrations (Page number)** | **Evidence** | | | | |
|  |  |  | **Unequivocal** | **Credible** | | **Unsupported** | **Finding number** |
| N=22 caregivers.  Twelve female partners, five male partners, two daughters, one son, one mother and one father who were caring for family members with a primary malignant brain cancer (PMBT).  Most carers in the sample were under 60 years of age—five carers were in their 60s, six were in their 50s, four were in their 40s, six were in their 30s and one was in her 20s. | Information at discharge | **Caregiver** We weren’t given any information, nothing. We weren’t given any help until last year when I kicked up a fuss big time. (Wife 15) Pg 330  **Caregiver** In the beginning I had no advice on what happens to people with brain tumours. The health professionals didn’t guide me where to go. I think it was all there but I didn’t take it, I don’t know. (Wife 1) Pg 330  **Caregiver** The day they finally said he could leave he scrambled in the car, he just wanted to go, but you’re feeling very insecure if anything goes wrong. You’ve not got a checklist, I wasn’t given anything. Just all the Sister said, it was a Sunday night, she said ‘Well give us a ring if you’ve got any problems.’ But I said: ‘Have you ever tried to ring this hospital? I have and you don’t get through.’ ‘Oh,’ [she said] ‘I think you will.’ I said: ‘No, you’re telling me what you want me to hear, not what happens …’ If this had been his first operation I would have been terrified because you get him home, you don’t know. Then his wound got infected. I’ve seen infection, I said ‘This is infected, we’ve got to do something’, but our local hospital doesn’t want to know and he won’t go to them anymore because he’s had such a bad experience. (Father 5)  Pg 331  **Caregiver** I did have a call from one lady, a physiotherapist from the district general hospital, who actually, I don’t think lived on the same planet as the rest of us. She basically said to me ‘We’re going to be discharging your husband’, and I said: ‘Oh okay, so what are you going to put in place to help us?’ because I work and I was working full time at that stage. She said: ‘Well, can’t you give up your work?’ And I said: ‘I’m sorry but no, I can’t give up work’, and she said: ‘Well if you give up work they’ll pay your mortgage for you.’ I said: ‘I don’t think it works like that’ and she gave me all this wrong information that I knew was a load of old rubbish, and in the end she said: ‘Well there’s nothing much we can offer you.’ And I thought, well great, you know. (Wife 18) Pg 331 | X |  | |  | F1 |
|  | Managing medications | **Caregiver** Well he’s recovering from being taken off the [cortico] steroids too quickly. I knew it was happening, we’d already been [given an] authorized dose. I didn’t quite understand the working of them. I guess I need to know a bit more. (Wife 12) Pg 331  **Caregiver** I could have done with a two-day seminar on [cortico] steroids and the effects of [cortico]steroids. Nobody tells you … He didn’t sleep on steroids. They don’t tell you that. (Wife 1) Pg 331  **Caregiver** So I would take him to the hospital and he’d go in and I’d say: ‘Do you want me to come in’ –‘no’ – ‘ok alright,’ I had to respect that. And then he’d come out and we’d come back and would be querying straight away. ‘What do I do with these tablets? What do I do?’ I would phone up and they would say: ‘Oh no, we’re not allowed to discuss this with you because you are not put down as a person.’ So I think once you have established there is a carer, whoever they are—whether they are a parent or a wife—that aspect has got to be sorted. Because you know you are up there usually all day going from one clinic to another and if you’re not given the information, the patient in many cases is not capable of relaying it to you. I came back here the first time with his drugs for the chemo and you had to make it up. I think the dose was something like … There were three tubs to open, take the right amount out and calculate the dosage. In his worse state he couldn’t remember what he’d had for his lunch when he got it. I am sure I’m not the only one to come across that. But this time I’ve gone in and I’ve seen people like the clinical nurse specialist who’ve got it clear. (Father 5)Pg 331 | X |  | |  | F2 |
|  | Accessing information | **Caregiver** There is a really good website. It’s called the International Brain Tumour Alliance. Because his condition is so devastating and everything [and] the prognosis is really bad, it is good to find some websites that give you, actually, hope. And there is some hope [that] there are people who can survive. (Wife 17)  Pg 332  **Caregiver** We’ve got to go back at the end of March and they will review it again. There’s a new drug out that I looked at on the Internet that’s supposed to be quite effective with radiotherapy and I don’t know whether it would be possible for him to have that if he does have to have another course at some stage but we’ll wait and see. (Son 16)  Pg 332  **Caregiver** He was being treated at the [district general hospital centre], and it was only through searching the Internet that I found out about the support group at the specialist hospital. (Wife 19)  Pg 332  **Caregiver** I found it very useful. You could write your story, just a few lines, and then I’ve emailed [it] and you know in fact I printed all my emails to one particular lady the other day. I don’t know what I’m going to do with this but I’ll do something with it all one day. But you can see the shock, my shock, her resignation because she was a month ahead of me, and then you could see my hopeful bit, like at Christmas, the scan was good. (Wife 1)  Pg 332  **Caregiver** Cancer Research UK’s been quite good but again they have warnings up that you might not want to read what’s coming. (Female partner 8)  Pg 332 | X |  | |  | F3 |

| **Authors: Arber et al. 2013** | | | | |  |  |  |
| --- | --- | --- | --- | --- | --- | --- | --- |
| **Participants, Clinical and Demographic Characteristics** | **Findings** | **Illustrations (Page number)** | **Evidence** | | | |  |
|  |  |  | **Unequivocal** | **Credible** | | **Unsupported** | **Finding Number** |
| N=22 caregivers of PMBT individuals  12 female partners, 5 male partners, 2 daughters, 1 son, 1 mother and 1  father.  Most carers (n = 17) were aged under 60 years and 15 were female. | Finding someone to talk to and getting practical help | **Caregiver** This is a book a friend gave me. He’s a carer too. His wife has Alzheimer’s. Peculiar title [Selfish Pig’s Guide to Caring] it’s a book..he goes to a carers meeting to do with Alzheimer’s and he’s out of his depth a bit with his wife.So in fact he bought it for me because we tend to talk to one another because although it is different caring, different outcomes and everything we..other carers understand and when you say certain things, which you can’t say, I find, or don’t feel you can to friends or family who’ve got no knowledge of what it is like to be a carer (Wife 22).  Pg 54 | X |  | |  | F4 |
|  | Someone to help with benefits | **Caregiver** And they got ( ) carers in touch with us, which was Mary Wilson and she’s been fantastic and she has given me all the help that I need. She’s contacted other people for me, she’s explained things, she’s helped us with our benefits, as we weren’t getting loads of stuff and she helped us and she gave us all the information and she’s got me into days like relaxation days. .But before then we had nothing and we were told nothing. We just plodded along coping on our own. (Wife 15)  Pg 54 | X |  | |  | F5 |
|  | Time out from caring | **Caregiver** “The Marie Curie Day Nurse.. she was just like, it is like Mary Poppins arriving (laughs). You know she’s a very, very good person” (Wife 1).  Pg 54 | X |  | |  | F6 |
|  | Importance of having a relationship with the person providing care | **Caregiver** You can get volunteers but it’s a bit like the district nurses, some of them are nice, some of them are clued in, but some of them turn up and, one came on a Saturday morning, I hadn’t seen the woman before, her uniform was covered by a coat. She said she was from Church Road, I didn’t know what that meant, I wasn’t sure if she was a Jehovah’s witness, and it was only when she said’ have you someone in there who is terminal’ that I realised she was actually a nurse, because as I said we had deferred them from weekends (Husband 2)  Pg 54  **Caregiver** She says I’m to use that and it is for somebody to sit with him. I can either use it on a regular basis a couple of hours every Tuesday or whatever it is. Or I can save it up and have a day out or longer time. Well that’s all very well but I don’t know any of these people and I feel very uneasy about just saying yes, a complete stranger can come in. He may not like them... (Wife 22). Pg 55 | X |  | |  | F7 |
|  | So it is all really therapeutic stuff | **Caregiver** The other source of help has been the Apple Tree in Stockley. They are a centre, which support anyone with cancer and they have been absolutely fantastic. He has been going there for a year and a half now. He’s had counselling there. He’s had treatments like Reiki, massages and a couple of days ago he had a session up there where they were making necklaces. So it is all really therapeutic stuff and I know he can go there once a week and feel safe. It is a set time say, two hours and that’s really great for him (Wife 19)  Pg 55  **Caregiver** When he first went there he was frightened because he though’ oh god why am I coming here e it’s cos they think I’m gonna die’. It wasn’t that at all. It’s given him a hell of a lot and me as well. It’s been lovely. He’s made friends there although some of them have already died over the years so he’s had to deal with that himself. But it’s an incredible place (Wife 14).  Pg 55 | X |  | |  | F8 |
|  | Time out of the home socialising | **Caregiver**...the Cancer Centre who are brilliant. Pat goes there once a week on Thursday.She goes for lunch, they’re a lovely bunch of people I’d recommend them to anyone. they have therapies and things, and I can have therapies there if I want them, and I occasionally have a massage when tension brings my shoulders up by my ears.. (Husband 21). Pg 55 | X |  | |  | F9 |
|  | Safe place to express feelings about being a carer | **Caregiver** ‘...I don’t feel guilty when I come away because I feel angry or whatever it is because everybody feels like that so you don’t feel guilty that you are talking about them, or disloyal or anything. You are just talking about the way it is’.  Pg 55 | X |  | |  | F10 |
|  | Difficulties in accepting hospice and palliative care services | **Caregiver** Poppy had almost had an abject fear of cancer and has always kept hospice and people from the palliative care hospice team at,...didn’t want to speak to them. Every time they came near her she broke down in floods of tears (Husband 2)  Pg 55 | X |  | |  | F11 |
|  | Getting the right kind of support | **Caregiver** “So I asked my district nurse and she said ‘well he can always go into respite’ but he’d only just come home, and I said, ‘I want him home with me’, I couldn’t you know, I want him here and I owe him that.” (Wife 1)  Pg 55-56 | X |  | |  | F12 |
|  | Lack of contact with palliative care services | **Caregiver** ‘..she just rings. We haven’t seen her for ages actually. This carer also said she wanted some advice ‘and they can’t give it to you’ (Wife 15).  Pg 56  **Caregiver** I did ask her about sitters and I found that there is an organisation but she said ‘nothing here there’s nothing’, but apparently there is an organisation called Prospero (Wife 1).  Pg 56 | X |  | |  | F13 |
|  | Absence of support within the family | **Caregiver** Some friends have been good some friends haven’t been in touch at all. They can’t handle it, can’t handle the change in Matt. I mean there is a terrific change but he’s still Matt he’s different if I could and Sian [adult daughter] and I used to get quite angry because in a way we had to cope with it. People are very strange (Wife 1). Pg 56  **Caregiver** ‘but I think she is almost jealous of the attention Tom has had since he has been ill’ (Wife 19). Pg 56  ‘His family doesn’t help they haven’t been supportive but that is another story. It is a sore subject really’ (Wife 10). Pg 56 | X |  | |  | F14 |
|  | Support from social care services | **Caregiver** I had a sitter today a person who was arranged by a company called Prospero, which is a support for carers, but I couldn’t have left him with her. Cause she had no idea, she well I couldn’t have left him with her (Wife 1). Pg 56 | X |  | |  | F15 |

| **Authors: Boele et al. 2016** | | | | |  |  |  |
| --- | --- | --- | --- | --- | --- | --- | --- |
| **Participants, Clinical and Demographic Characteristics** | **Findings** | **Illustrations (Page number)** | **Evidence** | | | |  |
|  |  |  | **Unequivocal** | **Credible** | | **Unsupported** | **Findings number** |
| N=15 patients with PMBT  N=15 informal caregivers  Patients n=5 females, n=10 males, age range 28-66 years. Treatments not reported.  Caregivers n=2 males, n=13 females, age range 38-76 years. All spouses. | **Current supportive care provision**  Most patients mentioned to be content with the information provided by the hospital, while many informal caregivers felt this did not suffice or was unclear. Patients indicated that because of time constraints and a varying level of interest from physicians, they did not always feel there was enough attention for the person behind the disease: | **Patient** ‘Yes, it just doesn’t feel so… easy and… familiar, so to speak, and you have the feeling that it all has to be done in a hurry, so, I think, yes... never mind.’ (Female glioblastoma patient (42 years), currently under treatment) (pg 3014) | X |  | |  | F16 |
|  | **Advantages and disadvantages of monitoring** | **Patient** ‘That you… that you had better follow things over time, what is happening to you, how you are doing, yes. Well you know, whether your condition has indeed improved or deteriorated, or your weight... or, those kinds of things. That you are triggered to… to take the necessary action if things are not well.’ (Male grade III oligodendroglioma patient (65 years), progressive disease suspected) Pg 3014 | X |  | |  | F17 |
|  | **Advantages and disadvantages of monitoring**  Downsides of monitoring were also mentioned. Both patients and caregivers said that it could be difficult to master the discipline to monitor regularly and that it can be time consuming. Furthermore, they feared that it would increase awareness of problems they did not know they had and that it could be difficult to face (worsening) symptoms: | **Patient** ‘But I also have to be careful that I do not go and sit there thinking up things, like, what do I find so hard..’ (Male caregiver (76) of a grade III astrocytoma patient with stable disease) Pg 3014 | X |  | |  | F18 |
|  | **Advantages and disadvantages of monitoring**  Participants who experienced no needs considered monitoring to be pointless. However, patients and caregivers did feel that it might be useful for others who do experience needs and are less able to cope with symptoms or distress |  |  |  | | X | NS1 |
|  | **Preferences regarding monitoring**  Patients generated mainly physical symptoms as topics to monitor. Cognitive deficits, changes in personality, mood, and emotional reactions were also mentioned. Caregivers mainly mentioned mental symptoms, such as depressive mood and stress. Moreover, changes in the relationship with the patient and in everyday life, and coping with the patient’s symptoms were frequently mentioned. Many topics mentioned by caregivers were associated with grief and acceptance: | **Caregiver** ‘Also the… amount of time I really spend with my husband, quality time. I have been wondering about… those are also things I wonder about. Like, is this normal what is happening here. What… what do people do when you tell them you only have so and so long to live.’ (Female caregiver (52 years) of a glioblastoma patient with disease progression)  Pg 3014 | X |  | |  | F19 |
|  | **Preferences regarding monitoring**  Both patients and caregivers thought receiving feedback on the results of monitoring was essential. They indicated that with feedback, changes over time become apparent, and it can provide more insight into the problems experienced. Patients would like to know if symptoms are normal considering the circumstances. Several caregivers mentioned that feedback and advice alone could provide solace: | **Caregiver** ‘Yes, in itself I do believe that it... may give some relief since you know there is care available. Oh dear, not that you, that I would immediately use it, but I, again believe, the idea that you, the sheer knowing that it is there... that could be very comforting.’ (Female caregiver (52 years) of a glioblastoma patient who is under treatment) Pg 3015 | X |  | |  | F20 |
|  | **Preferences regarding monitoring**  Subsequent referral to supportive care was considered useful by most patients and caregivers. Informal caregivers believed that referral after monitoring can save time and effort to seek out help. |  |  | X | |  | F21 |
|  | **Different monitoring instruments**  Generally, patients felt that any of the presented instruments would be an improvement in existing health care, as it can help guide the discussion with the physician. Some even see its implementation as a form of good customer service. Patients had various different opinions on social influence by peers or health care professionals. A few caregivers indicated that recommendation by the treatment team specifically would encourage them to use a monitoring instrument: | **Caregiver** ‘I take that seriously, yes. Yes, of course. That… you must take it seriously. I do. Of course, it is not something you can simply wave away. I see it as an exam. No, then, yes then I will.’ (Female caregiver (67 years) of a glioblastoma patient with stable disease) Pg 3016 | X |  | |  | F22 |
|  | **Paper-and-pencil instrument**  Patients said the PCI seems to be a simple instrument that would be easy to complete because the different topics are concrete and clear, and believe that it can help recognize problems and initiate discussion . As the PCI does not allow for an indication of the severity of symptoms, patients said it only provided a snapshot picture of their concerns, which may hinder its usefulness. Patients indicated that they expected the social cues from face-to face feedback would help them in the interpretation of the advice provided and would allow them to ask questions.  Informal caregivers felt the DT included relevant topics and could help initiate a conversation about supportive care. They expected the DT to be easy to complete. Face-to-face feedback was mentioned to be highly appreciated and could make them feel acknowledged. However, restrictions of the DT mentioned include that some caregivers found it too superficial and that the answer options do not do justice to subtle fluctuations in symptoms or concerns. They indicated that questions were difficult to interpret and that it would be difficult to monitor supportive care needs over time. |  |  |  | | X | NS2 |
|  | **Computerized application: Oncoquest**  Patients indicated that Oncoquest provides a more detailed description of needs compared to the paper and pencil (PCI). The option to receive face-to-face feedback was mentioned to be an advantage, although some patients indicated that they would also like to receive feedback in written form:  Caregivers mentioned more advantages of Oncoquest. They indicated to expect that completing Oncoquest would not take long, and the answers go straight to the physician. The availability of the instrument at the outpatient clinic was seen as an advantage by some, as at this time, their focus is already on the disease. Others indicated that completing the questions from home would be better: | **Patient** ‘Preferably on paper, but I also say that because of my current… short-term memory.’ (Male grade III oligodendroglioma patient (65 years), progressive disease suspected) Pg 3017  **Caregiver** ‘Well, my first thought is not… not like, then and there. Since, as I have already said, the moments that we go to the hospital are always a little tense... and I… I am more focused on my husband.’ (Female caregiver (52 years) of a glioblastoma patient with disease progression)  Pg 3018 | X |  | |  | F23 |
|  | **eHealth application: Oncokompas**  Patients expected Oncokompas to empower the user, increasing knowledge of symptoms or concerns while allowing you to take control of your own needs. Caregivers expected Oncokompas to reduce the barrier for exploring supportive care options for themselves. Other advantages mentioned included the instant, tailored advice which could facilitate finding supportive care options, and the trustworthiness of the information provided as it comes from the hospital. Patients and caregivers felt using Oncokompas from their home computer would be convenient, as it would allow them to monitor at a time and frequency of their choosing: | **Patient** ‘I believe yes, that… that would, of course, be very convenient if you could just arrange it through the computer. […]. Then you don’t have to be there at half past ten. […] So yes, that might be even more appealing. Also because you then could do this more often. Without constantly going to and fro.’ (Male grade II oligodendroglioma patient (51 years), stable disease) Pg 3019 | X |  | |  | F24 |
|  | **eHealth application: Oncokompas**  A number of patients indicated they are less able to use computers than before, due to loss of strength in their hands, memory problems causing issues with passwords, and fatigue, language, or visual problems: | **Patient** ‘Yes, I am not someone who… who really likes to, as it were, crawl behind my computer and then... that… that actually takes quite a lot of effort nowadays.’ (Male grade IV glioblastoma patient (51 years), currently under treatment)  Pg 3020 | X |  | |  | F25 |
|  | **eHealth application: Oncokompas**  Both patients and caregivers expected completing the questionnaires online would take a lot of time. Caregivers explicitly mentioned that this would likely result in a low expected use of Oncokompas. |  |  |  | | X | NS3 |

| **Authors: Boele et al. 2017** | | | | |  |  |  |
| --- | --- | --- | --- | --- | --- | --- | --- |
| **Participants, Clinical and Demographic Characteristics** | **Findings** | **Illustrations (Page number)** | **Evidence** | | | |  |
|  |  |  | **Unequivocal** | **Credible** | | **Unsupported** | **Finding number** |
| US n=12 caregivers of PMBT  N=10 spouse, N=1 parent, N=1 cousin  Age range 30-80 years of age.  N=6 male, N=6 female  Dutch n=15 caregivers of PMBT  N=15 spouse, Age range 30-80 years of age.  N=2 male, M=13 female. | **Experiences with supportive care for caregivers**  Examples of informal support provided include social support such as being there in the hospital, supporting each other through tough times; practical support such as people coming over to bring dinner, deliver groceries, or helping out with other chores; and financial support such as helping to pay for medical bills or housekeeping. Particularly practical and financial support were mentioned more often by US caregivers, e.g.: | **Caregiver** ‘They took up a collection through a variety of different things. They had a housekeeper come in and clean our house for 6 months. For 6 months! They paid for it. […] It was tremendous.’ Husband of GBM patient who is currently under treatment. Pg 160 | X |  | |  | F26 |
|  | **Experiences with supportive care for caregivers**  In a few cases, questionnaires were used in the clinic to screen for needs. However, none reported to have received any feedback and if needs were identified, there was no further follow-up or referral to professional services. When asked if they felt the supportive care offered was sufficient, caregivers with few care issues were generally satisfied while others indicated they would have liked more information on how to deal with the patient’s symptoms and prognosis, as well as more practical, financial and social support especially early in the disease trajectory. | **Caregiver** ‘Oh I think we need something! We’re not professionals, we don’t know what we’re doing. […] Are we making it better, are we making it worse? We don’t know, we’ve not been in this situation before.’ Cousin of meningioma patient with suspected disease progression. Pg 160 | X |  | |  | F27 |
|  | **General attitude**  Caregivers generated several possible advantages of keeping track of care issues over time. It could: help them gain insight into their own needs and growing burden, so that they might get help in time; give them a sense of being in control of the situation; help them be more perceptive of changes in the patient’s symptoms; and some believed that keeping track of issues could help them process grief Disadvantages were also mentioned. Some caregivers said they do not like keeping track of things per se and many thought that just keeping track would not benefit them. Another disadvantage frequently mentioned by caregivers is that it takes time and requires discipline: | **Caregiver** ‘I don’t know if there’s a disadvantage, other than the fact that it’s time consuming. It takes time to do that and it takes discipline. You have to have discipline to sit down and journal. […] And this, I look at this company, forget whether it’s health related, if we ask people to journal, so they knew what they were spending their time on and what was most important to them during the day, we probably get about 5% compliance. It’s just, that’s a hard thing to do on a regular basis.’ Husband of GBM patient who is currently under treatment. Pg 162 | X |  | |  | F28 |
|  | **Frequency and timing**  Preferences for the frequency of monitoring care issues varied widely, ranging from once a week to once every 18 months. Many indicated that their preferred frequency would depend on the needs they experience and that it would therefore be good to start monitoring shortly after the patient is diagnosed. In terms of timing and setting, most caregivers indicated it would be best for them to complete questions at home, before visiting the hospital. This would allow more time under less stressful circumstances, and lead to more honest answers because of better privacy. Moreover, as not all caregivers were always able to accompany the patient to clinic, this would allow them to keep track of their care issues and needs more frequently. Others however, felt their often long waiting time in the hospital could be put to good use by completing questions then and there. | No illustration |  |  | | X | NS4 |
|  | **Format**  Presented with different options to keep track of their issues over time, only a few caregivers preferred a yes/no type checklist as it is easy to complete and unambiguous, while the majority of caregivers believed a multi-item scale would reflect their situation better: | **Caregiver** ‘This is just a concrete yes or no. And it could be like oh I’m having a little bit of a problem with child care or I’m in desperate need of help. And this kind of tells you how big of a problem each individual thing is.’ Wife of oligodendroglioma grade II patient with suspected disease progression. Pg 162 | X |  | |  | F29 |
|  | **Format**  A few suggested that a diary form would be best, or alternatively to allow room for free text remarks. Some emphasized that any questionnaire should be as concise as possible to reduce burden. Although caregivers said a paper-based checklist (DT) would be quick and easy to complete, includes relevant topics and would give a good overview of the issues that are present in one glance, they were unsure how they would benefit from completing the checklist: | **Caregiver** ‘Will it provide some way to foresee down times and be able to… You know, help reduce the stress to look for a way to kinda work around it? I don’t know, I’m not sure that I could… That it would have like a lot more value than ‘hmm that’s interesting’. Would it really, would I find a way to make it a less stressful time? You know based on that? I’m not sure it would.’ Husband of patient with medulloblastoma who is receiving treatment | X |  | |  | F30 |
|  | **Format**  Caregivers indicated that personal feedback from a health care professional would help, but some participants felt this would not be realistically achievable in clinical practice. Although many caregivers mentioned that an eHealth monitoring instrument that comprises a touchscreen system based in the clinic (OQ) would not be ideal as it is not mobile, they did feel completing the questions in this digital manner would be easier and faster than on paper, and better for the environment. Several caregivers said the fully digital option comprising a home-based monitoring instrument (OK) could serve as a guide to supportive care options as the resources it generates are suggested by a trusted source (i.e., the hospital). The opportunity to complete the questions at home and the flexibility in terms of timing were mentioned as important advantages: | **Caregiver** ‘I like it ‘cause you could do it at your house, whenever you want to, privacy of your home, be comfortable, and you know. It’d just be you and the computer. I like that.’ Wife of patient with astrocytoma grade II who is under treatment. Pg 162 | X |  | |  | F31 |
|  | **Format**  Negative points raised regarding the fully digital option (OK) were the expected high investment of time, an expected need for reminders, and the lack of personal contact. | **Caregiver** ‘So I think if you maybe had the answers like the short answers and then give the opportunity to say tell me what you meant by this. And then it will all come out. To give them the opportunity to not be hit by it all of a sudden.’ Wife of oligodendroglioma grade II patient with suspected disease progression.  Pg 163 | X |  | |  | F32 |
|  | **Format**  Almost all caregivers felt able to use an eHealth instrument and most, but not all, caregivers opted for either a fully digital instrument or a combination of a digital instrument with personal feedback. A few caregivers, on the other hand, preferred a paper-based checklist with personal feedback instead, and one caregiver suggested to make all options available so that all personal preferences may be reflected. | No illustration |  |  | | X | NS5 |
|  | **Conditions for use**  Caregivers made several suggestions for the development of any monitoring instrument. Many said they would not make use of an instrument unless they perceive benefits for themselves; this could be related to the needs present, or to the options for supportive care that are available and affordable. Participants said that any needs assessment should be quick to complete, preferably under 10 min, and be clear and easy to use. Furthermore they indicated that the resources that are referred to, should be tailored specifically to the neuro-oncology situation. Many believed recommendation by a trusted source such as the treatment team would help them to start using a monitoring instrument but it was expected that an incentive (either the presence of supportive care needs, a small monetary reward, or frequent reminders) would be needed to ensure continued use: | **caregiver** ‘I think some people would say yeah that’s a good thing, the doctor’s telling me that. There’s other people who’ll forget about it once they’re in the car. There has to be constant reminders. […] Just an idea, some kind of marketing pushout to people on a regular basis.’ Husband of patient with medulloblastoma who is under treatment Pg 163 | X |  | |  | F33 |

| **Authors: Cavers et al. 2012** | | | | |  |  |  |
| --- | --- | --- | --- | --- | --- | --- | --- |
| **Participants, Clinical and Demographic Characteristics** | **Findings** | **Illustrations (Page number)** | **Evidence** | | | |  |
|  |  |  | **Unequivocal** | **Credible** | | **Unsupported** | **Finding number** |
| N=26 patients with PMBT  Age range 21-66 years. N=13 males, n=13 females.  N=23 caregivers, age ranged not reported. N=19 spouse, N=3 parents, n=1 daughter | **Dynamic physical trajectory**  Initial physical problems with which patients presented varied from a sudden isolated seizure with rapid diagnosis, to more gradual symptoms including headache and nausea: | **Patient** The little funny turns which were almost like, it felt to me, almost if I was having the start of a seizure … Just a bit of a shuddering in my head and wanting to just be lying down. But never ever lost consciousness or anything like that. Just, you know, I was working. I was scared, I was very scared. — Sandra, 46-year-old woman with a suspected glioma, interview before diagnosis (time 1) Pg 376 | X |  | |  | F34 |
|  | **Dynamic physical trajectory**  Physical and cognitive symptoms tended to increase in number and severity with time, with a substantial impact on patients’ strength, mobility, communication, understanding, behaviour and appearance. Problems with memory, speech and language could become particularly distressing: | **Patient** My memory is not the same, not the same as what it used to be like … trying to read things, you just, you read a wee bit and then you get fed up with it. Concentration is not there either. — Robert, 36-year-old man with glioblastoma multiforme, interview after diagnosis (time 2) Pg 376  **Caregiver** When you are starting a sentence, er, and you can’t think of the right word and you come out with some, you tried desperately. You know and I can see you being annoyed with the situation intensely because you can’t think of the right word. — Alistair, husband of Harriet (64-year-old woman with glioblastoma multiforme), interview after treatment (time 3) Pg 376 | X |  | |  | F35 |
|  | **Dynamic physical trajectory**  Most patients had a period of stability before their tumours gradually progressed, leading to more debilitating physical symptoms and cognitive decline. | **Patient** Sometimes I get a bit tired … I think, the first few [treatments] I didn’t really feel it. But the last couple I’ve felt a bit tired. — Ewan, 21-year-old man with glioblastoma multiforme, interview during follow-up (time 4) Pg 376 | X |  | |  | F36 |
|  | **Dynamic physical trajectory**  Physical symptoms increased in number and severity as death approached | **Caregiver** And then eventually all the seizures started coming back again. And they were just like happening all the time. There was one weekend, he had 26, it was just like unbelievable. — Sheila, wife of Andrew (45-year-old man with glioblastoma multiforme), interview post bereavement (time 5) Pg 376 | X |  | |  | F37 |
|  | **Dynamic social trajectory**  The lives of patients and their caregivers were substantially disrupted at the time of diagnosis. Their time was occupied with in-patient stays, visits to hospital and recovering from surgery, leaving little time to invest in their social wellbeing. However, many people gained support from family and friends visiting them. While waiting for her diagnosis (time 1). | **Caregiver** So [patient] could ask questions because as I say there’s things he wants to know. And she was very helpful as well and very positive as well about the whole thing. That was very helpful. — Sheila, interview at time 2.  Pg 376 | X |  | |  | F38 |
|  | **Dynamic social trajectory**  For most people whose disease was stable, there was a strong desire to return to “normal” socially after treatment (or a “new normal,” depending on abilities). For some patients, the effort of socializing posed a barrier to returning to life as normal. | **Patient** We stopped doing that because it’s … likes the pubs and all that sort of stuff, just gave up that because you cannae [cannot] really get into that at all. — Andrew, 45-year-old man with glioblastoma multiforme, interview at time 3  Pg 376 | X |  | |  | F39 |
|  | **Dynamic social trajectory**  Caregivers found it difficult to continue to work and participate in other activities; some identified a change in gender roles. | **Caregiver** It was at least an hour later than I really wanted to depart (for work), when I got finished. Erm, doing household jobs and what have you … Erm, I’ve no doubt other things that might tire me out and wear me out, like erm, organizing the washing, drying of clothes. — Alistair, interview at time 3. Pg 376 | X |  | |  | F40 |
|  | **Dynamic social trajectory**  As the disease progressed, the sense of isolation increased. | **Patient** I’m seeing people in a different context, but they are kind of one-off things as opposed to like a normal social life. … ‘Cause, I mean, I, the types of stuff I was doing that I mentioned earlier, the exhibitions, you know, book shops and things, I’m just not able to do any more. So, you know, gradually I suppose, I’ve been cut off from society that way. — David, 48-year-old man with a brain stem glioma, interview at time 4  Pg 376 | X |  | |  | F41 |
|  | **Dynamic social trajectory**  For those interviewees who practised a faith, meeting church members was a valued source of social, emotional and spiritual support. | **Caregiver** There’s a great boost from all these different people plus the fact of the, the people in [town] that go to the church in our part of the community there, I mean, they’re very, very supportive. — Audrey, wife of Bill (63-year-old man with glioblastoma multiforme), interview at time 4. Pg 376 | X |  | |  | F42 |
|  | During the terminal phase of the illness, people’s social lives dwindled alongside their physical and cognitive abilities. Often, patients became house- or bed-bound and were unable to communicate. |  |  |  | | X | NS6 |
|  | **Dynamic psychological trajectory**  Before formal diagnosis, with uncertainty at its peak, most patients felt an immediate drop in their psychological well-being. Patients’ anxiety was most acute while waiting for a formal confirmation of a malignant growth. | **Patient** I was scared when they told me, when they said there was a tumour. It knocked me for six. I thought, oh my God, I’m gonna die? — Ian, 46-year-old man with glioblastoma multiforme, interview at time 1. Pg 377  **Patient** I wanted to know, what, have I got three months, have I got six months, have I got a year? Or does anybody know? — Sarah, 66-year-old woman with suspected glioma, interview at time 1. Pg 377 | X |  | |  | F43 |
|  | **Dynamic psychological trajectory**  Caregivers reported great stress in caring for their loved ones while waiting for the diagnosis: | **Caregiver** Cos the the pressure is really enormous, you know, enormous. But it’s, I’ve never, I never visualized in my life, having to do anything as difficult as what’s happening now you know. — James, husband of Sarah, interview at time 1 Pg 377 | X |  | |  | F44 |
|  | **Dynamic psychological trajectory**  During the initial treatment period, when patients received radiotherapy and attended regular clinics, anxiety generally lessened as patients were assured by caring clinicians: | **Caregiver** He is such a warm person. And makes you somehow just, you can trust him. You know that you are in very good hands and he kind of radiates that presence. So I think that affects how, when we were talking about this. — Joan, wife of Malcolm (43-year-old man with anaplastic astrocytoma), interview at time 2  Pg 377 | X |  | |  | F45 |
|  | **Dynamic psychological trajectory**  Indeed, participants placed a great deal of importance on the style of communication used by clinicians. | **caregiver** It does help a lot, you know, it helps to make the patient feel better as well actually. You know and they can sort of communicate with somebody and have a smile. — James, interview at time 1 Pg 377 | x |  | |  | F46 |
|  | **Dynamic psychological trajectory**  During treatment, most patients strongly desired to return to normal and to make the most of their remaining time, aware that progression of their disease was likely. | **Patient** I think for me, because, because I’m that good I just keep going on as normal until I really do feel no well. And then I can start changing. — Lois, 50-year-old woman with glioblastoma multiforme, interview at time 2. Pg 377 | X |  | |  | F47 |
|  | **Dynamic psychological trajectory**  Anxiety and concerns about life expectancy, future pain and loss of control at the end of life were voiced at this time. | **Patient** That’s what I said earlier, you know, when the pain starts, what do they do? When I start losing my faculties, what do I do? I don’t know. — Wilson, 58-year-old man with glioblastoma multiforme, interview at time 2  Pg 377 | X |  | |  | F48 |
|  | **Dynamic psychological trajectory**  During the interviews at time 3, after treatment, anxieties were often expressed by patients. | **Patient** What are the things that could happen? I mean, what, is it going to be a stroke or is it going what? It might be a bit morbid to start thinking what will it be? — Henry, 65-year-old man with glioblastoma multiforme, interview at time 3 Pg 377 | X |  | |  | F49 |
|  | **Dynamic psychological trajectory**  For caregivers, dealing with progressive personality changes was most distressing. | **Caregiver** It’s made it sort of, unbearable sometimes … It’s hard to imagine being able to live the rest of my life with somebody with that kind of temperament. — Sharon, wife of Ian (46-year-old man with glioblastoma multiforme), interview at time 3, Pg 377 | X |  | |  | F50 |
|  | **Dynamic psychological trajectory**  Some caregivers showed symptoms of depression, particularly if they had a history of the disease. | **Caregiver** I have been depressed in the past so perhaps that is why I am feeling more depressed than I would like. But it’s just, I hadn’t really decided what to do, you know, about getting [anti]depressants, I don’t know. — Joan, interview at time 3, Pg 377 | X |  | |  | F51 |
|  | **Dynamic psychological trajectory**  For some patients and their caregivers, the distress they felt that was related to the fear of dying eased with time. | **Caregiver** Alice: And I think we’re beginning to be, well I am, less frightened of it [dying]. Henry: Yep. I’m not, I have to say I am not frightened of it. — Henry (65-year-old man with glioblastoma multiforme) and his wife Alice, interview at time 3. Pg 378  But it’s now over a month since it stopped and he’s just going from strength to strength and it’s wonderful. This is like a sort of honeymoon period, if you like, you know, until they take the scan again. — Angie, wife of Wilson, interview at time 3. Pg 378 | X |  | |  | F52 |
|  | **Dynamic psychological trajectory**  During follow-up interviews, worries about the cancer’s recurrence persisted: | **Patient** I think, probably, that it’s just normal and I am fine. But at the back of my mind, always the thought, now is this the cycle starting again. — Wilson, interview at time 4. Pg 378 | X |  | |  | F53 |
|  | **Dynamic psychological trajectory**  The progression of disease could cause a decline in mental well-being, but knowing the prognosis from the start meant patients had time to prepare for this eventuality: | **Patient** You don’t know how this deterioration ends up, how bad it might be or whatever.You know, because I don’t want to, you know, become a, a complete burden on my sister. — David, interview at time 4. Pg 378 | X |  | |  | F54 |
|  | **Dynamic psychological trajectory**  The support of clinicians at this time was crucial, and a lack of such support was distressing. | **Patient** This was vocalized by Wilson, who said “Yes, I mean, basically I just, well, [doctor] didn’t have any sort of bedside manner ... it was terrible.” Pg 378 | x |  | |  | F55 |
|  | **Dynamic psychological trajectory**  Distress could increase again as thoughts about death became pressing. | **Patient** Three weeks before he died, Sandy (a 47-year-old man with glioblastoma multiforme) became increasingly frustrated, to the point of wanting to die, stating “I’ve kind of made up my mind that it’s better to be going than live like this.” Pg 378 | X |  | |  | F56 |
|  | **Dynamic existential trajectory**  The unconfirmed possibility of a fatal brain tumour brought many patients to immediately question the meaning and purpose of their lives. Where a sense of meaning was absent, existential distress often occurred. | **Patient** I was looking for answers and I haven’t got any. I’ve tried to get answers to some of my questions and it hasn’t come yet. — Sarah, interview at time 1, Pg 378 | X |  | |  | F57 |
|  | **Dynamic existential trajectory**  The crisis made some patients more spiritually aware, with several turning to religion. | **Patient** You can find yourself getting quite spiritual in situations like this too though. I think you’re feeling a bit more desperate and very vulnerable. — Sandra, interview at time 1. Pg 378 | X |  | |  | F58 |
|  | **Dynamic existential trajectory**  General sources of existential support were available,such asfriends, family and professionals. | **Patient** Oh definitely. Yeah, I mean … I mean if I never had her I’d be, really be struggling. — Andrew, interview at time 1, Pg 378 | X |  | |  | F59 |
|  | **Dynamic existential trajectory**  Existential distress appeared to lessen with time. Everyday life was more valued, and patients became more at peace with their situations in subsequent interviews: | **Patient** Every day is a bonus. And you wake up in the morning and say well, I’ve got another day? …You look at life and you think, ‘Yes’. — Ian, interview at time 2. Pg 378 | X |  | |  | F60 |
|  | **Dynamic existential trajectory**  Facing a life-limiting illness led many patients to adjust their priorities, | **Patient** I’m looking here and I’m thinking what are we pushing for all of the time? Sometimes you should actually just sit back and enjoy what you’ve got and relax … So I think that’s my kind of motto now, like. — Robert, interview at time 2  Pg 378 | X |  | |  | F61 |
|  | **Dynamic existential trajectory**  Just as at diagnosis, a firm faith in God provided spiritual comfort for some patients as their illness progressed: | **Patient** I believe there’s life after death … so that way I’m not frightened of dying … It calms me down. I know whatever happens, when it happens, will be the Lord’s decision, not mine. — William, 64-year-old man with glioblastoma multiforme, interview at time 2. Pg 378 | X |  | |  | F62 |
|  | **Dynamic existential trajectory**  As time passed, people accepted their situation as part of an ongoing process: | **Patient** … And then you say well why not me. Somebody’s, people get ill … that’s life. I’m doing better with myself with accepting it. — Bill, interview at time 3. Pg 378 | X |  | |  | F63 |
|  | **Dynamic existential trajectory**  However, some participants described a lack of professional support in maintaining their hope. | **caregiver** For example, Sheila, in her interview at time 3, stated “I wanted them to be more positive for us.”Pg 378 | X |  | |  | F64 |
|  | **Dynamic existential trajectory**  Gradually, patients were able to prepare for and accept the prospect of dying. Some participants developed a greater appreciation for nature, whereas others were simply resigned to their deaths: | **Patient** I get up early now and I like to look out at the day break you know and see the sun coming out over there it’s good, I enjoy that. Nice and quiet and have my thoughts and think that’s nice that’s, life could be a lot worse, yeah. Just thinking what a wonderful world you know. — Bill, interview at time 3. Pg 379  **Patient** I am fairly pragmatic about … life and death. Everyone is as important as everyone else. But ultimately we’re all insignificant is really how I regard it. — David, interview at time 4 | X |  | |  | F65 |
|  | **Dynamic existential trajectory**  Existential sadness and distress were sometimes expressed alongside finding meaning and peace in the journey toward death. There appeared to be a swaying between hope and despair as participants struggled to maintain positive attitudes. | No illustration |  |  | | x | NS7 |

| **Authors: Collins et al 2014** | | | | |  |  |  |
| --- | --- | --- | --- | --- | --- | --- | --- |
| **Participants, Clinical and Demographic Characteristics** | **Findings** | **Illustrations (Page number)** | **Evidence** | | | |  |
|  |  |  | **Unequivocal** | **Credible** | | **Unsupported** | **Finding number** |
| N=23 caregivers of patients with PMBT  (n=15 current and n=8 bereaved).  N=10 spouse, N=3 child, N=2 other  ranged in age from 27 years to 77 years (median 54 years) | **The enormity of the caring role**  Carers described the physical and emotional enormity of providing care, which differed according to the patient’s level of disability, but overall involved doing ‘everything’. Constant availability to tend to patient needs was required: practical help with toileting, feeding, bathing and dressing; driving patients to required appointments; advocating for the patient; navigating the health system; making treatment decisions; managing medication schedules, and for some, tumour-related sequelae such as seizures. This was all undertaken while accepting new responsibilities previously shared with their partner; bearing financial strain as a single-income provider; and managing the reactions and emotions of concerned family and friends. | **caregiver** “For me it was a massive upheaval… my life did a complete turnaround.” (C14)  **caregiver** “It’s all changed. Everything’s changed.” (C2)  **caregiver** “From that moment [of diagnosis], everything was different… As the seizures progressed, she started losing more of her abilities – she lost the ability to eat, to drink, to stand, to walk. Her sanitary needs were done by me, everything was done by me.” (C3)  Pg 5 | X |  | |  | F66 |
|  | **The challenges brought about by the particularities of PMG— neurocognitive changes and the unique illness trajectory**  Neurocognitive problems were reported to be a significant cause of concern for carers who described difficulty and frustration in managing personality changes; diminished insight and inhibitions; and impulsive, erratic, inappropriate or aggressive behaviour changes. Such changes and the additional particularities of the PMG illness trajectory were recognised to cause carers significant burden. The rapidity of change from ‘normal’ state to crisis following the cancer diagnosis and each period of recurrence was always unexpected. Despite being told of the patients’ poor prognosis, the uncertainty and unpredictable deteriorations often meant carers felt unprepared for their role. | **caregiver** “We have lots of fights continually, because she thinks she’s right. ‘No, you can’t put your hand in the flame,’ ‘Yes I can.’ Stuff like that. There’s no rationalisation behind it; two and two do not make four. That’s the most frustrating thing about it.” (C3) Pg 5 | X |  | |  | F67 |
|  | **The complexity of living with the unspoken future**  Trying to negotiate hope in the face of increasing deterioration caused an emotional burden for carers who were committed to caring, without any indication of how long it must be sustained. The complexity of this was reflected in the discrepancy of views between carer groups regarding the need for early referral to palliative care. Bereaved carers almost universally reported the benefits of referral to palliative care, and felt that information about these services should be introduced early in the patient’s illness. Conversely, current carers who had not yet had contact with palliative care often reported they didn’t think they needed help, and didn’t want to think about it until it was necessary. They instead, emphasised the importance of always maintaining hope. Current carers who were interviewed while the patient was in a palliative care unit however, reported relief that the patient was being cared for and wondered why ‘no-one had told them’ about palliative care. Thus it was apparent carers ‘don’t know what they need to know’, but with hindsight, felt it would have been better to have been prepared and supported ahead of time. | **caregiver** “We’re realistic enough, we know one day this thing will creep up on us and that’s it – but you don’t want to hear that, you want to keep going and tracking on until such time as well, it goes wrong I suppose.” (C6)  **caregiver** “I would have liked it if [the oncologist] had said to [the patient], “This is what you need now and I want you to get into the program… Just to be in the system much earlier than when you land in the system…” (BC6)  **caregiver**  “I think to be in contact with palliative care at the early stages would be something positive for everybody… Because then when you want something you’ve got a specialist person that’s got the knowledge to be able to talk to the family about what’s happening.” (C12)  **caregiver** “I think a lot of people have no idea what they’re up against… I know that sometimes it’s cruel for medical staff to have to tell you how it is but I think you just can’t hide that stuff from people.” (C13) PG 5 | X |  | |  | F68 |
|  | **Lack of care coordination and continuity**  Carers described the absence of a central, clearly identified contact person who was responsive, reliable and available. Necessary coordination tasks identified by carers that were unfulfilled included: providing a point of contact within the treating hospital; assisting in navigation through community and hospital settings; providing information; and being a familiar presence who was aware of the whole person when there was frequently little continuity of medical care. Many bereaved carers noted community palliative care provided these tasks later in the illness. | **caregiver** I had nobody to talk to, no contact, I didn’t know what to do.”(C14)  **caregiver** “We were just being handballed around. No one was going to take responsibility and tell us what we had to do.”(BC8)  **caregiver** “There’s no continuity, there’s no one doctor. She spent a month remembering the doctor’s name from last visit. And then when we get there and it’s not him.” (C12) PG 5 | X |  | |  | F69 |
|  | **Lack of individualised information**  Carers emphasised the inadequacy of resources provided to support them in their caring role. Written information was scarcely given, and never individualised. Consistently identified gaps included: what to expect over the patient’s illness; how specific tumourrelated sequelae manifest; where to seek additional help if required; specific seizure information; practical tips for caring and strategies for managing behavioural and personality changes. Carers stressed the importance of understanding the relationship between patient’s behavioural changes with the site of their tumour. Overall carers noted that often their needs for information differed with those of the patient and changed over the patient’s illness. | **caregiver** “I wish somebody had have sat down and said to me, ‘Do you want to know why your husband’s acting like that, or why he doesn’t want to have a wash?’ And I would have said, ‘Yes, I do want to know.’ But you have to get a bit of a shove.” (BC2)  **caregiver** “You actually need an interpreter to be with you, you kind of need that middle person… That go to person like a librarian, who can say, ‘This is the information you need.’ You’d hit the next bump in the road and every time was different and every time I hit that bump I just didn’t have that ‘go to’ person to ask.” (BC6) PG 5 | X |  | |  | F70 |
|  | **Lack of preparation**  The limited preparation by the health system to assist carers to effectively undertake their role was emphasised. They felt unsure of how to enlist support afterhours, often defaulting to emergency departments for simple advice. Carers were particularly dissatisfied with the lack of preparation at key transitionary points such as at diagnosis, at discharge following surgery, or following tumour progression. At such times, although recognising the constraints of busy clinics, carers felt they required better preparation to appropriately care for the patient. | **caregiver** “I certainly needed help with the physical management, like even finding out that you could put a tap at the end of the urinary bottle so it wouldn’t spill everywhere… Things like that I think ‘The things you learn!’ It sounds so obvious, but if you don’t need to know, how would you ever know?” (BC5)  **caregiver** We weren’t informed about having seizures… The doctors had said if you fall unconscious then someone should call an ambulance…. No mention about length of time of seizures, you know, at what point is too long?” (BC8) Pg 5 | X |  | |  | F71 |
|  | **Lack of emotional support**  There was typically no routine emotional support provided to carers (or patients), and if it was offered, the demands of the caring role made access difficult. Carers reported support was needed to: validate experiences and legitimise concerns; assist with everyday caring and decision-making; provide strategies; and have their grief heard. Carers suggested an ‘online buddy system’ or a telephone ‘lifeline’ might be an accessible format to fill the void of face-to-face support. This was particularly important during bereavement, when carers report immense loneliness in their grief. | **caregiver** “You need someone who is consistent and persistent in contacting you as the carer, and for want of a better word, the victim, of what’s going on –because you not only live through it, but you have to tolerate it, and keep going with a smile on your face” (BC6) PG 5 | X |  | |  | F72 |
|  | **Inconsistencies of service provision**  Carers reported inequity in service provision, where some had access to the full range of supports available, and others, very little. Those who lived in regional or remote areas emphasised the lack of services available to support them and some relocated to the city for significant periods to receive treatment or required services. Inadequate communication with local medical teams about the patient’s treatment plan was also recognised by rural carers. Bereaved carers in particular, advocated the need for more timely access to community support services, often referring to long waiting times for respite, which exceeded the patient’s prognosis. Access to inpatient palliative care was reported by some carers to be restricted, due to the uncertainty of a glioma prognosis with few hospices that could offer extended care. They felt that nursing or aged care homes were inappropriate places of care for patients with glioma and were deeply distressed if patients were required to move from an inpatient palliative care environment to a nursing home. | **caregiver** ‘When we got into the public that’s when everything became so much more manageable – we were getting a lot more services and, we just felt it was better managed- a lot more cohesive – ‘we were much better informed.’ (C10) Pg 5 | X |  | |  | F73 |
|  | **Relentlessness of caring**  Carers described the impact of undertaking their role and responding to the difficulties as ‘relentless’, but did so with a continued sense of loyalty. The lack of choice in accepting the role, the inability to share the caring load, and the limited time for themselves was a continual struggle as carers persisted in prioritising the patient’s needs above their own. Carers with young families spoke of the ‘massive upheaval’ to their family, as children were often left to ‘fend for themselves’. While current carers reported feeling helpless, they frequently downplayed their distress. Bereaved carers however, were able to openly verbalise their own depression, their feelings of inadequacy in caring, and the toll it exacted upon their physical health. | **caregiver** “Occasionally it overcomes you… It’s relentless, it’s nothing but relentless.” (C3) PG 6 | X |  | |  | F74 |
|  | **Loneliness of caring**  Carers frequently reported they had lost their support network, who had ‘dropped off’ while they were consumed by the daily tasks of caring. The sense of isolation was made worse by the subtle cognitive changes which often occurred, yet were unnoticed by others. Such changes meant the nature of their loving relationship was different, and carers were now facing a new phase of life effectively alone. The nature of such changes often meant patients would refuse help from people other than their carer. Likewise carers reported trying to ‘uphold face’ and protect the patient from outside judgement. Carers of those patients with such profound changes commonly reflected on their grief for the loss of the person they loved, for their former relationship and ‘pre-caring life’. The demands of caring under these circumstances were compounded by having to ‘make allowances’ for the differences | **caregiver** “You get glimpses of the old Jack, but he feels different to me…I feel like I’ve already lost him.” (C2)  **caregiver** “Of course I couldn’t talk to her about it [dying], because she didn’t have the words to deal with the impact of what I’d be telling her. She couldn’t express her rage, grief, her desperation. So I wasn’t sure whether she did know [she was dying], or she didn’t.” (BC4)  **caregiver** “Death is like divorce in a sense; you had a huge network of friends and all of a sudden you’ve lost your left arm and you’ve lost everybody that goes with that left arm and it’s horrid.” (BC6) PG 6 | X |  | |  | F75 |
|  | **The ongoing suffering for bereaved carers**  Despite reflecting on some moments of joy and good humour in caring; overall there was much grief, and this was sustained into bereavement. Bereaved carers spoke of the long ‘catch-up’ period after the patient died, in which they finally had time and space to process the immense losses. They spoke of their sadness at missed opportunities for sharing meaningful goodbyes, which they had expected at their partner’s impending death, but were not possible in the setting of cognitive failure. They ruminated over regrets about care, particularly when the patient who expressed a wish to remain at home, had died in hospital. Some carers still felt distressed by ‘unanswered questions’ about the patient’s illness, attributing behavioural changes to the person being different, rather than recognising such changes as tumour-related sequelae. | **caregiver** “I apologised and said, ‘You have to go [to hospital], I can’t do this anymore.’ And then he wouldn’t talk to me, and it was really hard…The promise was he’d stay at home for as long as possible, and I just couldn’t go another minute.” (BC6)  **caregiver** “It’s funny but the more time passes, the more I need support… I’m floundering still… but I don’t have anyone who wants to listen.” (BC2) Pg 6 | X |  | |  | F76 |

| **Authors: Coolbrandt 2015** | | | | |  |  |  |
| --- | --- | --- | --- | --- | --- | --- | --- |
| **Participants, Clinical and Demographic Characteristics** | **Findings** | **Illustrations (Page number)** | **Evidence** | | | |  |
|  |  |  | **Unequivocal** | **Credible** | | **Unsupported** | **Finding number** |
| N=16 caregivers of patients with PMBT  Mean age 54.2 years (31-68 range),  N=6 male, N=10 female  N=13 spouses, N=2 parents, N=1 friend | Feeling lost and alone in a new life | **caregiver** We had never thought of such a thing. We had never thought about something like that. What happened afterward (after the announcement of the diagnosis), I can’t remember. I was like, ‘‘this is not possible, this can’t be true; this cannot be’’  (crying). (C10) page 408  **caregiver** We were poorly supported. There you are: completely powerless. They told us, ‘It is big, it is malignant,’ and at that time, it seemed like he had only 6 more months to live. So, then he came home, and we had to wait for the (results  of the) biopsy. And then, well, you really don’t know what to do anymore, you feel desperate. It is really the way you are supported actually, the assistance you get. (C11) page 408  **caregiver** And then you see someone who is enjoying life, suddenly stuck in a harness, not being able to do anything anymore.  And that hurts. That really hurts (silence). (C8) page 408 | X |  | |  | F77 |
|  | Committed but struggling to care | **caregiver** ‘‘I shall continue doing it myself until I fall down when it comes down to it.’’ Page 409  **caregiver** If my husband gets to sit in a wheelchair (I) then, maybe, we will need to have an elevator to go upstairs, you knowI. Then I think, ‘‘Oh dear, is this what comes next? I can’t manage that.’’ That may be very terrible, and maybe, when I’m actually in that situation, somebody needs to do it, you know. And everyone says, when that is actually happening, then you are maybe able to do that, because you  know, that is what is in front of you and what you just have to do. (C15) page 408 | X |  | |  | F78 |
|  | Caring needs | **caregiver** Nobody wants or dares to tell you what is going to happen, because indeed, it depends on the patient, but somehow you really need to know. (I) Luckily, I had read on that Web site about what can happen; I was prepared to so  many things, because those last months were really hard. He stood up in the middle of the night, and he was convinced that it was the day. Luckily, I knew from that Web site that this could happen. (C16, during an interview after death of the patient) page 410  **caregiver** That was the most important thing for me: that I would know whom to turn to with questions and not to stand there likeI, ‘‘And now I’m still alone here and what do I need to do now? Whom can I call?’’ (C10, during an  interview after death of the patient) page 411 | X |  | |  | F79 |

| **Authors: Cubis et al 2022** | | | |  |  |  |  |
| --- | --- | --- | --- | --- | --- | --- | --- |
| **Participants, Clinical and Demographic Characteristics** | **Findings** | **Illustrations (Page number)** | **Evidence** | | | | |
|  |  |  | **Unequivocal** | | **Credible** | **Unsupported** | **Finding Number** |
| 20 Participants; 65% female  aged 22–69 years with diverse types of primary brain tumour (25% grade I, 25% grade II, 20% grade III and 30% grade IV or malignant) who were on average 35 months post-diagnosis | Engaging and connecting | *Barriers:*  **Patient** Cognitively um like … the other day I was already over at the coffee shop with another friend and Suzie walks in with hands on hips like ‘Angie! Did you forget we’re meeting for coffee? (Angie, Time 1) Pg 10  **Patient** It can be painful for [friends] because I’m just not getting the message … I usually just shut myself off from conversation. Sit back a little bit and lean back just so they know I’m not – I don’t make myself involved. (Paula, Time 1) Pg 12  **Patient** Yeah since the tumour, somebody I grew up with, who has known me since I was a baby, actually said she’d seen another good friend pass away and she can’t handle seeing that happen again. (Lou, Time 1 & 2) Pg 12  *Facilitators:*  **Patient** My dad, grandma and [mum’s friend] make sure I remember to contact my friends. If they don’t remind me, I’ll forget what we have planned. (Ciara, Time 2) Pg 13  **Patient** I have [husband] to help me so much with some of the stuff I can’t think through or talk through … other people are lovely and accommodating, but the flip-side is it can come across as condescending. (Paula, Time 1) Pg 13  **Patient** See, I can’t visualise when I’ve got to go down the stairs, I’ve got to have a rail ‘cos I have to take great care in walking down the stairs. (Lou, Time 1) Pg 13-14.  *Strategies:*  **Patient** I’ll generally try and make sure that if I have a big day and I’m catching up with people I make sure that I allow for some time to slow down and just rest the head. Don’t have to sleep but rest the head a bit and ah … have that down time, which I need I suppose. (Max, Time 1) Pg 11    **Patient** It’s hard for me to keep up with conversations with more than one or two people, so I generally have coffees or dinners with one or two people instead of big groups. (Ella, Time 2)  Pg 11  **Patient** When I meet somebody for the first time now, I just tell them, that I have brain cancer and that is why I might come across a bit strange. That way they understand better. (Lou, Time 1) Pg 11  **Patient** There were always people around; I made sure to go through the main roads. There were always loads of people around in case anything happened. (Frankos, Time 1) Pg 14  **Patient** On the website I post something every month or so, then with Facebook and Instagram… I don’t have to call people to catch up all the time – they’ll message me asking when I’m free to catch up. (Max, Time 1) Pg 14  **Patient** They said, ‘oh ***, what do I say to someone in this situation?’ And I was conscious of that and trying to just sort of help them get over that by saying ‘hey! I’m still here. I’m still who I always have been. Let’s just move on’. (Peter, Time 1) Pg 14  **Patient** … a lot of my existence is around that rehabilitation and how I am coping. But it’s certainly not central to other people’s lives. I need to be conscious of letting them know how I am. But also, be ready to flip it over to find out what they’re up to as well. (Peter, Time 2) Pg 15  **Patient** It’s been hard just everyone else is off working and studying. I’d like to branch out and make some new friends, but I don’t know where to start. (Ciara, Time 1) Pg 15  **Patient** This TAFE course I am going to do is like a stepping-stone. For me to just get out there and meet new people. (Ciara, Time 2) Pg 15 | X | |  |  | F80 |
|  | Then versus now | **Patient** My work colleagues were never regular friends, but I enjoyed their company. Now I don’t work so I meet people from work for coffee, you know. It helps me stay in the loop. (Will, Time 1) Pg 11  **Patient** Yep, we still catch up for coffees. We don’t talk as much about work which has been good. (Will, Time 2) Pg 11  **Patient** And, so it wasn’t like I felt I had to actually bring them in, like they were sort of there the whole way. And nothing really changed. (Jake, Time 1) Pg 16  **Patient** Yeah, yep. It’s all pretty much as it was few months ago. That hasn’t changed. (Jake, Time 2) Pg 16  **Patient** Yeh I still see all of them, they come and visit or I meet them for coffee downstairs at the café … The brain tumour support group has become like a family. And the Occupational Therapist. Really important. (Belle, Time 1) Pg 11  **Patient** It [social map] would still be pretty much the same. I’ve been doing music therapy so that would be a new group too. (Belle, Time 2) Pg 11  **Patient** well, now I’m part of the choir at the wellness centre and that’s opened up my world even more. (Lou, Time 1) Pg 16  **Patient** …the surgeon came on his rounds and I had already asked the junior doctors if I might be allowed go over to the Wellness Centre … then you know I burst into the room and I got such a lovely response. (Lou, Time 2) Pg 16-17  **Patient** It was hard at first because I couldn’t work or study. Apart from my parents and [partner] I couldn’t get out to do things…. (Ella, Time 1) Pg 11  **Patient** I’m working a few part time hours at the moment to see how I go… I might go back to study soon too. It’s been good to make new friends. (Ella, Time 2) Pg 11  **Patient** Um, wasn’t until after I had surgery and I started studying and leaving the house that I started to build a base of friends again, from scratch… (Roberta, Time 1) Pg 17  **Patient** Um. I have more uni friends, ‘cause people know who I am. My other ‘uni people’ groups have expanded a bit. (Roberta, Time 2) Pg 17  **Patient** I’ve definitely lost touch with people since I’ve been sick. No-one wants to know you when you can’t be at their beck and call. (Brione, Time 1) Pg 11  **Patient** …it really upsets me because nobody even bothers to check anymore. Nobody really keeps in touch. They go on with their own lives and my life just gets smaller and smaller. (Brione, Time 2) Pg 11  **Patient** I hardly see them so that’s the thing, people are all busy, busy, busy, busy so I don’t see many people really. (Irene, Time 1) Pg 17  **Patient** Ah only a few people now, you know the ones that are closer, the rest have forgotten about me. (Irene, Time 2) Pg 17 | X | |  |  | F81 |

| **Authors: D’Agostino and Edelstein 2013** | | | |  |  |  |  |
| --- | --- | --- | --- | --- | --- | --- | --- |
| **Participants, Clinical and Demographic Characteristics** | **Findings** | **Illustrations (Page number)** | **Evidence** | | | | |
|  |  |  | **Unequivocal** | | **Credible** | **Unsupported** | **Finding Number** |
| N=7 young adult survivors of PMBT  Age range 20-32 years  N=2 male, N=5 female | Specific Challenges: Brain Tumor Diagnosis. Psychosocial cognition, living with a brain tumor | **Young adult survivor** Sometimes I will freeze when I start talking ... like I’m notorious, for example, for going off in tangents like you can tell, I probably talked about seven different things in the last 10 seconds. Page 593  **Young adult survivor** When I was going through school ... people would say things to me and I would be all excited and reliable and honest and everything and then they would just back out and they wouldn’t show up and I was like what am I missing here? Page 593  **Young adult survivor** I’ve gone 41 /2 years now with no recurrences and so like everyone thinks you’re cured and every six months when I get my checkup it’s kind of like a roll of the dice, you have no idea what’s going to [happen]. Page 594 | X | |  |  | F82 |
|  | Practical: Limited options, loss of autonomy | **Young adult survivor** Finding a job is the hardest thing, like I can go to McDonalds, but I want something higher up for the future like maybe in an office or something, a real job. Page 594  **Young adult survivor** I lost my [driver’s] license last year because I had another seizure. Page 594 | X | |  |  | F83 |
|  | Psychosocial resources: peer support, professional counselling | **Young adult survivor** When you’re freaking out like that you probably just want a survivor who can relate to you more than like a doctor who will give you cold hard facts. Page 594  **Young adult survivor** I had one person who was my age who’d been through cancer too and gave me lots of information and it was fantastic because it’s not just about where you are in your life, it’s about where you are mentally and emotionally. Page 594  **Young adult survivor** Maybe when you are assigned to an oncologist you also get like assigned to you a psychosocial oncologist. Page 594 | X | |  |  | F84 |
|  | Health care delivery: Probing for symptoms, age-specific information, education | **Young adult survivor** I would have annual checkups and the doctors would ask is everything ok and for me everything was ok, but it wasn’t ok, I just didn’t know that it wasn’t ok. Page 595  I would’ve liked stats from my age group rather than overall because overall stats were horrible, but when you’re in your 20’s, it’s a much better [prognosis]. Page 595  You absolutely need some kind of group involving parents and explaining things to them ... so that not everything they hear is coming from you ... to have somebody with you know MD or PhD just kind of lending some support. | X | |  |  | F85 |

| **Authors: Dahlberg et al 2022** | | | |  |  |  |  |
| --- | --- | --- | --- | --- | --- | --- | --- |
| **Participants, Clinical and Demographic Characteristics** | **Findings** | **Illustrations (Page number)** | **Evidence** | | | | |
|  |  |  | **Unequivocal** | | **Credible** | **Unsupported** | **Finding Number** |
| 7 persons living with brain tumours comprised of 5 male, 2 female. Age range 34yrs-66yrs. Time since diagnosis 5yrs-19yrs.  Total of 12 caregivers:  6 informal, all female. 4 spousal and 2 child caregivers. Age range 25yrs-88yrs.  6 bereaved, 5 female and 1 male. 2 spousal, 3 child and 1 sibling caregivers. Age range 27yrs-54yrs | Perceived usefulness of the CareMaps tool | **Caregiver (bereaved)** Well, the first impression was … you don’t understand how many relations you have. And there are many relations that perhaps you didn’t think of and how those have changed. So that can be a lesson learned in itself. Also, […] you can be angry […] because you think people have failed you or … so when you think about it, maybe that’s not really it. Because you’ve been so caught up with yourself [ …] Maybe you don’t even let people in. So, I think it is really important to sit down and reflect on your relations a little. (Bereaved caregiver 4) Pg 4  **Caregiver (bereaved)** The relations are so complex that it is not so easy to put a number on them, or to indicate who cares about whom or how strong or weak [the relationship] is. / … / For me, it is more of a thought process of, well, what really was my relation to my brother and what worked well and what didn’t? And I can do that from his name on the map, but to put a number on it and value it as strong or weak? I would say that the relation to my brother is strong, but at the same time complicated. So, I think, maybe, this grading system / … / I don’t really see how much it would give me to put a number on my brother. (Bereaved caregiver 2) Pg 4  **Patient** Maybe, for some people, it would work from day one. Others might need some time before they feel that they are interested and so on. But it should be available, so that you know that it is there when you need it. (Person living with brain tumor 4) Pg 4 | X | |  |  | F86 |
|  | Self-care supportive relations | **Caregiver (bereaved)** And that you sometimes feel that you need help, but […] you don’t really know with what or in what way, everything is just a huge pile. I feel that it is hard to ask for help until I know what I want. (Bereaved caregiver 3) Pg 5  **Caregiver (bereaved)** I can only speak for myself […] but friends can say ‘You’ll fix this’ or ‘It’s going to be alright’. That really makes me mad! You have no idea what you are talking about, it’s not going to be okay! But when you meet someone who is in the same situation or has been, they know exactly, you don’t even have to explain. It’s like ‘Yes, I feel the same way’. So that support is really, really important, I think. (Bereaved caregiver 4) Pg 5  **Patient** I’m not worried that she will break. What worries me is that her physical and mental energy level will be so suppressed that she will  lose her joy of life along the way. It’s a lot about that. I find that part the hardest. (Person living with brain tumor 7) Pg 5  **Caregiver** People get divorced because they feel that they don’t get enough space. But as an informal caregiver you are expected to think that it’s okay, […] that I can’t live on my own terms […] have wishes, that it should be okay for me to live in a void. That feeling makes me irritated sometimes. No one told me that, but it’s still what is expected. (Informal caregiver 4) Pg 5-6    **Patient** Even if the professional competence is there, it’s not always just that you are looking for. Maybe you are also looking for a bit of understanding and the emotional bit. (Person living with brain tumor 7) Pg 6  **Caregiver** Their role in cancer care can’t be stressed enough. [ …] they helped us through so many of those steps. […] [and] they gave an immense sense of security. We could call and they gave us answers, they asked a bit about how you were doing, gave you the warm tap on the shoulder. It became a little bit of a family. […] Otherwise, you are pretty much left on your own. (Informal caregiver 4) Pg 6 | X | |  |  | F87 |
|  | Identity-preserving relations | **Caregiver** My job is extremely important. I can actually make a difference there, actually do something about things, unlike with mom’s illness, where I can’t do anything. […]. And the understanding [from the employer] is important. (Informal caregiver 5) Pg 6  **Caregiver (bereaved)** It was […] the only place where it was business as usual. At home somebody was missing, and other places reminded me that he was gone. But at work, there was … my tasks were the same, my colleagues were the same, nothing was missing there. (Bereaved caregiver 6) Pg 6  **Patient** Then there was six weeks of radiation therapy […] so I took a taxi […] to the hospital five days a week […] and walked […] to the sports centre, worked out a little and hung out and had coffee with the people there, because, you know, I knew them. […] Then there was six months of chemotherapy. Yes, the same thing all over again. (Person living with brain tumor 4) Pg 6  **Caregiver (bereaved)** I have some friends who say, ‘but you’re a little like this or that’. […] And then, it can be difficult to develop as an individual because that person knows that I used to be in a certain way and so I continue to be that way. It’s nicer with new connections, so that you can start over. (Bereaved caregiver 3) Pg 7  **Caregiver (bereaved)** It gives me something to get to know people I did not know before […]. It’s different for different people but for me […], sometimes I don’t want to be only with those who know my background, sometimes it can be extremely relaxing to hang out with people who didn’t know me before […] to not have that common story […]. (Bereaved caregiver 1) Pg 7 | X | |  |  | F88 |

| **Authors: Deatrick et al 2018** | | | |  |  |  |  |
| --- | --- | --- | --- | --- | --- | --- | --- |
| **Participants, Clinical and Demographic Characteristics** | **Findings** | **Illustrations (Page number)** | **Evidence** | | | |  |
|  |  |  | **Unequivocal** | | **Credible** | **Unsupported** | **Finding Number** |
| N=45 caregivers  N=28 males, N=17 female  Age 52.5 (SD 6.34)  All parents | Child’s daily life | **caregiver** Amber’s mother stated, “. . . Her IQ test came out average,which to me was fine after what the kid’s been through. And she had a lot of strengths.... Some of the weaknesses were nothing new to me. Processing slower. . . .” Also, her mother noted that although Amber has a college degree, she had significant assistance from college and family members. Pg 327  **caregiver** Nesta’s mother focused on vulnerabilities and differences from peers. For example, she said “… Food is 24/7.... He’s never been happy because it would mean he’d need to stop thinking about food... he has an outburst... it’s over and he doesn’t think about it” and “I accept he’s different but I’m not willing to accept that there’s not more for him.”  Pg 327 | X | |  |  | F89 |
|  | Condition management ability | **caregiver** Amber’s mother said that she felt competent to meet caregiving demands related to Amber’s special needs pointing to routines to help her daughter care for herself. “. . . At night I push things she needs (like medications) to the front of the counter because I don’t want her to forget. That way I can monitor how she is doing and not too intrusive.” Page 327  **caregiver** Although Nesta’s mother reported that “. . . the temper tantrums over food have gotten better as he’s gotten older,” overall, she did not see his condition as manageable in terms of her competency to carry out management now and into the future. “It’s just frustrating because there has to be an answer. I can’t find it.”Page 327 | X | |  |  | F90 |
|  | Condition management effort | **caregiver** Amber’s mother recognized the importance of the survivor taking more responsibility for condition management. However, she also reported that it was sometimes difficult to refrain from doing things for her, such as refilling prescriptions instead of encouraging Amber to call for renewals herself. “I know it is more work but I have to keep the long-term objective in mind.” Page 328  **caregiver** Nesta’s mother described not only his 24-hour, 7 days/week care that included locking anything in the kitchen with food in it but also the work of asking professionals questions, which they could not answer. She described constantly struggling with knowing how much to limit her child’s eating. “He needs 24/7 care; otherwise he will leave to get food.” Page 328 | X | |  |  | F91 |
|  | Family life difficulty | **caregiver** Family life was viewed positively, but difficulties were acknowledged. Amber’s mother stated, “So there were changes both ways; some we became better parents because of what we went through and then other things we had to work around.” Page 328  **caregiver** Mother reported that family life changed dramatically after Nesta’s diagnosis, with many aspects of everyday life becoming more difficult. The condition and its management had become the center of family life, especially for siblings. Siblings were described as both protective and resentful, “. . . It is very hard when you can’t get an adult to understand; [how can you] expect children (siblings) to understand?” Page 328 | X | |  |  | F92 |
|  | View of condition impact | **caregiver** Amber’s mother acknowledged the seriousness of the situation and implications for her daughter and family’s future. Although the health care team advised that it is unlikely Amber will live  independently, she continued to weigh evidence supporting and contradicting that probability. Meanwhile she focused on Amber being more independent, “I just want her to do something where she feels valuable and in control, and I’m not telling her she wants to go out and go find an apartment now but I’m sure at some point she’s gonna want to do that. . . .” Page 328  **caregiver** For example, Nesta’s mother addressed the seriousness of his condition and its profound implications for her son’s and family’s future. “The older he gets, we realize I am a full-time caregiver  probably for the rest of his life.... Where that does that leave us?” Page 328 | X | |  |  | F93 |
|  | Parental mutuality | **caregiver** She described her relationship with her husband as complementary in meeting challenges they faced. “If I was feelin’ like this, he happened to be feeling like [that]. . . . We never pulled each other down. . . .” Page 328  **caregiver** Nesta’s stepfather was sympathetic to the many caregiving challenges; however, his mother said that he had not fully understood them until spending more time at home because of a recent illness. “I said, “Well, that’s what I would tell you when you worked late.” Page 328 | X | |  |  | F94 |

| **Authors: Foust Winton et al 2021** | | | |  |  |  |  |
| --- | --- | --- | --- | --- | --- | --- | --- |
| **Participants, Clinical and Demographic Characteristics** | **Findings** | **Illustrations (Page number)** | **Evidence** | | | | |
|  |  |  | **Unequivocal** | | **Credible** | **Unsupported** | **Finding Number** |
| 27 participants interviewed  median age 58.5 years; range, 21 years –83 years.  Most participants were white (n=25), female (n=15) with n=1 African American participant. Length of hospital stay between 3 days – 13 days. Majority of participants had no prior pain history of history of opioid use.  Majority had an anterior craniotomy (n = 25) with sedation (n = 17).  The most common tumour types were glioma / glioblastoma /  Oligodendroglioma / oligodendroma and meningioma. Participants were diagnosed with all grades of tumours, with grade 1 being the most common. | Pain-as-nonsalient, routine pain management | **Patient**…wasn’t a piercing pain. It was more like a discomfort. (Patient, female, 76, grade III astrocytoma) Pg 175  **Patient** Well, basically…they [the staff] would ask me, how do you feel? What’s your pain level? And I would tell them…. And um, they would address that with medication. (patient, female, 76, grade III astrocytoma) Pg 175  **Patient** The pain has not been real terrible, not excruciating, so tolerable… I guess. (patient, male, 54, hemangioblastoma; grade not available) Pg 175  **Patient** I like the fact that they would let me talk and know if indeed I felt I needed something [for pain] or if I thought I could get through, they treated me as if I was intelligent. [I would say], “Let—let’s wait another hour until the meds kick in and then I can maybe sleep through the night.” Or whatever like that. So when they walked you through like that, I appreciated that. (patient, female, 66, grade I subependyoma)  Pg 175  **Patient** You just wait till it goes away. [You] just go over the hump and that’s it. (patient, female, 64, schwannoma; grade not available) Pg 175 | X | |  |  | F95 |
|  | Pain-as-salient, routine pain management | **Patient** The pain [the first day] was extremely excruciating — I cannot say the word. It was horrible. (patient, female, 48, grade I meningioma) Pg 176  **Patient** I definitely took the pain medication. (patient, male, 65, grade IV oligodendroma) Pg 176  **Patient** As soon as I woke up, it was like, “Oh, my, God, I’m in so much pain.” And they told me how much to rate it. And I said, “A 20.” And she said, “Okay.” She said, “We’re gonna give you something for it.” They didn’t let me sit there long before they took care of the situation and gave me something to take care of it. But yeah, when I first came out of it, it hurt like a mother. (patient, female, 48, grade I meningioma) Pg 176  **Patient** They’d come in 3 of 4 times a day [and] would give me Norco or Valium or both, depending on what I needed… They were pretty on top of keeping my pain in check, which was nice. (patient, male, 22, astrocytoma; grade not available) Pg 176  **Patient** So knowing that [the medication] lasts for 4 hours, 4 hours and 10 or 15 minutes is important, that way I can let these guys know like I did… because I know she’s gonna be busy  and that way she can get the pills and so forth… because I’m one of 4 or 5 people that she’s taking care of… so I definitely wanted to let her know that I was ready for it. (patient, male, 28, grade II glioma) Pg 176 | X | |  |  | F96 |
|  | Pain-as-salient, complex pain management | **Patient** Oh, yeah, I was crying, shaking, all the nine yards. (patient, female, data not available) Pg 176  **Patient** They were trying to give me Percocet, and that takes about an hour to kick in, and my pain, they had trouble staying on top of it for a while, so they gave me an IV that kicked in right away, and then some Percocet on top of that… (patient, male, 77, grade I hemangioblastoma) Pg 176  **Patient** I even think the blood pressure and all that can be very related to the pain, and my blood sugar has been up and down. I think a lot of it has to do with the pain. (patient, female, data not available) Pg 176  **Patient** After the morphine it [the pain] got so  much better…. But they were able to absolutely get me back on the fentanyl without it dropping my oxygen even more, and then they have been giving Percocet and Vicodin. So that was what kept me pretty much not in pain. (patient, female, data not available) Pg 176  **Patient** They [staff] just asked me how much pain I was in. I gave them a number, and they said, “What’s a tolerable—manageable pain for you?” I said, “If it’s about a 5. I’m good. If it starts to get up to a 6, we need to start the fentanyl because after 6, it starts to go up really quickly from there, so if I say it’s 6, fentanyl time…” But at one point, it wasn’t. Because I wasn’t getting the fentanyl every hour like I’m supposed to so, it would go back to, “Okay. We’ve got to get this every hour on the hour again.” And so it got to the point where I’d be like, “Give me the fentanyl before you start doing your charting because then it will be an hour before you can get back in here because I can’t deal with going through this again. (patient, female, 36, pseudomeningocele; grade not available) Pg 177  **Patient** The nurse was very nice to come in and she said, “Well, what can I do for you? Just tell me, what do you want me to do because I’ll do anything I can.” And that in itself was nice to hear, and she was able to get me a medication to calm me down a little bit. (patient, female, 61, grade I schwannoma) Pg 177  **Patient** They [the staff] did everything they can  to possibly help me…. They have been there for me, ‘What can I do to help you? (patient, female, 54, grade III oligodendroma) Pg 177 | X | |  |  | F97 |

| **Authors: Francis et al 2022** | | | |  |  |  |  |
| --- | --- | --- | --- | --- | --- | --- | --- |
| **Participants, Clinical and Demographic Characteristics** | **Findings** | **Illustrations (Page number)** | **Evidence** | | | | |
|  |  |  | **Unequivocal** | | **Credible** | **Unsupported** | **Finding Number** |
| N=10 spouse caregivers.  Seven women and three men who were providing care for a partner in treatment for primary malignant brain tumour (PMBT). Spouse caregivers ranged in age from 36-76 years. Two couples had children living at home and ouple had grown-up children no longer at home. | Enduring everyday life | **Caregiver** It is a fight to get all the different things to work together when there is no surplus energy left. It is even difficult to just make a meal. When I am like this, it makes me feel powerless, because this is the root to everything else, that the coordination of our home life is sailing around. (P1 – female) Pg 323  **Caregiver** I am nervous, I am 7 years older than her (the ill partner) and have had a heart operation. If something happened to me, everything would fall apart. It is important for us, that I keep reasonably fit. (P6 – male) Pg 324  **Caregiver** If I just throw in the reins and let go of  how it actually feels, then I am not sure I can get hold of myself again and keep it all together. In one way or another, it is easier to just keep it in. (P4 – female) Pg 324  **Caregiver** I was in the car on the road, and all of a sudden, everything just began to spin out of control. It just started spinning and I was really dizzy. I drove onto the slip road, opened the  door, and put one foot out, everything was just spinning. (P3 – male) Pg 324  **Caregiver** I read in different leaflets that you should look after yourself; otherwise, you cannot look after others. But how can I think of myself, how am I supposed to do that? (P1 – female) Pg 324 | X | |  |  | F98 |
|  | Being overlooked and hurting | **Caregiver** ‘Give us a hug,’ … it is going so fantastically well. Newsflash! It is not. There is a world outside the numbers of the thrombocytes. (P2 – female) Pg 324  **Caregiver** It is missing along the way, some good advice about what to do… You do not get that. Nobody tells you what to do or preparing one on what is going to happen. (P6 – male) Pg 324  **Caregiver** I could kick them (friends), when they say, ‘You are just like your old self.’ I think to myself that they do not even bother to notice how he is now and how he used to be. (P4 – female) Pg 324  **Caregiver** Then I get angry, then I do not need them to come, if it involves extra work for me. They have no idea of what I am doing, absolutely no idea, they are totally oblivious to it all. (P2 – female) Pg 324 | X | |  |  | F99 |
|  | Being acknowledged and feeling good | **Caregiver** My parents empty the dishwasher and do the vacuuming without me having to ask for  help, and that makes me so happy that I just want to cry. (P1 – female) Pg 325  **Caregiver** I have a good colleague and we often take a trip to the main street just for a cup of coffee. She is good to talk to… when I have something that I need to get off my chest. (P5 – female) Pg 325  **Caregiver** When one feel so affected by it all… it helps when something is happening. The evening I went to the theatre, it was great, a really good evening out. I was just like… it is such a long time ago since I have had such a good evening. (P10 – female) Pg 325 | X | |  |  | F100 |

| **Authors: Fraulob and Davies 2019** | | | |  |  |  |  |
| --- | --- | --- | --- | --- | --- | --- | --- |
| **Participants, Clinical and Demographic Characteristics** | **Findings** | **Illustrations (Page number)** | **Evidence** | | | |  |
|  |  |  | **Unequivocal** | | **Credible** | **Unsupported** | **Finding Number** |
| 84 qualitative comments  No demographic information reported. | Experience of Care and Support From General Practice | **Patient** “I have not had any follow up care or had any contact to them to check progress and wellbeing,” or “following my operation there was no communication from the GP regarding the surgery.” Page 316  **Patient** “GPs were insensitive and very unprofessional towards my family,” “GP care could be better‚” “GP care was very poor,” and “I am not happy with the level of service with my GP.” Page 316  **Patient** “More input from my GP and district nurses for side effects, seizures, swollen feet.” Page 316  **Patient** “It took a month to sort out diagnosis but my GP group practice remains supportive in every way,” Page 316 | X | |  |  | F101 |
|  | Experience of Overall Coordination in Care | **Patient** “more co-ordination care between GP and hospital and district nurses.” Page 319  **Patient** “No copies of my details yet from recent contact sent out” or “GP didn’t receive discharge letter as never posted from the N” or “no information was given to my GP—he had to write a letter asking for advice on future care.” Page 319  **Patient** A few patients described their GP receiving misleading information: for example, “stating my tumour was benign when in fact it was malign” or “not told enough about my side-effects and difficulty in needing to go to my GP” Page 316 | X | |  |  | F102 |

| **Authors: Gately et al 2020** | | | |  |  |  |  |
| --- | --- | --- | --- | --- | --- | --- | --- |
| **Participants, Clinical and Demographic Characteristics** | **Findings** | **Illustrations (Page number)** | **Evidence** | | | | |
|  |  |  | **Unequivocal** | | **Credible** | **Unsupported** | **Finding Number** |
| N=10 participants  Six survivors (4 male and 2 female); Four caregivers (3 spouses and one sister).  Survivors were aged between 35-78yrs (mean: 56yrs) and had a median overall survival of 7.3yrs (range: 2.4-8.8yrs). Two survivors had experienced recurrence with only one showing signs of recurrence and receiving active therapy at the time of interview. | Disconnection from past self *‘who I was’* | **Caregiver** He feels as if he has been cheated and the rug has been pulled out from under him. (Wife of P6) Pg 286  **Survivor** There are days when I think I should be out there working, doing what I love and I’m not… It’s taken away my career after 35 years. I can’t get that back. (P5 – male) Pg 286  **Survivor** The whole thing has forced me into retirement…when I thought I would carry on. (P6 – male) Pg 286  **Survivor** My arm – I can’t strum the same way on my guitar that I used to. (P4 – female) Pg 286  **Survivor** It was part of my self-directed rehab to start singing so I joined a choir. (P3 – male) Pg 286  **Survivor** I was one of the best jujitsu fighters in the state… I never went back…just don’t have that want to fight people anymore…doesn’t work for me. (P1 – male) Pg 286  **Survivor** There are times where I thought I was on the road to becoming a cripple…it’s hard. (P3 – male) Pg 286  **Survivor** I want someone to be there because I’m a bit scared. (P4 – female) Pg 286  **Caregiver** His left side is not good, and he now walks with a stick. He can’t cook and I cut up his food. (Wife of P5) Pg 286  **Caregiver** He used to be self-sufficient but now it’s changed…he [wants] to do everything with me…at the hip. (Wife of P6) Pg 286 | X | |  |  | F103 |
|  | Disconnection from present self *‘who I am’* | **Survivor** I look fine, but inside I am tormented...sitting around looking sick isn’t healthy for the family. (P5 – male) Pg 286  **Survivor** People are trying to be compassionate and nice but what happens is they help to remind you every day what’s going on and you’re actually trying to forget. (P5 – male) Pg 286  **Survivor** I don’t make really strong connections with people anymore. I’m a little bit agoraphobic –for the most part, I just don’t go out much... because of the stress of it. (P1 – male) Pg 286  **Caregiver** I do find that when people come around, he is always making excuses that it looks worse than it really is. (Wife of P5) Pg 287  **Caregiver** gaming the system. He thought survivors work, so I’ll work, and I’ll survive. (Wife of P3) Pg 287  **Caregiver** She is I think understandably depressed, understandably paranoid about every  little ache and pain. And so that prevents her from living life a lot of the time. (Sister of P4)  Pg 286  **Survivor** I quite often lock the door, chill out and put some music on and not answer the door for a couple of hours. (P5 – male) Pg 286  **Survivor** I don’t wake up every morning thinking this is what happened to me...I actually never think about it. (P1 – male) Pg 286  **Survivor** Obviously, I was angry and annoyed at what has happened, but you can’t waste your energy on that. I focus on the future. (P5 – male) Pg 286  **Survivor** Biggest coping mechanisms is I’ve been smoking weed. (P1 – male) Pg 287  **Caregiver** Drinks an awful lot...he says this is what I need to cope. (Wife of P6) Pg 287 | X | |  |  | F104 |
|  | Disconnection from future self *‘who I could be’* | **Survivor** thanking my lucky stars everyday...to hit two and a half years is sort of like well there is hope. (P5 – male) Pg 286  **Survivor** I don’t deserve to be so lucky. (P3 – male) Pg 286  **Caregiver** a little unreal. (Wife of P5) Pg 286  **Survivor** I don’t think about it. (P1 – male) Pg 286  **Survivor** I see myself becoming an artist with an eBay shop. (P4 – female) Pg 286  **Survivor** I’m thinking that probably my next big change is going to be retirement – probably in 8–9 years. (P3 – male) Pg 286  **Survivor** There has been a bit of reticence about planning too far in advance...but I made sure I took out travel insurance with a company who would cover me (laughs). (P6 – male) Pg 286  **Survivor** I just go along. (P2 – female) Pg 286 |  | | X |  | F105 |

| **Authors: Halkett 2010** | | | | |  |  |  |
| --- | --- | --- | --- | --- | --- | --- | --- |
| **Participants, Clinical and Demographic Characteristics** | **Findings** | **Illustrations (Page number)** | **Evidence** | | | | |
|  |  |  | **Unequivocal** | | **Credible** | **Unsupported** | **Finding number** |
| N=19 patients with HGG  55 years (range 31–74, SD = 10).  N=12 males and N=7 females | Feelings of uncertainty around prognosis and quality of life | **Patient** “We still don’t know what we’re going to have to look forward to in the future because, you know when you do ask you still get a little bit of a vague answer” page 115  **Patient** “like I want to know, would I have a stroke or would the symptoms just come back because the tumour was just growing back….you know I wanted to know what was going to happen to me, would I have a seizure, because I wanted to tell my children, what to do if they’re home alone with me and something happens” p115  **Patient** “If you think about what part of your body you don’t want surgery on its your brain, and you know that’s what I ended up having. The question is OK you come out of it, you’ve gotta have this operation you’ve got a surgeon that you know is a good surgeon, that’s great. But at the end of the day you find out what your new condition n is like when you wake up and, am I going to be able to speak, am I going to be able to move my arms and legs, am I going to be able to swallow, and I going to be able to do these things….?” p115 | X | |  |  | F106 |
|  | The need for individualised information | **Patient** ‘‘I never got really good confirmation of my condition. . . They thought it was the bad one; which I worked with in my own head until eventually and it’s quite right they’ve got to do it until they get confirmation it’s such a long winded thing. . . .over a week to get a biopsy confirmed. And a week is a long time to hover around – thinking. . . and that was after having all the, up to the operation, having the operation and then the thing gets sent away another week. After that you get told that you have got whatever it is and then, then you get the right information.’’ P115  **Patient** ‘‘I was just looking at trying to find something to read and I couldn’t find anything about brain cancer or anything like that.’’ P115  **Patient** ‘‘I’ve got a loss of little peripheral vision he thinks. . .’’p115  **Patient** ‘‘I’m not very good at reading. I want them to tell me.’’p115 | X | |  |  | F107 |
|  | Dependency on carer | **Patient** ‘‘They give you a steroid to shrink the tumour and you loosecontrol of your appetite so you just want to eat sort of 24 hours a day, you just feel like eating and so you have to control that otherwise you just blow up like a balloon and then they dump anti-nausea drugs on you so you can cope with the chemo and that slowed down my metabolism down again so I not only ate ,it just didn’t go anywhere.’’ P115  **Patient** ‘‘My husband does it all. . . That’s what I say to him, ‘‘Thank goodness I’m not doing all this, because, Oh God I could give myself anything.’’ It’s wonderful that he does it all for me.’’  **Patient** Yeah, I’ve got to sit here all day, listen to the radio and watch television, I can’t do anything at all (tearful mumbling), it’s affected all my balance, my nerves in my arms and my legs and I just can’t do anything, it’s very upsetting. . .’’ p116  **Patient** ‘‘You know we have got a driving issue. We are from the country and suddenly I cannot drive, and I have got a wife and who has only driven in a country town, driving in the main streets of the city and it is just chaotic. So, I’m having to try to say: ‘‘You do not do that’’ and we’ve driven home in tears and the pattern is almost everyday.’’p116 | X | |  |  | F108 |
|  | Communication with health professionals | **Patient** ‘‘I think I’ve been involved with it (decision-making) but at theend of the day it’s like, we’ve got this choice of doing this or not; like well at the end of the day it’s your decision. It’s one of those things where you do have a decision but at the end of the day you really don’t.’’p116  **Patient** “They had a family consultation which I knocked back and made an individual consultation and got down to the nitty gritty straight away and I wanted to know what it was all about. . . They told me what would happen eventually when the inevitable happens. They were real up front. . . The doctor spent nearly an hour and a half answering every question that I had.’’p116  **Patient** But it’s very frustrating when he can’t speak and use words, and he puts in all the wrong words. . .Very frustrating and he also finds that humiliating because it’s quite indecipherable when he does that.’’p116 | X | |  |  | F109 |
| **Authors: 1608 Hazen 2016** | | | |  |  |  |  |
| **Participants, Clinical and Demographic Characteristics** | **Findings** | **Illustrations (Page number)** | **Evidence** | | | | **Finding number** |
|  |  |  | **Unequivocal** | | **Credible** | **Unsupported** |  |
| N=7 patients, N=6 caregivers  Patients: n=4 female, n=3 male, age 52.86, range 42-66 years old  Caregivers: n=5 female, n=1 male, age 50.3, range 39-63 years old.  Relationships not reported. | Current Challenges | **Unclear participant** P06 explained this in saying “I think [knowing more about] the medications [and] the treatments would have been helpful because you feel like you are jumping out of an airplane without a parachute when you start this journey.”p1807  **Unclear participant** For others, the amount and presentation of information was overwhelming, as described by P06 in saying “It’s actually kind of hard from my viewpoint, going through this. I think they are talking at you a lot, and we’re both pretty much in a little state of denial or something… You can’t keep track of all the information they are giving you and be able to register it enough to keep everything in your head.”p1808  **Unclear participant** Interestingly, C06, the other half of this patient-caregiver dyad, countered this, expressing frustrations over missing information several times, and summarizing in saying “Well, I would say that is true for you. I could keep track of everything they said because they didn’t tell me very much [both laughing]. I felt a lack of information, and you were overwhelmed by everything because of your state.”p1807  **Unclear participant** This scenario was described by P04 in saying “Well I got the distinct impression that he was trying to invoke the power of positive thinking. He didn’t want to put any negative sort of doom-saying scenarios into the works because that can probably turn into a self-fulfilling prophecy.”p1807  **Unclear participant** “What most of the doctors say is like… ‘oh well this is your tumor, and there is no other tumor like it. So your experience is your experience, and there’s no such thing as an average.’ And so they make these projections as to how I might or might not respond, but they don’t know, and they always quantify it saying ‘I can’t tell you because it’s you and your tumor, and it’s not somebody with their tumor that’s had the experience that’s in the statistics.’ And so the trouble with that is you come away without any knowledge whatsoever…”  P1807 | X | |  |  | **F110** |
|  | Current Behaviours | No evidence quote for this subtheme just discussion |  | |  | X | NS8 |
|  | Future Behaviours and motivations | **Unclear participant** Some participants like P07 felt that this would be a good way to contribute, saying “I said right from the beginning, I would be happy to help down the road… I am not the last one that’s going to get this diagnosis, there’s people coming up all the time with it. If I can help somebody else, I would be happy to do that.” P 1811 | X | |  |  | F111 |

| **Authors: Heckle et al. 2018** | | | |  |  |  |  |
| --- | --- | --- | --- | --- | --- | --- | --- |
| **Participants, Clinical and Demographic Characteristics** | **Findings themes** | **Illustrations (Page number)** | **Evidence** | | | | |
|  |  |  | **Unequivocal** | | **Credible** | **Unsupported** | **Finding number** |
| 17 caregivers of PMBT  Age 65.3 years (SD9.9), female 70.6%, spouse 58.8%, child 41.2% | Assessment of the situation | **caregiver** “and then my husband started to have hallucinations.” (H10) p196  **caregiver** “I wouldn’t have thought, not for a second, that he would die.” (T12) P196  **caregiver** “Well there are some diagnoses where the first impression is already: death sentence.” (H11)  P196  **caregiver** “and then you ask yourself, now what do we do.” (H15) “even though I informed myself about hospices, because I realized that this won’t last any longer, and it would not help my wife if I collapsed.” (H2) p196 | X | |  |  | F112 |
|  | Dealing with the situation | **caregiver** “the biggest burden has been to organize the assignments who drives to [place D]” (T9) p196  **caregiver** “and the dying itself wasn’t at all dreadful. Rather I felt a great peace, with the dying at home” (H7)  P196  **caregiver** “but, as I said, it’s always difficult, because you do not want to burden the other one, you talk about it, but still, something you rather keep to yourself, because you do not want to burden the others, and that’s how everybody feels.” (H1) p196 | X | |  |  | F113 |
|  | Effects of the situation | **caregiver** “we put the bed, exactly. We got the adjustable nursing bed. We cleared the living room and put the bed there, because only there we had enough room. And then we, because it’s quite roomy, the living room, we put the nursing care bed in one corner und in the other we had a couch, on which we slept in turns at night.” (H9) p196  **caregiver** “well, in the evening I did, during the day, early in the morning I did not wash him, I said, I just cannot manage it, because I have to be at the office at 6.30 am, I got up at 5.45 am or even 4.45 am.” (T4) p196  **caregiver** “Due to the psychological burden I was on sick leave.” (T1) p196 | X | |  |  | F114 |
|  | Support by others | **caregiver** “Yes, so, the social environment which I, so the problem which I had, to my good luck, I can thank my social environment, the problem vanished. They are of the kind, that when they come in and see a problem, in the next moment it’s gone. That’s what I find so cool.“ (T2)p 196  **caregiver** “I mean, these are very personal things, who should step in. “and “because in this situation, nobody can help you.” (H4)  p 196  **caregiver** “would have wished that I could have gone someplace and could have talked to somebody.” and “the bureaucracy, which you have to comply with. That got to my nerves, […], that would have been a real help for me.” (H5) p196 | X | |  |  | F115 |
|  | Information | **caregiver**: “[…] so that, when all is said [in the first contact between professional and informal caregiver], we could set up another appointment, if you find you have more questions, we could give more explanations“ (BT16).p197 | X | |  |  | F116 |
|  | Perception by others | **caregiver** “In hindsight my sister-in-law once said to me, I have now been in there [with the patient], we went home, I was all run down. And, she says, I now can understand you when you say, you are run down. I could not have stayed in there over night, I could not (BT15)”p197 | X | |  |  | F117 |

| **Authors: Hricik et al. 2011** | | | |  |  |  |  |
| --- | --- | --- | --- | --- | --- | --- | --- |
| **Participants, Clinical and Demographic Characteristics** | **Findings themes** | **Illustrations (Page number)** | **Evidence** | | | | |
|  |  |  | **Unequivocal** | | **Credible** | **Unsupported** | **Finding number** |
| 18 caregivers of patients with PMBT  Female 80%, 50% spouse, age 21-63 years mean not reported. | Patient Changes: The new normal | **caregiver** “I care for the kids 24/7. I’ve taken on a lot of the chores around the house that include lifting, taking out the garbage, cleaning the house, and I’ve taken care of the bills and dealing with the insurance company, and making sure he gets the medication filled.” P 5  **caregiver** “[The patient] said, “I turned suddenly and have a headache.” I got cold. I thought this is how the whole thing started. I thought, “He’s recurred.” I kept watching him and checking him: “How’s your head now?” I think this will probably continue forever. I think I’ll probably always be afraid of recurrence.” P 6  **caregiver** “I’m a nurse and there’s a lot of things that I understand more than someone else would. But it’s still your kid and it’s still scary…. I think that I’ll always have the concerns that I have about recurrence. I feel that’s my private concern and I have to show my “I’m OK” face to most people.” p 6  **caregiver** “What makes it difficult for her is having the memory problems; she doesn’t know that she can’t walk, so she gets upset that there is a seat belt on her wheelchair. Not knowing the different things that are going on with her, what’s causing them, and how to treat them.” P 6 | X | |  |  | F118 |
|  | Care giver Adjustments | **caregiver** “I’d say definitely closer than before. We definitely cherish each other more and tell each other that we love each other.” P6  **caregiver** “I worry about him more and he doesn’t like that. When we go out to eat he has trouble if he has to calculate the tip. He has trouble writing it—he has a tremor— and so I have to write it. I’m the one who keeps track of the medications, goes to the bank, goes to the post office; I’m the one that does the things that he used to be able to do.” P 6 | X | |  |  | F119 |
|  | Accessing Support | **caregiver** “What I didn’t expect is my mother-in-law and my sister-in-law being such wrecks. I would like them to show a little more interest in him. He has always shown interest in them. They should stop being so self-centered. Give him some peace at the end of his life.” P 7  **caregiver** “It would be nice if they’d call up and just offer to come sit with him for a day or even a couple of hours so I could take a break. Or even someone putting a meal together and dropping it off at your house. I would like people to take initiative and say, “Hey, Saturday I’m going to come and mow your yard.”p7  **caregiver** “Just talking to other people who are going through the same things that I am. Just being able to talk to them and knowing that I’m not going crazy, and that they’re going through it too, and how they cope. It has really helped a lot, just having people that know what you’re going through.” P7 | X | |  |  | F120 |

| **Authors: 867 Langbecker 2017** | | | |  |  |  |  |
| --- | --- | --- | --- | --- | --- | --- | --- |
| **Participants, Clinical and Demographic Characteristics** | **Findings themes** | **Illustrations (Page number)** | **Evidence** | | | | |
|  |  |  | **Unequivocal** | | **Credible** | **Unsupported** | **Finding number** |
| 19 patients, mixture of malignant and benign brain cancer. Female n=10  Unclear if the quotes are from benign or malignant cancer patients. | Theme 1: “Don’t need help” – a perception that support services were not needed | **Patient** “I was pretty negative about getting any help – I don’t know why. I didn’t see why I needed to go to these speech therapists. But getting better I- I realised that there was something wrong with my speech but I didn’t feel it at the time. (P2; 9 unmet needs at baseline, 1 unmet need at follow-up, 3 types of services used)” p1746 | X | |  |  | F121 |
|  | Theme 2: “Don’t want help” – a desire not to use support services to address needs | **Patient** “You tend to go with ‘oh well, if- if this is as good as it is then that's okay, I’ll just live with this’ . . . I never feel normal. (P34; 15 unmet needs at baseline, 9 unmet needs at follow-up, 6 types of services used)” p 1747 | X | |  |  | F122 |
|  | Theme 3: “Can’t get help” – difficulties accessing support services to meet the needs experienced | **Patient**.” . . at first off you’ve got to know, I mean- what I found this massive frustration part of it is what I’m experiencing is that normal part of, of having a brain tumor or is it a part of having had surgery, or is it just the side effects that I’m experiencing, how normal were they. (P17; 10 unmet needs at baseline, 19 unmet needs at follow-up, 7 types of services used)” p 1747 | X | |  |  | F123 |

| **Authors: 824 Mc Conigley 2010** | | | |  |  |  |  |
| --- | --- | --- | --- | --- | --- | --- | --- |
| **Participants, Clinical and Demographic Characteristics** | **Findings themes** | **Illustrations (Page number)** | **Evidence** | | | | |
|  |  |  | **Unequivocal** | | **Credible** | **Unsupported** | **Finding Number** |
| 21 caregivers, female n=17, male n=4, age range 30-70+ years (mean not reported).  N=20 spouse and n=1 parent. | A time of rapid change | **caregiver** “... She [the doctor] said ‘we’ve got the results of the test on the tumour’ and she said ‘it’s aggressive brain tumour’, and she put her fingers she said, ‘one, two, three, it’s in the fourth stage’ and she said ‘that’s not very good’ and she said ‘we’ll give you between six to twelve months’. I was stunned, shocked (C018).” P 474-475  **caregiver** “Oh no, it was really hard, you know emotional and highlights the fact that you haven’t prepared for anything like that in your life. I was only 38 or 39 and you don’t sort of think about dying (C003)” p 475  **caregiver** “The social worker said, ‘You know, there’s got to be someone that’s got to look after him’ and being the wife, I said ‘I have to do it don’t I?’ and I did (C018).” P475  **caregiver** “Well I did not go back to work fulltime, I was doing ten hours a week, you know. So the rest of the days I was home with him (C018).” P 475  **caregiver** “I haven’t ever driven in the city before ... ‘cause my husband always had driven and it was a big thing to take off and drive down there because he wasn’t able to drive (C020).” P 475  **caregiver** “You have to think about what we do now is different than what we would’ve done before. Whether we have more children, and we both wanted to have lots of children. Now I’m thinking it might not be a wise thing to do (C007).” P 475-476  **caregiver** “I needed more the practical stuff, what do we do? What should we expect? When we went to see the neurosurgeon, he gave us a very short time span and was talking about bringing [our son’s] wedding forward, like the wedding was in six weeks and that was really quite scary (C008).” P 476 | X | |  |  | F124 |
|  | Renegotiating relationships | **caregiver** “I think things change dramatically and you’ve gotta do everything that normally your wife used to do but we’re getting there (C001).” P476  **caregiver** “Everything was partnership and now it’s not and it’s very hard to get used to (C021).” P476  **caregiver** “I need to make the decisions myself as far as the doctor goes, I’m the head caregiver (C001).” P476  **caregiver** “You have to make a decision whether you’re going to extend life or extend death, oh no, prolong life or prolong death (C008).” P 476  **caregiver** “Too many. Too many because then you’re left wishing in your heart have I made the right decision, am I doing the right thing (C009).” P 476  **caregiver** Cause my husband really can’t make himself understoodat times and other times he can. But it’s very frustrating when he can’t speak and use words, and he puts in all the wrong words (C024). Pg 476 | X | |  |  | F125 |
|  | Learning to be a caregiver | **caregiver** “But it’s a big learning curve and it happened so quickly and that’s the thing which I found was hard and though people kept trying to help we couldn’t get the information that was really needed. You had to learn as you go along (C001).” P476  **caregiver** “The thing about it is that the information ... the abstract of not knowing where you’re going, what’s happening ... most of the time ... and that’s the thing that I find a bit confronting sometimes (C001). “ p 476  **caregiver** “Well I’ve had to shower her... When she first came out of hospital she couldn’t move much. She was paralysed down the left hand side so it was very difficult. I had to get her into a commode type chair, put her into the bathroom, get her from the commode to the sliding down chair (C001).” P476-477  **caregiver** And also the responsibility of being a watchful eye with him because even like the medicine I have to measure it all out for him. If you put it beside his bedside cabinet and put it there for after the meal it doesn’t mean to say he’s going to take it (C024) Pg 477  **caregiver** When I’m away I worry about what’s going on with him. I won’t let him go around the block without his mobile and I ring him to check that he’s alright ... Yes and I’m frightened of that for myself too, because I don’t know that I’m going to do the right things (C009) Pg 477  **caregiver** I can’t think of an activity that suits him. I mean apart from maybe taking him to the pool and keep him in the shallow part, you know, in case something goes wrong. It’s hard to find something that he can do (C003). Pg 477  **caregiver** “Yeah and then you neglect yourself too, like you don’t know all those things that you should do. Like go to doctors yourself (C003).” P477  **caregiver** “We’ve got good friends too that have had cancer in their family too. They are really great they come and you know, the couple of times he has had seizures my daughter has come over and they’ve come straight over (C003).” P477  **caregiver** “I don’t actually call out for help as much as I should. It would probably do me good to actually have a break where I can have [a friend] or someone come and be with [husband] for the day and maybe go and buy some clothes or something. ‘Cause I take him everywhere with me for that fact that he’s a seizure risk and that two times he’s hit his head and bled (C024). Pg 477 | X | |  |  | F126 |

| **Authors**:  **788 Molassiotis et al 2010** | | | |  |  |  |  |
| --- | --- | --- | --- | --- | --- | --- | --- |
| **Participants, Clinical and Demographic Characteristics** | **Findings** | **Illustrations (Page number)** | **Evidence** | | | | |
|  |  |  | **Unequivocal** | | **Credible** | **Unsupported** | **Finding Number** |
|  | Tiredness | **Patient***.* You just feel drained, it's just that you haven't got any energy at all and it's like it's been sucked out of you.”  **Patient***.* “Yeah, towards the end of the day and my mind's wandering, and then, as soon as I get home I'm absolutely drained”  **Patient***.* “when I get to the top of the stairs and I'm knackered.”  **Patient***.* “I feel like, sometimes, especially when I'm playing golf, I feel like an old man because I'm the only one there with a buggy and, in fact, I'm the youngest.” p 412 | X | |  |  | F127 |
|  | Neuro-cognitive symptom | **Patient***.* “I have to write everything down at work otherwise it won't get done, if I don't write it down it don't get done, it doesn't even enter my mind.”  **Patient***.* “If I went shopping and there was probably more than four or five items, if I didn't write it down I'd forget something, I couldn't remember. But I think now, I think it was a good job that I didn't get it earlier in life because the reason I always had good marks at university I was thinking was because I had a photographic memory when I was doing exams. And I don't think I have that now” p412 | X | |  |  | F128 |
|  | Social restrictions | **Patient***.* “Driving the most bothersome issue over everything else, it hurts” BRP12 at 1st interview “No, you can't drive. It's a bit of a kick isn't it?”  **Patient***.* “The only thing that hurts is not being able to drive e that really. been like 55 years with a car. Never been without a car in that time. And then all of a sudden somebody takes it off you or doesn't let you drive. You can't go to the shops for the whole week's shopping. You can't carry it. Whereas before we'd go down to the shop, do the shopping, stick it all in the boot of the car, jump in. I can see the sense behind it, but I don't agree with it” p412/413 | X | |  |  | F129 |
|  | Renewed perspective in life as a result of heightened awareness of mortality | **Patient***.* “ very stable, very stable, very, very stable indeed erm yeah, couldn't be better. Totally balanced and.. it's strange because I'm now taking an interest in the arrangement of things”    **Patient***.* “So it's a changing person if you like, so every time I go out for a walk I think oh I'm here, you know even the simplest things in life you think this is great.”  **Patient***.* “I felt, I woke up in the morning and I thought what am I doing with the day, you know, I've no, nothing planned to work towards and that worried me. But now I feel like I've got lots of things, you know. I've started doing some knitting .” p413 | X | |  |  | F130 |
|  | Fatalism | **Patient***.* “ So you sort of submit to it in a way” “Plus I can't do anything about it.  “.there's nothing I can do about it, well why worry about it” p413 | X | |  |  | F131 |
|  | Social contacts | **Patient***.*Participants attempted to maintain an independent life personality changes over time, restlessness and irritation were present p413 |  | |  | X | NS9 |
|  | Physical exercise & environmental changes | heightened awareness of the need to psychologically and physically readjust to the diagnosis was noted. Three (3/9) of the participants sampled added walking to their daily life and found an improvement in their psychological and contentment levels. Other participants had a more formal exercise regimen p413 |  | |  | X | NS10 |
|  | Complementary therapies | **Patient** “ Well I can remember on one of these panic modes when I was frightened looking down the .. hall there and thinking .. there's the toilet, you've got to get there and that seemed like a really hard task to find, to go to the toilet, you know. .. But then just at that moment I thought there's only one way forward and that's with Jesus. There's no other way at all. And from then on that's kept me sustained.” BRP20 at 2nd interview “  **Patient** “it is, it is a lovely experience, there's no doubt about that and I'm thinking, ‘right, ok, what I need to be doing now is getting as much positive, powerful energy in there, the lightness, the brightness which will then kill off, you know, these bits of glioblastoma that are still floating about there.’ So, yes, I suppose yeah, that spiritual element I believe that that part of it, positive thinking, will help you know, well it can't do any harm, and I'm enjoying it at the same time” [A patient describing his experience of transcendental meditation]. p414 |  | | X |  | F132 |
|  | Expectations | **Patient** Some cases of anger and dissatisfaction with the way the diagnosis was delivered to them by health care professionals. Also, it was noted in two (out of 6, 2nd interview) of the participants that there was a disparity between the symptoms expected by the participant and the symptoms actually experienced, particularly with regards to the symptoms related with fatigue, leading to anger. Communication problems and a perceived lack of sensitivity were also described. ‘‘It was the six week check and he [the doctor], he came in and he didn't even ask me how I was to be honest, he just started going about [the chemotherapy treatment]”. p414 | X | |  |  | F133 |

| **Authors: 616 Nixon et al 2010** | | | |  |  |  |  |
| --- | --- | --- | --- | --- | --- | --- | --- |
| **Participants, Clinical and Demographic Characteristics** | **Findings** | **Illustrations (Page number)** | **Evidence** | | | | |
|  |  |  | **Unequivocal** | | **Credible** | **Unsupported** | **Finding Number** |
|  | Family support | **Patient** My main concern was for my family how they would cope without me’  **Patient** ‘Was so important for my wife to come daily’  ‘I needed my family with me’ p 2263 | X | |  |  | F134 |
|  | Emotional support | **Patient** ‘Needed someone to share my emotions’ p 2263 | X | |  |  | F135 |
|  | Need for connection/loneliness/ depression | **Patient** ‘Post operation I was down as I couldn’t think what the correct words were and struggled explaining test phrases.’ p2263 | X | |  |  | F136 |
|  | No spiritual needs | **Patient** ‘I had no requirement for spiritual assistance’ p 2263 | X | |  |  | F137 |
|  | Religious needs | **Patient** ‘I really needed someone to pray with me I think’ ‘Whole thing has awakened in me stirred in me religious beliefs. Whole reappraisal of life.’ p 2263 | X | |  |  | F138 |
|  | Need to talk | ‘**Patient** Thinking about death and having no-one to talk to about it’  ‘Needed someone to talk to’  **Patient** ‘I needed to cry and be allowed to talk about my fears of not seeing my grandchildren ever, of not seeing my sons ever married or settled down. My fear relating to my son who has depression and what might happen to him. My sadness at leaving my husband after 32 years of marriage. I felt I was being hushed when I tried to say these things and that made me more upset. I needed to cry and say them.’  **Patient** ‘Asking a lot of questions p2263 | X | |  |  | F139 |
|  | Reassurance | **Patient** ‘Needed reassurances – about survival, to deal with inner panic and disappointment and fears’ p2263 | X | |  |  | F140 |
|  | Solitude | **Patient** ‘Just needed to accept this on my own’ p2263 | X | |  |  | F141 |
|  | Plans for the future/sense of normality | **Patient** ‘A scale of targets to aim for’  ‘I just wanted to get on with getting it sorted out, not worrying other people too much, and to get back to work and normality’ p2263 | X | |  |  | F142 |
|  | Plans for the future/sense of normality | **Patient** ‘I felt scared and thought I was probably going to die. This made me think seriously about my life, what had I done to deserve this?’  ‘I felt guilty that I had not done enough in my life’ p2263 | X | |  |  | F143 |

| **Authors: 548 Ownsworth et al 2015** | | | |  |  |  |  |
| --- | --- | --- | --- | --- | --- | --- | --- |
| **Participants, Clinical and Demographic Characteristics** | **Findings** | **Illustrations (Page number)** | **Evidence** | | | | |
|  |  |  | **Unequivocal** | | **Credible** | **Unsupported** | **Finding Number** |
|  | Intertwined and distinct caregiver support needs | **Caregiver:** For example, when Michael was asked if friends were supportive of him and his wife he replied: “Yes, which are basically the same thing.” These caregivers often used phrases like “when we were diagnosed” or **Caregiver** “when we went through treatment,” highlighting their shared experiences of both the brain tumour and support.  Other caregivers reported feeling supported if their loved one was supported. Laura (mother): The oncologist wasn’t personally supportive of me, I didn’t expect that, but by the fact that I knew she was so wonderful for my daughter that supported me.  **Caregiver** Not really for myself – more for Lucy . . . for other people to support her.  Caregivers perceived that access to information would have helped them to adjust to their caregiver role. William (father): Even if we had been aware of the support group and all the information available. . . that could have made our lives so much easier | X | |  |  | F144 |
|  | Varied expectations of support. | **Caregiver** When asked about support following her daughter’s discharge from hospital, Joanne noted: We never had any call back from them (hospital). . . or a call at home to see if we got there, nothing p7,8  **Caregiver** Other caregivers perceived that ongoing support even on a less frequent basis was supportive. Michael (husband): Well, (Hospital) you know there was support there all the time. . .. Even now when we go in we still meet some of them. Professionals were viewed as supportive when support was available as needed. For example, in discussing their GP, Sam (husband) noted: He wasn’t a daily source off support, but when we had to go and talk to him he was excellent. p8  **Caregiver** My parents have been there. . . but they’ve been more financial support when we really needed it, not emotional p8  I don’t think we got very much support at all from anywhere.  *HP support*  **Caregiver** When discussing his wife’s neurosurgeon Sam expressed: His manner’s been very encouraging and very supportive and I would classify him as being a source of support p8    **Caregiver** Doctors with a kind and caring manner were perceived as providing emotional support even when giving bad news. Laura (mother): She (neurosurgeon) had to give us some bad news some of the time. . . and you couldn’t ask for a better manner in her delivery of that bad news, or her support in what we were going through. p8  negative experience: There was no hello, we walked into the room and he (neurosurgeon) looked up from his desk and said you’ve got a very large brain tumour and it is an eight hour operation. Laura (mother): (We asked) do you think she will live? and he very tersely told us well, you want to be grateful that we’re not dead now . . . from our point of view all we really wanted was a little bit of reassurance. p8  **Caregiver** expected professionals and services to extend offers of support : Nobody ever rang up and said oh your daughter’s got a brain tumour, how can I help you? You know I’m from the hospital what can I do? p8 | X | |  |  | F145 |
|  | Factors influencing expectations of support. | **Caregiver** Caregivers advised that in the early stages following diagnosis they did not expect to receive nor seek support as they were more focused on treatment for their family member. Sam (husband): That was a time I guess of great shock in terms of support no, you’re basically just dealing with the issue p8  **Caregiver** would have liked to receive more information about brain tumor once the initial shock had subsided. Sam (husband): I guess we just wish that someone would have said to us right at the beginning here’s a very good guide, because when you have a brain tumor situation, oh you’re lost. Susan (wife) noted: I think that’s the time when some sort of support would be very helpful perhaps to a lot of families p8  **Caregiver** Practical issues such as time and distance and expectations about prognosis impacted caregivers’ expectations of support: could have done with something myself but I was pretty busy working p9 | X | |  |  | F146 |
|  | Strengthened | **Caregiver** I think it has made us closer. . . I’m a lot more tuned into him than I was before p9  **Caregiver** We pulled together for the family because we’ve always lived away from our families p9  **Caregiver** I think things like that have happened with Sarah and me; we’ve grown very close together as soul mates p9 | X | |  |  | F147 |
|  | Maintained | **Caregiver** We did then and still have a close relationship p9 | X | |  |  | F148 |
|  | Strained | **Caregiver** I’ve had to grieve for the man I married even though I’ve still got him . . .. It’s hard because some days John is really almost like the old John and you could sort of, do you say something to him or not? Yeah that’s hard p9  **Caregiver** Change in role: I did not really have too much to do with kids. I was riding dirt bikes and having a good time out there and sort of being single, to looking after Lucy and having bubs and the whole tumour ordeal | X | |  |  | F149 |

| **Authors: 490 Philip et al 2014** | | | |  |  |  |  |
| --- | --- | --- | --- | --- | --- | --- | --- |
| **Participants, Clinical and Demographic Characteristics** | **Findings** | **Illustrations (Page number)** | **Evidence** | | | | |
|  |  |  | **Unequivocal** | | **Credible** | **Unsupported** | **Finding Number** |
|  | Loss of control over physical and mental functions challenges patient’s sense of self | **Patient:** What’s it like, life on your back? It’s not nice. Everyday it gets progressively worse, it sort of creeps. Each morning you wake up with a little bit extra and it’s as if overnight, something goes into your brain and starts to scratch little bits away.”  **Patient:** “You know you’re not yourself because you’ve got no memory.”  **Patient:** “I find I get quite tongue tied sometimes but I never know if I’m tongue tied because I’m not speaking to yourself.” p 392 | X | |  |  | F150 |
|  | Difficulty grappling with increasing deterioration, disability, and dependence on others | **Patient:** “One of the things that strike me is how difficult it is to come to terms with feeling incapacitated. Going from an able bodied person, to suddenly having someone cut up your meal and feed you or shower you – that’s very hard to come to terms with.”  **Patient:** “Giving up my independence, that was the worst part of it.”  **Patient:** “I don’t want it to get to the stage where we have to move house because of my condition – you shouldn’t move because of that…I’ll never accept it, as simple as that.” P 392 | X | |  |  | F151 |
|  | Fear of being a burden to others Fear of burdening family, friends, and health care professionals with caring duties | **Patient:** “You’ve got to be careful when you press the buzzer, because the nurses – I know it is unfair to say it – but if you’re too demanding, they start to ignore you.”  **Patient:** “Going to a care place is better than, you know, expecting your friends to have to have look after you, you know, the chore of doing it.”  **Patient:** “Look, this is unbearable, you know? My partner has to stop work and drive me. It’s totally interfering with her work.” P 392 | X | |  |  | F152 |
|  | Need for someone to advocate on their behalf because patients felt unable to communicate their needs to others | **Patient:** “There were questions about if I could really stay at home. But it was really difficult to communicate that, and I really needed a third party to do that. The emotional stuff is the hardest stuff to deal with– the emotional fatigue from anticipating and thinking about the effect this is having on other people.”  **Patient:** “I’ve just got to the point where I say to [my carer], ‘you manage it,’ because I’ve lost a bit of my thought, and I’ve lost a bit of my speech.” “Someone came to my home to help. But she just sat there. And I was thinking, ‘Aren’t you supposed to be doing something? Like, give me a shower?’ Yeah, but I was only thinking that. I wasn’t saying that.” p392 | X | |  |  | F153 |
|  | Changed relationships with loved ones, as people fail to understand limitations | **Patient:** “People look at me and can’t tell any difference, I look normal, even now. And yet I can hardly see, and can hardly walk. Like even my brother, I’ve only seen him twice. He doesn’t even stand in the right spot where I can see him. He doesn’t even know where my vision is.”  **Patient:** “My partner visits [the hospice] a little bit. Well, I don’t really see a lot of him, but anyway, that has to be his decision, and I don’t know it’s hard.” p392 | X | |  |  | F154 |
|  | Physical limitations inhibit social being and exacerbated isolation | **Patient:** “The real company is [the sound of] the bed - as it shuffles you one way, and then shuffles you back. It’s actually a bit of company because you hear it let the air in and out…it’s an extreme sound, but you know it’s there, so it’s a comforting sound. It’s something that’s actually happening, rather than just silence and nothing else.”  **Patient:** “I can sometimes feel like an alien, alone, depressed. And it’s even worse because I can’t get out and I can’t drive, having to rely on my dad all the time.” p392 | X | |  |  | F155 |
|  | Patients coped day by day with limited preparedness for what the future might hold | **Patient:** “I’m taking it step by step, because I feel well. I can’t imagine that it would be such a sudden onset that I would just go down really quickly. I can’t imagine that.”  **Patient:** “I’m just going with the flow. But I think, ‘Oh, how long is this going to go for? Will I get to the six months period and then it declines?’” p394 | X | |  |  | F156 |
|  | Limited insight into care needs and “system” concerns, rather focus on everyday symptoms and limitations | **Patient:** “The more dex you take, the weaker you get. And you get very depressed, very quickly, because dex takes you down very low, plays with your emotions…All of a sudden you’re watching what you’re eating and all you’ve really got left, when you have a GBM, is that sort of small pleasure, like eating.”  **Patient:** “Fatigue is the biggest thing… but you can live with that.”  **Patient:** “I’ve got five brain tumours…but I don’t really notice too much, only thing I remember is tiredness” p394 | X | |  |  | F157 |
|  | Perceived health care professionals focus on the here and now Narrow focus of care provided, with no time given to existential concerns | **Patient:** You get caught up in appointments, blood tests, urine tests and things like that. Sometimes you just need someone to say, ‘OK, we’re doing really good, the outcome so far is good, but you know, what about you, what’s on your bucket list?’ It’s just a trigger for people think this is a death sentence. So what am I going to do with my time? Where do I want to be in six months?”  **Patient:** “I find doctors just don’t like it when you start asking them questions. But people need options, to have a choice for their self.”  **Patient:** “I’d be very happy to have an opportunity to talk with someone about what I’m going through and what it all means.” p394 | X | |  |  | F158 |
|  | Disappointments and their implications later perceived as lack of openness from health care professionals | **Patient:** “The danger of being paralysed by the biopsy would’ve been something I would’ve liked to have known…big time. But he [the surgeon] sort of brushed over it. He said there may be a bit of paralysis on my left hand side which would last for about three weeks. But it’s just a little bit longer than that, and a little bit more than minor.”  **Patient:** “[The surgeon] said, ‘I operated on you in the theatre…We didn’t get it all out.’ That was really heart wrenching. I thought, ‘What is he talking about, he didn’t take it all out?’ And I could have asked him, but I wouldn’t have gotten the questions that I needed to be answered. Because they like to protect you and make sure you don’t know.” p394 | X | |  |  | F159 |
|  | Provision of information about palliative care felt to be guarded. Those patients further along the illness trajectory felt it was inevitable and could have been helpful sooner | **Patient:** “Steer people towards palliative care. They must have palliative care, even though they’re scared of the meaning of the word, they will get the assistance they need. ‘OK, I’ve got a GBM, how long have I got?’ They won’t tell you that, they avoid the answer. But I think over a period of time you work out your own answers.”  **Patient:** “Refer to palliative care at the earliest possible time I think. I don’t know, soon after surgery? It would’ve been helpful then, having that support could help you manage.”  **Patient:** “We had to ask about it [palliative care]. We don’t want to be morbid, but we just want to plan. It’s about setting your life up for what it should be, so that the time we’ve got is our time.”  **Patient:** “I would have no idea what palliative care has to offer. How could I know?” p394 | X | |  |  | F160 |
|  | Sense of always waiting, from the time of diagnosis, right up to the time death | **Patient:** “At this stage there’s no decision about whether to go ahead with treatment, so this [the hospice] is basically just a nice, comfortable, pleasant place to be while waiting and seeing what’s going on, while waiting for their [the doctors] decisions about further treatment.”  **Patient:** “I feel like I’m just sort of waiting and so I’m a bit anxious, and I’m depressed. I’m anchored here, dependent on everyone else…so I feel disempowered, it’s not a nice feeling.” p 394 | X | |  |  | F161 |
|  | Ongoing feelings of continually being “let down”, as treatment fails and disability increases | **Patient:** “I’ve had chemo, I’ve had radiation but now they’ve decided they can’t do anything more so it’s just - up to the Gods. I’m just waiting. You know, you’re waiting, waiting, waiting, for things to happen and they just don’t.”  **Patient:** “I was supposed to be off those tablets [steroids] after 3 or 4 months. And I said well when are you going to give me back my licence, and they said oh 3 to 6 months, 6 months at the most. Well it’s been 6 months and nothing’s happened. Still on the tablets and still no licence.” P395 | X | |  |  | F162 |
|  | Unpredictability of illness meant patients couldn’t plan | **Patient:** “If you know what the odds are, you know how to plan – the annoying thing about this is it says, ‘All these things may, may, may.’ Nothing says can help, will help. I don’t know where I should be, where I’m supposed to be, where I’m predicted to be. I mean, how am I supposed to be feeling at this stage?”  **Patient:** “With my condition I don’t know how you could form a plan too much, because it could change like that.” p395 | X | |  |  | F163 |

| **Authors: 476 Piil et al. 2015** | | | |  |  |  |  |
| --- | --- | --- | --- | --- | --- | --- | --- |
| **Participants, Clinical and Demographic Characteristics** | **Findings** | **Illustrations (Page number)** | **Evidence** | | | | |
|  |  |  | **Unequivocal** | | **Credible** | **Unsupported** | **Finding Number** |
|  | Individual Strategy for Acquiring Prognostic Information (PI) | **Caregiver**: In no time I read all accessible information on the Internet. (Malcolm, 59 years old, caregiver, interview 1) Pg 274  **Patient:** I prefer to know about my prognosisVit helps me to be prepared. I spend time with my family planning and discussing my situation. (Steven, 71 years old, patient, interview 2) Pg 274  **Patient:** I have to keep the amount of information at a level I know I can deal with. It works for me. (Lauren, 33 years old, patient, interview 3) Pg 275  **Caregiver**: I don’t need more information, and if I do then I ask for it. I have a lively imagination and in order to prevent it from running away from me, I need to limit the information. That is the only way I can concentrate on my work...after having comes back and it’s pretty much enough. (Susanne, 64 years old, caregiver, interview 1)  **Caregiver** sorted the information I’m able to concentrate again. The more anxious I get, the harder it is to handle the situation with Steen and I’m really trying to...restrict it. I know the facts, I know it’s an aggressive cancer and that it comes back and it’s pretty much enough. (Susanne, 64 years old, caregiver, interview 1) Pg 275-276 | X | |  |  | F164 |
|  | Shared Hope | **Patient:** I will not die now. I will fight the best I can.... I will not just give up. You’ve heard of miracles. I have to live on hope don’t I? Yes, I do hope that I will survive. (Rita, 68 years old, patient, interview 1) Pg 277  **Caregiver** Since we don’t have a curative treatment, we will do everything else we can to survive. (Mary, 44 years old, caregiver, interview 2) Pg 277  **Patient:** I’ve decided that I need to handle this and I will fight this with a positive mind and then later I will enjoy my retirement quietly. (Bob, 62 years old, patient, interview 1) Pg 277  **Patient:** I hope that I can live for another 5Y6 years...6 years. (Heidi, 61 years old, patient, interview 1) Pg 277  **Patient:** I haven’t been sick before. it must be an advantage to me. (Tim, 29 years old, patient, interview 1) Pg 277  **Patient:** The physician predicted that I could live for another 2Y3 years, and at that time there would be a curative treatment there will, he said. It means a lot to me and I was much relieved to hear that. (Kenneth, 55 years old, patient, interview 2) Pg 277  **Caregiver** Instead of telling us that this is the end, you should say that it is a difficult situation. You ought to encourage us to use self-management strategies it is important. Whether he dies now or later it is his quality of daily life that is important. Support us by telling us what we can do. (Mary, 44 years old, caregiver, interview 3) Pg 277  **Caregiver** I do not think there is much hope at all. (Deirdre, 49 years old, caregiver, interview 4) Pg 277  **Caregiver** He (the patient) told me that he has no life. He could only stay at home not being able to do the things he used to. Then we took a very important and crucial decision. It felt right and I was calm. Our focus was then to get the best out of every day not being disturbed by any more scans. (Charlotte, 55 years old, caregiver, postbereavement interview) Pg 277 | X | |  |  | F165 |
|  | Engagement in Health Promotion Activities | **Caregiver** I guess the chemotherapy affects the immune system, so we try to strengthen it. (Lisa, 57 years old, caregiver, interview 2) Pg 278  **Caregiver** I’m sure that a diet can be life-sustaining, we try to make vegetable juices and right now I’m willing to try anything. (Lea, 38 years old, caregiver, interview 2) Pg 278  **Patient:** I don’t drink alcohol during the treatment with the radiation treatment; my body receives enough toxins. (Adam, 66 years old, patient, interview 2) Pg 278  **Patient:** I exercise at least 5-6 hours per week. At the rowing club I meet other people and friends and that is important. I prefer to exercise with people I know. We have a cup of coffee and talk. I appreciate talking with themVit is important to me. (Peter, 50 years old, patient, interview 2) Pg 278  **Caregiver** If our friends read or hear something that could be useful they send us an e-mail. (Jacob, 54 years old, caregiver, interview 4) Pg 278  **Caregiver** I’ve fed him broccoli, and I buy fresh blueberries every time I can find any. He eats a lot of ginger and garlic and things like that. I also heard that chili is good. That’s why I serve him food with these ingredients. Occasionally, he is sick and tired of garlic and ginger, but he needs to eat whatever I cook for him. He has refrained from alcohol consumption and sugar for a very long time, but suddenly he starts eating sweets. Afterwards he feels guilty. However, he still refrains from alcohol. (Anny, 56 years old, caregiver, interview 3) Pg 278  **Caregiver** Since I’ve been shopping at the health food stores we have completely changed our diet we have a positive attitude. (Mary, 44 years old, caregiver, interview 1) Pg 278  **Caregiver** We have increased our effort and gained more knowledge about diets. Now we know about the plus and minus balance (special diet). We were told that we are both in minus and need a plus diet. Therefore we do even moreVit is expensive though. (Luisa, 68 years old, caregiver, interview 5) Pg 278  **Caregiver** We use intravenous C-vitamin as a supplement because it can have the same effect as chemotherapy. (Mary, 44 years old, caregiver, interview 1) Pg 278  **Patient:** I buy shark’s liver oil in Sweden. I also used it the last time I had cancer. I don’t know if it helpsVbut I feel it does. (Betty, 69 years old, patient, interview 2) Pg 278  **Patient:** I only receive the injections with C-vitamin once every 14 days. It costs 167 USD per session. I’ll been there 70 times. I felt like a complete fool spending that amount of time. I went there 2 times per week and it was too much. (Adam, 66 years old, patient, interview 5) Pg 279  **Patient:** I don’t use shark’s liver oil anymore. I didn’t think it had any effect. (Betty, 69 years old, patient, interview 5) Pg 279 | X | |  |  | F166 |
|  | Adjustment to Symptom Limitations | **Patient:** I’m self-employed and thought it was a sign of stress. I went to see a physiotherapist for massage. But I still felt sick. Then I developed visual disorders. The physician and I still related it to stress. I decided to close my firm. Shortly thereafter my legs couldn’t bear me and I was admitted for an acute brain scan. (Ken, 38 years old, patient, interview 1) Pg 279  **Patient:** I would have appreciated being introduced to other patients that were in a similar situation. (Ken, 38 years old, patient, interview 3) Pg 279  **Patient:** You don’t have the energy for anything. Now I don’t bother being together with others. I’m just drained of energy. (Ken, 38 years old, patient, interview 4) Pg 279  **Patient:** I’m another person now my wife has changed too. I’m afraid, that she takes on too much responsibility. I’m afraid that she can’t handle it and that makes me insecure. (David, 50 years old, patient, interview 1) Pg 279  **Patient:** I don’t dare to go for a run in the woods, because I’m having these seizures instead I try to go for a walk everyday. (John, 31 years old, patient, interview 2) Pg 279  **Patient:** My brain couldn’t tolerate all those different instructions.... I had to play ball with another person. Next, I should touch my toes, and all of a sudden...it was not feeling right. I’m not sure if I’m going to attend that rehabilitation program again, because of my head is feeling crazy. I have some fluid that accumulates there. (Sheila, 71 years old, patient, interview 4) Pg 279  **Patient:** I feel that something is wrong, which is why I agreed with my wife that she reads through everything I’ve writtenVin order to identify any possible errors. If I write something during the afternoon or evening, I’m very surprised to learn how bad it reads the following day. (Steen, 79 years old, patient, interview 4) Pg 279  **Patient:** Work was too difficult. I was very reluctant to give it up, but I had to. I made too many mistakes. (Lily, 53 years old, patient, interview 5) Pg 280  **Patient:** At the moment, I need to be guarded again. I’m making all sorts of errors and I need to consider all tasks very carefullyVeven daily activities. (Hans, 50 years old, patient, interview 4) Pg 280 | X | |  |  | F167 |
|  | Role Transition From Family Member to Caregiver | **Caregiver** One should not give up. We have been married for 46 years, so why shouldn’t I help her at this stage as long as I have the strength. (Joe, 67 years old, caregiver, interview 3) Pg 280  **Caregiver** I keep my own logbook. I have a responsibility for ensuring that they (health care professionals) have the required information in order to make decisions. If not we risk that they use outdated information. (Stuart, 54 years old, caregiver, interview 4) Pg 280  **Caregiver** I need to do everything; earn money, cook, be a good hostess, be the driver and care for his needs. I need to do everything, don’t I? (Susanne, 64 years old, caregiver, interview 3) Pg 280  **Caregiver** I have to help him with everything and I’m constantly aware of what he is doing 24/7. (Brianna, 46 years old, caregiver, interview 1) Pg 280  **Caregiver** Jackson cheats them (health care professionals) at the medical consultations. He pretends to appear better than he actually is and it frustrates me a lot.... he just says that everything is fine and if I don’t agree, he gets very upset. They seem to believe him when he tells about going hunting with his good friend.... they cannot see how bad he really is. (Hannah, 44 years old, caregiver, interview 5) Pg 280  **Caregiver** I’m so tired that I even speculate in my sleep that is new to me. (Mary, 44 years old, caregiver, interview 2) Pg 280  **Caregiver** He (the patient) goes to bed early and I can’t attend any of my own activities. I’m bound ‘‘hand and foot’’ by this disease. If only I could have a break from this life just for 2 days. (Hannah, 44 years old, caregiver, interview 5) Pg 280  **Caregiver** He can’t remember that the prognosis is poor. It is terrible. He refrains from discussing all of these issues. It is his way of getting through this, and we can’t share those things anymore. (Deidre, 49 years old, caregiver, interview 4) Pg 280  **Caregiver** His is like another husband. Everything has changed. Maybe it is because of the steroids, butII just think that he is acting very manic. Maybe he is bored just being at home, and then he does strange things that he has never done before. And then he asks me to do this and that...it is strange, and it’s hard to know when to...put the brakes on, and when to...you know, how nice should I be, so to speak? (Lisa, 57 years old, caregiver, interview 3) Pg 281 | X | |  |  | F168 |

| **Authors: 427 Raju & Reddy 2018** | | | |  |  |  |  |
| --- | --- | --- | --- | --- | --- | --- | --- |
| **Participants, Clinical and Demographic Characteristics** | **Findings** | **Illustrations (Page number)** | **Evidence** | | | | |
|  |  |  | **Unequivocal** | | **Credible** | **Unsupported** | **Finding Number** |
|  | Understanding about illness | **Patient:** “I know I am admitted for brain tumour treatment as informed by treating doctor. Apart from that, I do not know in detail; what is brain tumour? Why does it come to me? I do not have any bad habits like smoking and drinking. I want to know about my illness. Even my relatives divert on the same whenever I ask them”  **Patient:** “What I am aware of is, I have been suffering with brain cancer. I had already undergone surgery, radiation, and chemotherapy once but till today, I do not know, why I should take these many treatments for my illness” | X | |  |  | F169 |
|  | Personal views and feelings on death and dying | **Patient:** I feel sad… sometimes, I get fear whenever I think about my death and even future of my family members”  **Patient:** “I may die soon, I am not worried for myself. I am thinking of my children and wife. Who will look after them if I die…. I am worried more for them….(Cried…….)… (After long breath)….It’s better to die than creating burden to wife and children”  **Patient:** “I feel worried, not only me everyone will feel fear…worried…if they know, they are dying….(…pause…. silence……for….4min…. tears…. observed in the eyes….). I would like to die at home, if I die with my illness” p 321 | X | |  |  | F170 |
|  | Coping with fears of death and dying | **Patient:** “I get death‑related thoughts wheneverI am alone. That time, I pray to the God and chant Mantras. Sometimes, I talk to my wife to get rid of those thoughts”  **Patient:** “I know I may die soon with my brain cancer. My family is good support for me. They always be with me and help me in day–to‑day activities. I call and talk to them and divert my death‑related thoughts”.  **Patient:** “My family members have to consider me as I am before my illness. They should not treat me as ill person. I ask them only one cigarette in a day, I like cigarettes…(smiled….)…nothing more I need from them during my last days.” p 322 | X | |  |  | F171 |

| **Authors: 240** Sterckx et al (2015) | | | |  |  |  |  |
| --- | --- | --- | --- | --- | --- | --- | --- |
| **Participants, Clinical and Demographic Characteristics** | **Findings** | **Illustrations (Page number)** | **Evidence** | | | | |
|  |  |  | **Unequivocal** | | **Credible** | **Unsupported** | **Finding Number** |
|  | Devasting experience of living with High Grade Glioma (HGG) – diagnosis is a shock; leads to uncertainty and anxiety, feelings of loss and disregard. | **Patients:** “In the beginning you don’t believe it (the diagnosis), because you don’t feel sick. You don’t know it, it is later that you come to realize, much later. **(p. 385)**  **Patients:** “When everyone is gone and you sit on the sofa, then the fear comes back. Then, you start to cry. I still have that, I know for now that the tumour shrunk two times, but still, sometimes the fear returns…The fear about ‘What if the therapy stops working’. Some days it comes and other days…’ **(p385)**  **Patients:** “That nasty cancer. Why does it expand and why does it destroy me, little by little, more and more?...You are only a part of the person you once were, anymore…” **(p 385)**  **Patients:** “I simply cannot do anything about this disease. I find it terrible that I cannot do something about it. I’m used to helping myself a little and in this case I can’t **(p.386)** | X | |  |  | F172 |
|  | Patients’ inner strength that keeps them going | **Patients:** “The little things, I’m happy with just the little things. First it didn’t work out and now I can do some things again. I changed a switch and repaired a bicycle…” **(p386)** |  | | X |  | F173 |
|  | Caring needs  Receiving help is a support but also a burden  Patients wanted information about their disease and what to expect  Needed to share their emotions and concerns  Accessibility and availability of professional caregivers was essential | **Patients:** About getting offered a hospital bed, “I say, not yet. I don’t need it. When there is a bed…then I’m lost; then i have nothing to go upstairs for…” **(p. 387)**  **Patients:** **“**But I can ask him everything; I get trustworthy information, an answer. I found it very important that you are fully informed” **(p. 387)**  **Patients:** “There was no frank discussion about what I have and what it means…and it is cowardly to avoid speaking about it **(p. 387)** | X | |  |  | F174 |

| **Authors: 189** Tastan et al (2011) | | | |  |  |  |  |
| --- | --- | --- | --- | --- | --- | --- | --- |
| **Participants, Clinical and Demographic Characteristics** | **Findings** | **Illustrations (Page number)** | **Evidence** | | | | |
|  |  |  | **Unequivocal** | | **Credible** | **Unsupported** | **Finding Number** |
|  | First reaction: shock and fear of death | **Caregiver:** “It was awful. At first we thought it was bleeding in the brain. I could not believe it and felt faint when I learnt it was a brain tumour. I was unable to think anything. **(p.80)** | X | |  |  | F175 |
|  | Decision for surgery: Helpless acceptance | **Caregiver:** “He had to undergo a surgery. This was his only chance to live. He is a young man and he must have many years to live. So, we had to accept the surgery.”**(p.80)** | X | |  |  | F176 |
|  | First Meeting with the patient after surgery: happiness or fear | **Caregiver:** “I kept thinking of death until the surgery was over and I saw her. ‘What if something happens during surgery?’ was my fear. I would not hold back my teas when the surgery was over and I saw my mother.” **(p.80)** |  | | X |  | F177 |
|  | Management of the side effects of the tumour | **Caregiver:** “My spouse’s movements were slow after the surgery. He had difficulty when walking, eating and using his hand. This really bothered us. We did not know what to do.” **(p.81)** | X | |  |  | F178 |
|  | Management of role and behavioural changes | **Caregiver:** “The behaviour of my wife annoyed me. She became aggressive sometimes. She blamed me for everything. She accused me even if anything was lost in the house.” **(p.81)** | X | |  |  | F179 |
|  | Management of care in the home | **Caregiver:** “At that time, my wife had nausea and anorexia. She lost weight. She should have eaten something but she could not. We slogged away in this situation.” **(p.81)** | X | |  |  | F180 |
|  | Social support – carers made life adjustments to provide support. Some received support from family members, friends or neighbours. | **Caregiver:** “It was very difficult. Everything was planned according to my son. We could not do anything. But we thought that it was okay as long as my child was well. It was not important even it we were in pain and could not do our work.” |  | | X |  | F181 |
|  | The experiences of patients were different. Half had felt the information provided was sufficient. The other half did not. | “**Caregiver:** They just made you sign a paper and then operate. It was going to be serious surgery. They did not provide good, detailed information. They were a lot of unexplained things. I did not know what to ask, who to ask.”  **Caregiver:** The information provided to me was adequate. Everything was said. We knew everything that could happen. We did not experience anything other than what was said. **(p. 82)** | X | |  |  | F182 |

| **Authors:** 130 – TinaWang 2018 | | | |  |  |  |  |
| --- | --- | --- | --- | --- | --- | --- | --- |
| **Participants, Clinical and Demographic Characteristics** | **Findings** | **Illustrations (Page number)** | **Evidence** | | | | |
|  |  |  | **Unequivocal** | | **Credible** | **Unsupported** | **Finding Number** |
|  | Information needs | **Patient**: During a consultation, the neurologist urged the participant to ask questions and asked whether there were any he wanted to ask right now, and the participant replied: “Yes, I do have some questions, but I have forgotten it all in a minute anyway”.  **Patient:** He explained it well and he said what he could do and didn’t promise to do more than he could. And I think that is really fine, that they don’t promise more than they can keep. And they don’t look at the dark side either. |  | | X |  | F183 |
|  | Balancing hope and reality while trying to perceive the unknown reality of brain cancer | **Patient:** “Well, she did say they had found a lump and that they cooperate with [name of hospital] why I have to go there” (I: Did she tell you, what they suspected it to be?) “No, she did not” (I: Did this conversation make you any wiser?) “No”. |  | | X |  | F184 |
|  | Not knowing what to expect | **Patient:** The first neurologist who this participant met, chose to describe the result of the MRI saying: “You had a scan yesterday...[pause] and we have found a tumour in your brain. It looks malignant.” After this consultation, the result of the CT TAP scan being normal was provided to the participant by a second neurologist who articulated the brain tumour as: “You have something in your head.” When the participant was evaluated by a neurosurgeon, he was shown the MRI and the neurosurgeon said: “You have something there, there and there and we really do not know what it is or if it requires after treatment. We can take a sample of it, and we can do that already tomorrow.” As the spouse asked, “do we know if this is cancer?”, the neurosurgeon replied: “We cannot leave out that option. ”When this participant was interviewed about his understanding of the neurosurgery consultation just afterwards, he answered, “I only understood that; it is the operation you know. They do not know what it  is...”. On the actual day of the operation, the neurosurgeon who was to perform the surgery, met the participant for the first time and they talked for five minutes. During the brain surgery, the neurosurgeon stated to the first author: “Isn’t he funny? When I talked to him, I had no impression of him having received any information prior, even though the medical journal from yesterday clearly states, highly malignant, and one can see, it has been said during every consultation that this is what we expect.” |  | | X |  | F185 |
|  | Perception of relationship with health workers | **Patient:** We were having an equal dialogue. It was not like “I know-it-all”, “..also, he sits down himself, hmm, and he is present, right? And he, he does not have to think about, “what am I to say now,” he already thought that through! That is how I experienced it. He had prepared what he wanted to say.  **Patient:** This made the participants feel reassured, “... it was a good conversation. He is a pleasant doctor; he was nice and made me calm”  **Patient:** Even though participants met several healthcare providers in each department, they all were able to remember the names of the ones who they felt, had “seen and understood” them.  **Patient:** Participants also felt disappointed when healthcare providers gave vague answers or were unable to answer their questions.  **Patient:** No, but the first [consultation] was just a chit-chat. It made no difference. I could damn just as well have talked to my general practitioner. He knew just as much, I think. No, then you are too young and inexperienced to do.... No, I don’t know. I was not impressed by that. |  | | X |  | F186 |
|  | Being alone surrounded by health-care providers | **Patient:** It is always hard when they (HCPs) ask that “do you have any questions?”. It is so confusing, isn’t it? What am I to ask? They (questions) don’t appear until later on, when you have thought of what they said and at that point they (HCPs) have left.  **Patient:** In general, coordination of care was a huge challenge to Susan during the rigid treatment schedule of 30 days of radiotherapy. Due to a need for flexibility in the Department of Oncology, Susan only received the appointments for radiotherapy for 1 week at the time, which made it hard for her to plan anything ahead:  **Patient:** It is a little irritating; in fact it is very hard . . . I attend this rehabilitation and yesterday, she (physiotherapist) could not plan anything, as long as I did not have any appointments for radiotherapy, could she? So she is going to call me this afternoon.  **Patient:** Susan also experienced lacking what seemed to be “inherent” knowledge of the healthcare system. She described it as knowledge; she could not imagine she would need it until the situations actually occurred:  **Patient:** In the future, I must remember to have the blood sample taken first (before radiotherapy treatment), because the lab closes at 2 pm. That is what I should have done today, but no one have told me they close at 2 . . .I called the hospital last night, just as they told me to, if I have a fever, but when I called they said 37.8 was not to be considered fever. Apparently, I am only supposed to call them if I have 38.5, but no one told me that. |  | | X |  | F187 |
|  | Developing strategies to manage in the health-care system | **Patient:** “I hardly can remember what she (the nurse) said . . . but I think (name of spouse) can.”  **Patient:** In the same way, Susan described how she often experienced being asked questions such as, “When is your appointment for radiotherapy tomorrow?” or “When was your corticosteroids reduced to 16 mg?”  **Patient:** When I talk to them, they do not know, they start asking you questions instead of . . . as if they have not read the medical record, right? (I: Do you mean, when they ask you of what medication you are taking . . .?) Yes, they all ask that question, and the nurses do it too . . . and it bloody cannot be right, can it? That I have to run around with my notes? What if I became all dotty . . . ? | X | |  |  | F188 |

| **Authors:** Vedelo et al 2019 | | | |  |  |  |  |
| --- | --- | --- | --- | --- | --- | --- | --- |
| **Participants, Clinical and Demographic Characteristics** | **Findings** | **Illustrations (Page number)** | **Evidence** | | | | |
|  |  |  | **Unequivocal** | | **Credible** | **Unsupported** | **Finding Number** |
|  | Wanting to know it all | **Patient:** “It was nice to get the facts on the table…concrete and direct information…I wanted to have the facts straight. I wanted to know it all, so I can relate to it.” (p30) | X | |  |  | F189 |
|  | Feeling vulnerable and alone | **Patient:** Facing an unknown future made Susan feel alone and out of control, and she said, “I just want to know the course of events…”(p.30) |  | | X |  | F190 |
|  | Navigating the System | **Patient:** “In the future, I must remember to have the blood sample taken first (before radiotherapy treatment), because the lab closes at 2pm. That is what I should have done today, but no one have told me they close at 2…”(p31) | X | |  |  | F191 |
|  | Multiple Healthcare Providers and Settings | **Patient:** “I am so tired of seeing so many new people all the time…I cannot relate to that…I don’t even notice their names anymore…there are so many of them…now I actually only notice the doctors (p31-32) | X | |  |  | F192 |
|  | Disagreement of Healthcare Providers | **Patient:** “They all have an opinion of their own. It is strange and I mean they really must have the same education, don’t’ they? The doctors, they don’t read my medical record the same way I think…(p33). | X | |  |  | F193 |
|  | Not Being Heard and Feeling like a Burden | **Patient:** “I was sweating one night, I was wet all over, so I asked if I could have a new t-shirt, I got that, then I asked if I could have another one (t-shirt) and no, this time I could not, it is not that wet, they said to me, you can wear it until the morning, they should just try having sweats and feel so rotton, right? They would not give me a new one, no…I don’t know if they were busy…they would not give me a new one, she said it straight to me…yes, that made me a little speechless.”(p. 33) | X | |  |  | F194 |
|  | Providing Documentation | **Patient:** “I carry it with me (the print of prescribed medication) everywhere I go, so, they are able to see it.” | X | |  |  | F195 |
|  | Anticipating Questions and Tasks to Come | **Patient:** “I always have them (letter and paper from the hospital) with me, yes. I have to, really because I cannot remember, can I?”  (p.35) |  | | X |  | F196 |

| Wasner 2013, | | | |  |  |  |  |
| --- | --- | --- | --- | --- | --- | --- | --- |
| **Participants, Clinical and Demographic Characteristics** | **Finding** | **Illustration** | **Evidence** | | | | |
|  |  |  | **Unequivocal** | | ***Credible** | **Unsupported** | **Finding Number** |
|  | Psychological Distress and Burden of Care | **Caregiver:** I’ve been doing this whole thing for 18 months now. Sometimes I don’t believe it myself, all the things I’ve done. The worst is, that sometimes he’s so aggressive, so much changed from the feeling and sensitive person he used to be. Sometimes I think I don’t matter to him anymore. And he doesn’t really understand the condition he’s in, he still thinks he can do everything by himself. | X | |  |  | F197 |
|  | Taking Responsibility | **Caregiver:** Of course the result is, of course, that I have no private life to speak of … . But I think, my mother needs me now and, ah, like I said, first of all, it’s a diversion, it helps me and it’s my mission, it’s there to be fulfilled and doing it is a pleasurable fulfillment and yeah, it’s like that. It’s not as if I’m falling into some kind of depression or something, I think, that’ll come later, now is not the time. (45-year-old woman, child)  **Caregiver:** Yeah, I would say that my daily chores, no matter how basic or demanding, and also the decision making, all that has doubled in the last 4 years. Actually, I mean, I work for two and, in many ways, live for two. (41-yearold woman, spouse) | X | |  |  | F198 |
|  | Recognizing the Significant Role of PMBT Caregivers | **Caregiver:** The neurologist here said to me, “Be happy, (if) you don’t know what’s going on, then you won’t have to get upset.” I raged when I heard this; then I already knew through the Internet that it could be a Glio IV and it was pretty clear to me what the consequences would be. It was totally idiotic to say to me, “Don’t worry.” That was the worst, this information block. They were incapable of making even a single statement. (53-yearold woman, spouse)  **Caregiver:** One of the main problems appears to be that PMBT patients are not necessarily capable of perceiving their physical and mental condition adequately. One caregiver described this aspect:  **Caregiver:** I’m always confronted with these situations, he can no longer responsibly decide for himself, doesn’t know which medication he should take, when, or even how. Suddenly he decides to stop taking steroids. I said to him, do you know what the doctors (thank God I was there) said, what Prof. XY said? I had to work on him but finally he said: “Ok, I’ll take them.” I told him, you’re the patient now and you’ll do what he said. He’s doing it, too. It’s an enormous burden for me that I’m practically co-responsible for him and then he feels like I’m handling him like a child. Often, I’m overambitious and tell him things he knows. (56-year-old woman, spouse) | X | |  |  | F199 |
|  | Need for Solid and Continuous Support | **Caregiver:** He was fresh out of surgery and, instead of talking directly to his wife; the doctors gave him the information about his condition for him to tell his wife. If not even the doctors in such a ward know that directly after an operation the connection to reality is estranged and not totally functioning and that you no longer can rely on it, then I don’t know who should. And then, I have to say, why don’t they just write a note and lay it on his night table, “Mrs. XY, please contact the nurses when you arrive.” Unfortunately, they don’t do it like that, instead they talk directly to the patient who’s not capable of understanding the information and who twists and turns the issues so that when my mother does meet with the doctors, there’s total confusion. She didn’t contact the doctors earlier because no one encouraged her to do so. (35-year-old woman, child)  **Caregiver:** Yeah, and they don’t commit themselves at all, they only made short speeches, … perfect operation, the nurses were very nice, everything was well organized but in spite of everything, you feel immensely let down and alone. It’s the time of … It was even worse in the neurology department, we felt totally neglected there because the doctors said absolutely nothing. I: You got the diagnosis before the operation? A: Yeah yeah, we talked 3 minutes with a senior physician instead of the head physician. He told us, “According to the biopsy, it’s Grade II to Grade III,” that was it. He recommended an operation and then he was gone. This was hell. At this stage my husband couldn’t understand everything but for me this was hell. For him the most important thing was that he could walk again, and then he wouldn’t have any more problems. (53-year-old woman, spouse) | X | |  |  | F200 |
|  | Practical Advice and Help | **Caregiver:** The caregivers are interested in practical advice. In many cases, the need for practical advice is connected with economic circumstances. One caregiver described this aspect, “And soon you have the financial problems. Then/ while in the moment we get some sick-pay, but soon it will be over and then it is applying for welfare aid” (35-year-old woman, spouse). According to another participant:  **Caregiver:** Hmm, yeah, well for me, I just can’t understand it all. Why don’t they make a checklist or something like that which you could use to take care of stuff step by step? Especially in the state we’re in now. Everyone always asks, “What’s his nursing insurance level?” Well, when we answer, none, and ask, how do we get one, from whom and where, what do we have to do to get it, the answers we get are just excuses and false promises, like, “Yeah, they do that or, we could also do this for you,” but nothing is ever done and no one ever told us, “Come on, let’s get you some help.” (35-year-old woman, child) | X | |  |  | F201 |

| Whisenant 2011, | | | |  |  |  |  |
| --- | --- | --- | --- | --- | --- | --- | --- |
| **Participants, Clinical and Demographic Characteristics** | **Finding** | **Illustration** | **Evidence** | | | | |
|  |  |  | **Unequivocal** | | **Credible** | **Unsupported** | **Finding Number** |
|  | Enduring responsibility  Caregiver feelings of responsibility for  the welfare of the patient that extend  over time, despite difficulties or challenges that may arise | **Caregiver:** A 68-year-old husband of a patient expressed his desire to meet the challenges ahead: “It is a role that I relish because I wouldn’t want anybody else to be that caregiver. . . . Whenever that tumor was diagnosed, I knew . . . [we] were going  to have to face this together.” | X | |  |  | F202 |
|  | Making the patient priority  Placement of patient needs before all others; often requires significant life changes made for patient’s welfare | **Caregiver:** A 53-year-old mother described a difficult decision made in the best interest of her son; although she wanted to spend every moment with her son, she chose to continue working to support the family |  | |  | X | NS |
|  | Self-affirming, loving connection  Caregiver feelings of connectedness  with the patient, where meeting patient needs is emotionally satisfying for the caregiver | **Caregiver:** The 62-year-old wife of a patient described the importance of her role as a caregiver: “In some ways it is better because you are closer now than you were, and I feel sorry for him mostly because he had a wonderful brain, and a wonderful  memory, and to see someone lose that, but he seems happy [and] that is the main thing. . . . He is so grateful to me for helping him.” | X | |  |  | F203 |
|  | Supportive presence  Remaining at the patient’s side with  comfort, encouragement, and a positive attitude when the caregiver can  do nothing else for the patient | **Caregiver:** A 50-year-old husband caring for his wife described his support for her: “You have got to be very supportive because their mind changes after a brain tumor. . . . Sometimes she would say . . . she is sorry she is such a hard patient to work with.  I guess . . . you have to remember that love is the thing that brought us together and our love and our faith in God is what is keeping us going.” | X | |  |  | F204 |
|  | Envisioning tomorrow  Struggling with an unknown future involving hope, fear, or both to help caregivers find purpose and prepare for disappointments | **Caregiver:** A 40-year-old wife caring for her husband described both a hopeful and fearful outlook on the future: “What I hope to happen and what is going to happen are two different things. I hope they can contain the growth . . . and he could just  live another two to three years . . . but it looks like it is going to be six months. It is a monster tumor and it grew through radiation and [chemotherapy], so it  is going to be much more difficult now that he understands that he is dying,  because he had a lot of hope.” | X | |  |  | F205 |
|  | Gauging behavior  Explaining, predicting, or reacting to the patient’s actions or statements based on previous knowledge and experience | **Caregiver:** A 50-year-old husband caring for his wife described anticipating his wife’s needs: “I have been doing it [caregiving] with her so long now that I kind of know what needs to be done before she asks [and] I . . . anticipate what she will be wanting or needing.” | X | |  |  | F206 |
|  | Getting back to normal  Anticipating the return of an ordinary life that was lost in the demands of illness and treatment | **Caregiver:** A 52-year-old husband caring for his wife described a hope for a return to normal life: “I would like to have a sense of normalcy. . . . I know that it might be kind of a false sense of normalcy, but a feeling that . . . at least things are  good for now.” | X | |  |  | F207 |
|  | Reconciling treatment twists and turns  Comparing actual to anticipated patient outcomes to accept the reality  of the outcome | **Caregiver:** A 28-year-old woman caring for her sister told of accepting the reality of the outcomes of treatment: “I am not all that medically inclined, but [I] try to work with her and help her understand that if this procedure don’t work, there is going to be another one . . . and that one might be a little bit better than the first one or it might be a little worse than the first.” | X | |  |  | F208 |
|  | Taking one day at a time  Focusing in the present as a means of dealing with an ambiguous future that cannot be envisioned | **Caregiver:** A 38-year-old wife caring for her husband described dealing with illness by living one day at a time: “His favorite saying all along . . . is, ‘None of us know what our date is on our contract.’ . . . It is obvious that we are just supposed  to live it one day at a time.” | X | |  |  | F209 |
|  | Appropriate pushing  Caregiver taking responsibility to ensure that the rules for recovery set by healthcare providers are followed | **Caregiver:** A 74-year-old wife described taking responsibility and enlisting the support of  her son to convince her husband to take his medications, despite his disinclination to do so. |  | |  | X | NS |
|  | Attending to patient voice  Describes caregivers listening and considering patient perspectives before deciding on a course of action | **Caregiver:** A 50-year-old husband described listening to his wife’s perspective: “If I had tried  to pressure her into signing up for disability, that would have been a bad deal. So I  didn’t pressure her . . . and she made up her mind that she was going to [apply].” | x | |  |  | F210 |
|  | Getting a handle on it  Coming to grips with the reality of the demands of illness and identifying strategies to meet caregiving demands | **Caregiver:** A 53-year-old wife caring for her husband described getting a handle on the new diagnosis and new responsibilities: “This has been [our] first experience with cancer . . . so dropping into brain cancer has been sort of jumping into the deep  end of the pool. What has been difficult for me has been the amount of additional  work that fell in my lap: taking care of financial things, taking care of insurance,  taking care of the worries of all of that.” | X | |  |  | F211 |
|  | Sharing responsibilities  Determining caregiving needs and dividing responsibilities between people such as the caregiver, patient, healthcare provider, and family and  friends | **Caregiver:** A 32-year-old daughter caring for her father discussed sharing caregiving responsibilities: “This is just way over my mother’s head. I have a pill organizer and I fill  it up. . . . She doesn’t even know what those pills are.” | X | |  |  | F212 |
|  | Cultivating healthy habit  The caregiver maintaining or improving their own health to meet the caregiving demands | **Caregiver:** A 53-year-old mother caring for her son described taking care of her own health: “I am taking some vitamins and eating very well so I can be strong.” | X | |  |  | F213 |
|  | Getting away from it  Finding space to temporarily experience ordinary life away from the demands of caregiving | **Caregiver:** A 53-year-old wife caring for her husband described getting away from her caregiving responsibilities on occasion: “I really tried to keep my life going. . . . Every now and then I do get to a concert or I go to see friends, and I know that part is  real important for me.” | X | |  |  | F214 |
|  | Letting it out  Expressing the feelings and frustrations  associated with caregiving | **Caregiver:** A 28-year-old woman caring for her sister described expressing her frustrations:  “She needs help, and I often tell [my family], ‘She is going to have these mood swings, that is what drugs do to you . . . you have to be patient. You might have  to walk out of the room, grit your teeth, go outside and scream and holler, but come back.’ That is what I do sometimes.” | X | |  |  | F215 |
|  | Supportive physical environment  Creating accommodations, food, and  other amenities that are comfortable  and convenient for caregiving | **Caregiver:** A 32-year-old daughter caring for her father told of creating a physical environment amenable to being able to stay with her father at all times by keeping an  inflatable mat in her car. |  | |  | X | NS |
|  | Experiencing personal growth  Gaining new perspectives, knowledge,  and skills in the caregiving experience | **Caregiver:** A 53-year-old husband caring for his wife exhibited new perspective: “The good  thing about it is I think we have learned to appreciate each day.” | X | |  |  | F215 |
|  | Leaning on the Lord  Finding comfort and strength in the  belief that a higher power has control  of the situation | **Caregiver:** A 48-year-old husband caring for his wife described leaning on the Lord: “We both prayed and said, ‘God, you are going to have to help us with this one, this is bigger than us,’ and when we turned it over, it is not a burden.” | X | |  |  | F217 |
|  | Recognizing positive outcomes  Being uplifted by events that signify to  the caregiver an improvement in the  patient’s health | **Caregiver:** A 53-year-old husband described how surgery led to a perceived improvement in his wife’s health, giving her immediate relief and uplifting him. |  | |  | X | NS |
|  | Encountering competent, compassionate  care  Finding healthcare personnel who  meet the needs of both the patient  and caregiver | **Caregiver:** A 53-year-old wife caring for her husband described finding strength in competent  medical care: “One of the things that . . . made it easier for me to handle was  coming here and [letting go] of worries about the medical treatment. . . . That is  a huge thing [to] have that confidence.” | X | |  |  | F218 |
|  | Finding support for other responsibilities  Caregivers accessing assistance from  other people to handle responsibilities  not related to caregiving | **Caregiver:** A 32-year-old daughter caring for her father told of having her mother watch her  children one day a week so she could share the day with her father. |  | |  | X | NS |
|  | Knowing others care  Feeling emotional support from people outside the caregiving dyad, which  gives the caregiver a sense of personal  value and worth | **Caregiver:** A 48-year-old husband caring for his wife said, “We have a strong family support, we have a strong church support, and it is all part of the equation.” | X | |  |  | F219 |
|  | Meeting financial obligations  Finding ways to pay for added expenses of health care while compensating for lost income. | **Caregiver:** A 50-year-old husband caring for his wife described their decision to apply for disability |  | |  | X | NS |
|  | Receiving helpful information  Acquiring the knowledge needed to  perform as a caregiver | **Caregiver:** A 32-year-old woman described the process of gaining information: “Today I  brought my tape recorder and I asked the doctor some questions.” | X | |  |  | F220 |

Unequivocal (findings accompanied by an illustration that is beyond reasonable doubt and; therefore not open to challenge);

Credible (findings accompanied by an illustration lacking clear association with it and therefore open to challenge)

Not Supported (findings are not supported by the data)
